# Exploring the therapeutic potential of baicalin against MCF-7 breast cancer cells: biochemical, *in vitro*, and computational perspectives

**DOI:** 10.3389/fphar.2025.1698631

**Published:** 2026-01-09

**Authors:** Adil Ali, Tarun Kumar Upadhyay, Lamya Ahmed Al-Keridis, Mohammad Y. Alshahrani, Nawaf Alshamamri, Mohd Saeed

**Affiliations:** 1 Department of Biotechnology, Parul Institute of Applied Sciences and Research and Development Cell, Parul University, Vadodara, India; 2 Biology Department, Faculty of Science, Princess Nourah Bint Abdulrahman University, Riyadh, Saudi Arabia; 3 Department of Clinical Laboratory Sciences, College of Applied Medical Science, King Khalid University, Abha, Saudi Arabia; 4 Department of Biology, College of Science, University of Hail, Hail, Saudi Arabia

**Keywords:** baicalin, breast cancer, apoptosis, network pharmacology, molecular docking, molecular dynamics simulations

## Abstract

**Background:**

Baicalin (BA), a flavonoid extracted from the dried roots of *Scutellaria baicalensis*, is well known in traditional Chinese medicine (TCM) for its anti-apoptotic, anti-inflammatory, and antioxidant activities. However, its therapeutic potential in breast cancer needs to be evaluated systematically.

**Methods:**

In this study, BA was isolated by probe sonication, bath-assisted sonication, and maceration. Chemical and structural characterisation was done by UV–VIS, Fourier-transform infrared spectroscopy (FT-IR), high-performance thin-layer chromatography (HPTLC), high-performance liquid chromatography (HPLC), liquid chromatography–mass spectrometry (LC–MS), X-ray diffraction (XRD), and nuclear magnetic resonance (NMR), while morphological and physicochemical attributes were evaluated by scanning electron microscopy (SEM), zeta size, and zeta potential analysis. Antioxidant activity was measured by 2,2-diphenyl-1-picrylhydrazyl (DPPH), ferric-reducing antioxidant power (FRAP), ABTS, reducing power, and H_2_O_2_ scavenging assays. Total flavonol, flavonoid, and phenolic contents were measured, and biocompatibility was examined through a haemolysis assay. Anticancer activity was evaluated *in vitro* in MCF-7 breast cancer cells through 3-(4,5-dimethylthiazol-2-yl)-2,5-diphenyltetrazolium bromide (MTT), neutral red uptake (NRU), DCFH-DA, staining assays (Hoechst, propidium iodide (PI), LysoTracker, MitoTracker, JC-1, acridine orange/ethidium bromide (AO/EtBr)), and wound healing assay. Mechanism investigations were carried out using network pharmacology, docking, and 500 ns molecular dynamics simulations.

**Results:**

BA possessed good antioxidant activity and biocompatibility. Phenolic (99.9 ± 0.27 mg/g GAE), flavonoid (80.4 ± 2 mg/g QE), and flavonol (79.4± 2 mg/g QE) contents were concentration-dependent. BA revealed strong cytotoxicity towards MCF-7 cells (IC_50_ = 160 μg/mL) compared to 5-fluorouracil (IC_50=_410 μg/mL), whereas it demonstrated low toxicity towards normal fibroblasts (IC_50=_2 mg/mL). Cell-based assays showed induction of apoptosis, ROS production, mitochondrial and lysosomal disruption, and inhibition of cell migration. Docking and simulations also demonstrated stable binding with carbonic anhydrase IX (CA9), dihydrofolate reductase (DHFR), and matrix metalloproteinase 1 (MMP1).

**Conclusion:**

BA demonstrates strong antioxidant and selective anticancer activity with multi-target potential, supporting its promise as a natural therapeutic candidate for breast cancer.

## Graphical Abstract

**Figure:**
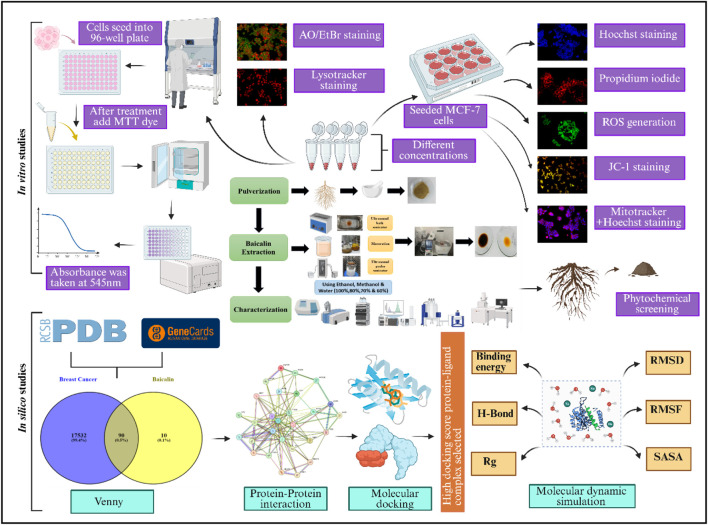
Graphical abstract showing baicalin extraction, characterisation, and various activities. The graphical abstract was created using BioRender software (https://biorender.com/).

## 1 Introduction

Breast cancer (BC) is the predominant form of cancer among women worldwide. BC is a complicated illness with various cell types, making prevention difficult worldwide. One of the most effective ways to avoid BC is through early detection (Sun et al., 2017). Based on the GLOBOCAN, there were 20 million newly diagnosed cases of neoplasms worldwide in 2022, with a mortality of 9.7 million cases. There were a total of 2.3 million BC cases and 0.6 million mortalities in BC overall; BC is the second leading malignancy in the world and the fourth leading cause of mortality. In Asia and India, BC is also the first leading cancer (Huang et al., 2019). Age, a personal or family history of BC, and important etiological variables include PTEN, ATM, TP53, CHEK2, and inherent genes (BRCA1 and BRCA2, which are associated with higher risk of BC). BC risk factors include radiation, obesity, STK11, and PALB2. Consuming alcohol, smoking, high-fat and low-fibre diets, postmenopausal hormone therapy, nulliparous, single pregnancy, not breastfeeding, and the use of oral contraceptives are among the factors considered risky (Shrihastini et al., 2021). Surgery, chemotherapy, radiation (RT), endocrine therapy, targeted therapy, and immunotherapy are among the treatments for BC, and the regimens necessitate the collaboration of several subspecialties (Wang and Wu, 2023). The three most crucial anatomical features of the breasts are the lobules, ducts, and delicate tissue. The lobules are responsible for milk production, which is subsequently transported to the nipple *via* the ducts. Although there are molecular subtypes of BC based on the genetic makeup of the cancer, in actuality, the molecular subtypes depend on the degree of human epidermal growth factor receptor 2 (HER2) expression, Ki-67, progesterone receptor (PR), and estrogen receptor (ER). The resulting molecular subtypes are HER2-enriched (ER-, PR-, HER2+), triple-negative (TN) BC (ER−, PR−, HER2−), and two luminal types: Luminal A (low Ki-67, ER+ or PR+, HER2−) and Luminal B (high Ki-67, HER2+) (Zhang et al., 2023).

**FIGURE 1:**
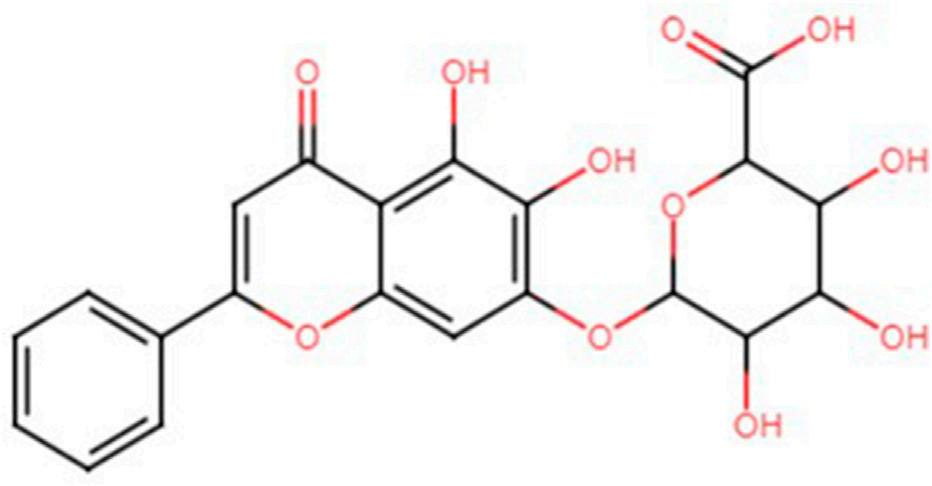
Chemical structure of BA.

Baicalin (BA) [IUPAC: 5, 6 dihydroxy-4-oxo-2-phenyl-chromen-7-yl) oxy-3, 4, 5-trihydroxy-tetrahydropyran-2-carboxylic acid] is a glycosyloxyflavone (Figure 1), which is the most abundant flavonoid component found in the *Scutellaria* genus, commonly known as ‘skullcaps,’ *Scutellaria baicalensis* (SB), and its dry roots, known as Huang-Qin or *Scutellariae Radix* (Ma et al., 2021). The organic structure revealed that BA contains two aromatic rings associated with a heteroaromatic ring and a glucuronamide.

SB is widespread in temperate regions and tropical mountains, including Europe, North America, and East Asia. This plant is a perennial herb with fleshy roots, branched stems, papery phylloclades, purplish flowers, and dark brown ovoid achenes. Since 100 BC, it has been extensively used in Japan, China, and Korea (Liang et al., 2017). BA has pharmacological properties, including anti-inflammatory, anti-apoptotic, antioxidant, antibacterial, antiviral, antidiabetic, anti-obesity, antihypertensive, anti-allergic, anti-HIV-1, anticancer, anticarcinogenic, anti-angiogenic, anti-excitotoxicity, antidepressant, and antifertility (Singh et al., 2021). BA, a principal component of Qingkailing injection, is a Chinese-patented drug commonly used in clinical settings and has received approval from the State FDA for the treatment of ischaemic stroke (Singh et al., 2021). BA has been reported to be non-toxic in animals and safe for use in humans. It has also been shown to have antitumour activities against a variety of cancers, including breast, colon, leukaemia, skin, pancreatic, prostate, and ovarian carcinoma (Huang et al., 2019).

The novelty of this study lies in the comprehensive evaluation of baicalin’s anticancer potential through advanced extraction techniques, detailed physicochemical and morphological characterisation (including scanning electron microscopy (SEM), zeta size, and zeta potential), and a wide range of *in vitro* cellular assays to assess cytotoxicity, apoptosis, mitochondrial and lysosomal integrity, and cell migration. Moreover, the integration of network pharmacology, molecular docking, and molecular dynamics simulations provides mechanistic insights into baicalin’s multi-targeted action against BC. This integrated approach highlights baicalin as a potential candidate for cancer treatment while promoting wellbeing and healthy lifestyles by bridging traditional medicine with modern biological research.

## 2 Materials and methods

### 2.1 Chemical reagents and solvents

All the chemicals used in the research were of analytical grade. Standard baicalin, aluminium chloride, and glacial acetic acid were procured from Sigma-Aldrich (United States). Ethanol, methanol, acetonitrile, HPLC-grade chemicals, and bovine serum albumin were procured from Advent Chembio Private Limited; ascorbic acid, DPPH, ABTS, quercetin, crocin, gallic acid, ferric chloride, Folin–Ciocalteu’s reagent, sodium carbonate, sodium dihydrogen phosphate, disodium hydrogen phosphate, hydrogen peroxide, potassium ferrocyanide, trichloroacetic acid, and TPTZ were procured from Himedia. MTT, Hoechst 33342, PI, LysoTracker Red DND-99, H_2_DCFDA, MitoTracker Red CMX-ROS, acridine orange, JC-1, and ethidium bromide were purchased from Invitrogen (Thermo Fisher Scientific).

### 2.2 Cell culture and maintenance

The cell lines of MCF-7 human breast cancer and L929 normal mouse fibroblast were procured from the National Centre for Cell Science (NCCS), Pune, India. Dulbecco’s modified Eagle medium (DMEM), RPMI-1640, and all cell culture reagents, including fetal bovine serum (FBS) and antibiotic–antimycotic solution, were obtained from Gibco (Thermo Fisher Scientific). The cells were grown in DMEM, which was supplemented with 10% FBS and 1% antibiotic–antimycotic solution, and incubated in a humidified incubator at 37 °C.

### 2.3 Plant material

The *Scutellaria baicalensis* dried root powder was procured from Shivay Herbals and Healthcare (SHAH/1191/2024) in Jaipur, Rajasthan, India. The company verified the details of the *S. baicalensis* dried root powder and provided a Certificate of Analysis (CoA) confirming the authenticity and quality of the material.

### 2.4 Extraction of BA

BA was prepared using three different extraction methods: probe sonication, bath sonication-assisted extraction, and maceration, following previously established procedures (Lee et al., 2014). Specifically, 5 g of dried *S. baicalensis* root powder was used. The powder was stirred in a shaker for 24 h for maceration. For bath sonication-assisted extraction, the powder was treated in an ultrasonic bath with a capacity of 2.75 L (37 kHz, 80 W) for 1 h, using a duty cycle of 25 s on and 30 s off. For probe sonication extraction, a probe sonicator (LABMAN Scientific Instruments) was used under similar conditions for 1 h with the same duty cycle. Each extraction method used a 1:20 ratio of distilled water (d/w), ethanol, and methanol as solvents. The resulting extracts were filtered using cellulose filter paper, concentrated under vacuum using a rotary evaporator (Buchi). Finally, the extracts were placed in a vacuum oven at 300 mmHg pressure and 60 °C for 8 h to eliminate residual solvents and determine the total extraction yield (Yang et al., 2013). The extraction procedure is shown in Supplementary Material S1, and the yield percentage was determined using the following formula:

$$\text{Yield }\%=\frac{\text{Amount}\;\text{of}\;\text{extract}\;}{\text{Amount}\;\text{of}\;\text{initial}\;\text{material}}\times 100.$$

### 2.5 Characterisations

#### 2.5.1 UV–VIS spectroscopy

The analysis was performed to determine the characteristic absorption peak of BA on a UV–VIS spectrophotometer (UV-1900i, Shimadzu, Japan), as described in a method with some adjustments (Zhou et al., 2024). In brief, the standard and sample were both 1 mg/mL in methanol, and their spectra were scanned in a wavelength range of 250–400 nm.

#### 2.5.2 Functional group analysis *via* FT-IR spectroscopy

Fourier-transform infrared spectroscopy (FT-IR) was carried out to evaluate the organic and inorganic contents of BA. FT-IR of BA was analysed using a Bruker ALPHA II Compact FT-IR Spectrometer (Bruker Nano GmbH, Berlin, Germany) as described earlier, with some modifications (Dmitri et al., 2023). It was analysed through 32 scans over the range of 3,500 to 500 cm^−1^ using OPUS software to determine the individual chemical groups present in the sample.

#### 2.5.3 Size and zeta potential analysis

The BA particles were characterised using the Malvern Zetasizer (v2.2), which is based on dynamic light scattering (DLS). The analysis reported particle size, polydispersity index (PDI), and zeta potential, which facilitated the measurement of particle uniformity and stability (Dalvi et al., 2022).

#### 2.5.4 HPTLC analysis

The chromatographic analysis was carried out on 10 × 10 cm glass-supported silica gel plates. BA and the standard were applied to the silica plate using a CAMAG TLC Scanner IV and a Hamilton Gastight Syringe (100 μL; 1700 Series) with a flow rate of 160 nL/s. BA standard and BA, which were formulated at concentrations of 1 mg/mL and 2 mg/mL, were used in 10 µL volumes, respectively, according to the mentioned protocol with slight modifications (Adin et al., 2022). The mobile phase (MP) was toluene/ethyl acetate/formic acid (5:4:0.2).

#### 2.5.5 HPLC analysis

The analysis was conducted using a Shimadzu HPLC instrument coupled with a PDA detector, controlled by LabSolutions software, following the previously mentioned protocol with slight variations (Khan et al., 2021). BA was separated using a C18 column (HSS, 4.6 × 250 mm, 5 µm particle size). MP consisted of methanol/water/0.1% formic acid in a ratio of 40:20:40 (v/v/v), and the MP had a flow rate of 1,000 μL/min. The temperature of the column was maintained at 40 °C for efficient separation. The two samples received a 10 µL volume each. Detection was conducted using a PDA detector, and BA was detected at its wavelength. BA was dissolved in methanol to prepare the samples at the desired concentration (Yang et al., 2013). The retention time for BA was determined by running a standard of BA under the same conditions.

#### 2.5.6 Liquid chromatography–mass spectrometry examination

The study was conducted using an LC-QTOF (Agilent) integrated with a electro spray ionization. The MP consisted of water and acetonitrile in a ratio of 85:15 (v/v) as per the previously described protocol, with slight modifications (Chung et al., 2012). The separation was conducted using a reversed-phase C18 column with a gradient flow system at a flow rate of 300 μL/min. To ensure effective separation, the column temperature was set at 35 °C ± 2 °C. A 1 µL capacity was used for the analysis. The gradient program was initiated with 15% eluent B, progressively increasing to 65% over 3 min, followed by an increase to 85% within 1 min, which was maintained for 2 min; the total run duration was 12 min.

#### 2.5.7 XRD analysis

An analysis was performed to assess whether the particles were crystalline or amorphous. Diffraction intensities were recorded from 10° to 90° 2θ as per the previously described procedure with minor modifications (Li et al., 2018).

#### 2.5.8 NMR analysis

^13^C and ^1^H-NMR analyses were assessed on an AVANCE III 500 NMR Spectrometer (BRUKER Corporation, Billerica, MA, United States). The sample preparation involves dissolving approximately 10 mg of BA for ^1^H NMR and 20–30 mg for ^13^C NMR in a suitable deuterated solvent, such as DMSO-D_6_, as per the previously described procedure with minor modifications (Zhang et al., 2016). For ^1^H NMR, a sweep width of 16 ppm was set, and 16–64 scans were typically performed with a pulse width of 8–12 μs and a relaxation delay of 1–2 s. For ^13^C NMR, the sweep width was set to 220 ppm, with 1,000–5,000 scans, a pulse width of 10–15 μs, and a relaxation delay of 1–5 s.

#### 2.5.9 Morphological analysis by scanning electron microscopy

The particle’s surface morphology, size, and shape were observed using a scanning electron microscope (Apreo LoVac, FEI, Inc., Hillsboro, OR, United States). BA was placed on an aluminium mount with a self-adhesive carbon layer and coated with a fine layer of gold using an ion sputter in an inert gas environment (Li et al., 2018).

### 2.6 Phytochemical screening: quantitative

#### 2.6.1 Estimation of total polyphenolic content

The total polyphenolic content (TPC) was assessed according to the previously defined protocol (Baba and Malik, 2015), with slight modifications. A 1 mg/mL solution of BA (100 µL) was combined with 500 µL of 10% v/v FC chemical, thoroughly mixed, and kept for 2 min at 37 °C. Then, 0.4 mL of 7.5% Na_2_CO_3_ was mixed, and the reaction continued for 15 min at 50 °C. After cooling to room temperature, a blue colour developed, and the OD was measured at 760 nm using a multi-mode microplate reader (Synergy H1, BioTek, United States). The gallic acid was used as a standard (20–100 μg/mL), and the findings were represented in terms of gallic acid equivalents (GAE) in mg/g as per the following formula:

$$\text{TPC }\left(\text{mg}/g\text{ GAE}\right)=C*\;V/M,$$

where “C” is the concentration of gallic acid, “V” is the volume of extract in mL, and “M” is the dry weight (g) of the raw material.

#### 2.6.2 Total flavonoid content

Total flavonoid content (TFC) of BA was determined according to the established protocol (Lim et al., 2024), with slight modifications. In brief, 1,000 µL of a 2% w/v aluminium trichloride in MeOH was added to 1,000 µL of BA and left at room temperature for 1 h. Upon incubation, a yellow colour was formed, and O.D. was measured at 510 nm using a multi-mode microplate reader (Synergy H1, BioTek, United States). The TFC was quantified in relation to a standard curve of quercetin (20–100 μg/mL), according to the following formula:

$$\text{TFC }\left(\text{mg}/g\text{ QE}\right)=C*\;V/M,$$

where “C” is the concentration of quercetin, “V” is the volume of BA, and “M” is the dry weight (g) of the raw material.

#### 2.6.3 Total flavonol content

Quercetin was used as a standard to evaluate the total flavonol content (TFolC) in BA, following a slightly modified version of a previously described protocol (Al-Mustafa et al, 2021). In brief, 0.5 mL of BA or the standard at different dilutions was combined with 1.5 mL of sodium acetate (50 mg/mL) and 0.5 mL of AlCl_3_ (20 mg/mL). After incubating the reaction mixture for 2.5 h, its absorbance was measured at 440 nm using a multimode microplate reader (Synergy H1, BioTek, United States).

#### 2.6.4 Total protein content

BSA was used as a standard to create a standard curve for estimating protein concentrations ranging from 20 to 100 μg/mL. To prepare the reagents, 4.5 mL of reagent A (which consists of 0.048 L of 2% Na_2_CO_3_ in 0.1 N NaOH, 1,000 µL of 1% KNaC_4_H_4_O_6_·4H_2_O, and 1 mL of 0.5% CuSO_4_) was mixed with BA and incubated for a quarter of an hour as per the defined protocol (Sarkar et al., 2020) with minor adjustments. Afterwards, 500 µL of freshly prepared reagent B (a 1:1 mixture of Folin–Ciocalteu’s reagent and water) was mixed with the sample, and the reaction was kept in the dark for half an hour. Absorbance was measured at 660 nm.

### 2.7 Anti-inflammatory activity

The *in vitro* anti-inflammatory activity (AIA) of BA was analysed using the stabilisation of the albumin structure technique. Cromol (crocin) and quercetin were used as standard drugs. BA was solubilised in DMSO and subsequently diluted using a 0.2 M phosphate-buffered saline (PBS) at pH 7.4. BA was mixed with different concentrations (0.6–3 mL) of a 1 mM albumin solution prepared in PBS and maintained at 27 °C ± 1 °C for 15 min. Denaturation was triggered by maintaining the reaction at 59 °C ± 2 °C for 10 min, as per the defined protocol (Gondkar et al., 2013), with minor adjustments. After cooling, absorbance was measured at 660 nm using a multimode microplate reader (Synergy H1, BioTek, United States). The percentage inhibition of denaturation was calculated.

$$\%\text{ Inhibition}=100\times \left(\left(\frac{\text{Vc}}{\text{Vt}}\right)-1\right),$$

where Vt and Vc are the mean optical density values of the test and control groups, respectively.

### 2.8 Antioxidant activities

#### 2.8.1 Determination of total antioxidant activity: DPPH assay

The antioxidant capacity of BA was evaluated by its ability to scavenge the DPPH free radical according to a previously described protocol (Durmaz et al., 2023), with minor adjustments. In brief, the stock solution of the extract was suspended in deionised water, and a range of concentrations (20–100 μg/mL) was tested. A 3,000 µL DPPH (0.1 mM) solution was added in methanol with several dilutions of BA. Ascorbic acid (AA) served as the standard positive control, while a DPPH-methanol solution without the extract acted as the negative control. Only methanol was used as the blank. The suspension was kept in the dim light at ambient temperature for 30–35 min, and O.D. was measured at 517 nm using a multimode microplate reader (Synergy H1, BioTek, United States). The free radical scavenging activity (FRSA) percentage was determined using the following formula:

$$\%\text{FRSA}=\left(\text{Abs}.\text{ of Control}-\text{Abs}.\text{ of Sample}\right)/\left(\text{Abs}.\text{ of Control}\right)\times 100$$

#### 2.8.2 FRAP assay

The FRAP assay was performed as per the previous protocol (Durmaz et al., 2023) with minor modifications. BA was prepared in various concentrations, and AA was used as a standard, supplemented with 3000 µL of the FRAP reagent. The reagent consisted of 300 mM sodium ethanoate buffer (pH 3.6), 10 mM TPTZ, and 20 mM ferric trichloride, mixed together in a 10: 1: 1 ratio, respectively. The reaction was maintained at room temperature for 30–35 min, and O.D. was measured at 593 nm using a multimode microplate reader (Synergy H1, BioTek, United States).

#### 2.8.3 ABTS assay

The ABTS assay was performed according to a previously described protocol (Durmaz et al., 2023), with minor modifications. The ABTS^+^ solution was prepared by mixing an equal volume of 2.4 mM K_2_S_2_O_8_ and 7 mM ABTS solution and then placing the reaction in the dark at room temperature for 24 h. The ABTS^+^ solution was then diluted with distilled water (d/w) to achieve an optical density of 0.7 ± 0.01 at 650 nm. Various dilutions of BA (20 μL each) were mixed with 80 μL of the ABTS^+^ solution in a microplate and incubated for 4 min at room temperature. O.D. was determined at 650 nm using a multi-mode microplate reader (Synergy H1, BioTek, United States). AA was used as a standard, deionised water served as the blank, and the ABTS^+^ solution acted as the control. ABTS/RSA was calculated using the specified formula:

$$\text{ABTS/RSA}\left(\%\right)=\left[1-\left(\text{Abs}.\text{Sample}-\text{Abs}.\text{Blank}\right)/\left(\text{Abs}.\text{Control }\right)\right]\times 100$$

#### 2.8.4 H_2_O_2_ scavenging activity

The ability of BA to reduce H_2_O_2_ and exhibit antioxidant potential was investigated using a slightly modified protocol (Peng-Fei et al., 2013). In brief, BA was prepared in various concentrations and suspended in PBS. A 43 mM H_2_O_2_ solution was prepared in 0.1 M PBS (pH 7.4). Due to light sensitivity, the experiment was performed in dark conditions. Different aliquots of the extract were placed in amber-coloured Falcon tubes, and 3.4 mL of PBS was added to each tube, followed by 0.6 mL of the H_2_O_2_ solution. The mixtures were thoroughly mixed using a vortex shaker at 500 rpm for 15–20 s (Thermo Scientific, United States). Subsequently, the sample was kept at ambient temperature for a quarter of an hour, and O.D. was measured at 230 nm using a UV–visible spectrophotometer (UV 1900i, Shimadzu, Japan). AA was used as the standard, PBS was used as the blank, and H_2_O_2_ was used as the control. The % of H_2_O_2_ scavenging activity was determined using the specified formula:

$$\%\text{ Scavenged }\left[H_{2}O_{2}\right]=\left(O.D.\text{ of Control}-O.D.\text{ of Sample}\right)/\left(O.D.\text{ of Control}\right)\times 100$$

#### 2.8.5 RPA assay

BA and its ability to convert ferric ions (Fe^3+^) to ferrous ions (Fe^2+^) were assessed using a protocol defined by Peng-Fei et al. (2013). In brief, aliquots of BA, prepared in deionised water, were combined with 2,500 µL of 0.2 M PBS (pH 6.6) and 2,500 µL of 1% [K_4_Fe (CN)_6_]. The reaction mixture was thoroughly mixed, covered with aluminium foil, and maintained at 50 °C for half an hour in a water bath. Following incubation, 2,500 µL of 10% TCA was added to each tube, and the reaction was spun down at 3,000 rpm for 5–6 min at 4 °C using a Thermo Scientific Sorvall ST8R Centrifuge. The upper layer was then supplemented with 2,500 µL of deionised water, and 500 µL of 0.1% (w/v) ferric chloride was added, forming a bluish-coloured solution. AA was used as a standard, and O.D. was measured at 700 nm using a multimode microplate reader (Synergy H1, BioTek, United States).

### 2.9 Determination of haemolytic inhibition

A haemolysis assay or a blood compatibility assay was performed to ascertain the possible side effects of BA on blood. The blood compatibility of BA with human red blood cells (RBCs) was analysed under a protocol explained by Wei et al. (2017) and Liu et al. (2023), with minor modifications. Blood was collected under aseptic conditions from a healthy multidonor, with consent from Parul Sevashram Hospital, Parul University. The blood, contained in an EDTA vial, was spun down at 1.5 k rpm for a quarter of an hour at 4 °C to separate RBCs from the serum, which was then removed. The RBC pellet was washed three times with sterile 0.9% NaCl solution, and finally, an equivalent volume of NaCl solution was mixed. BA was prepared in various concentrations. A 300 μL RBC suspension was mixed with each BA concentration. Positive control (PC) and negative control (NC) were prepared by mixing 300 μL of RBC suspension with 1 mL of d/w and 0.9% saline solution, respectively. After a 2 h incubation, the samples were spun down at 5,000 rpm for 5 min at 4 °C. The O.D. of the supernatant was measured at 540 nm using a multimode microplate reader (Synergy H1, BioTek, United States), which correlates with the amount of haemoglobin released. The % of haemolysis was calculated using the following equation:

$$\%\text{ Hemolysis}=\left(\frac{O.D.\;\text{of}\;\text{Sample}-O.D.\;\text{of}\;\text{NC}}{O.D.\;\text{of}\;\text{PC}-O.D.\;\text{of}\;\text{NC}}\right)\times 100$$

### 2.10 Isolation of peripheral blood mononuclear cells from blood

Following plasma separation, the buffy coat underwent dextran sedimentation (1% w/v) at 37 °C for 30 min. The upper leucocyte-rich suspension was transferred to a new tube and centrifuged for 5 min at 500 g (Kumar et al., 2018). The pellet was resuspended in 2 mL of Hank’s balanced salt solution (HBSS) and loaded above 2 mL of Histopaque-1077 Hybri-Max, with a density of 1.077 g/cm^3^, and then centrifuged at 700 *g* for 15 min (Kumar et al., 2021). The interface layer (1.080–1.065) was retrieved and washed twice with HBSS by centrifugation at 200 *g* for 10 min at 37 °C and then resuspended in RPMI-1640 medium.

### 2.11 *In silico* studies through bioinformatics tools

#### 2.11.1 Information collection on BA

The PubChem database (https://pubchem.ncbi.nlm.nih.gov/) provides essential information, such as common names, PubChem CID, canonical SMILES, and other details, to identify components, as per the previously mentioned protocol (Yuan et al., 2021). To analyse the ADMET parameters of the components, the SwissADME (http://www.swissadme.ch/) (Daina et al., 2017) and pkCSM (http://biosig.unimelb.edu.au/pkcsm/predic3tion) (Pi et al., 2015) databases were used.

#### 2.11.2 Prediction of potential targets for BA

The SwissTargetPrediction database (https://labworm.com/tool/swisstargetprediction) (Gfeller et al., 2014) was built upon a repository of 370,000 identical compounds across more than 3,000 proteins. The 3D structure of BA was imported to identify potential targets.

#### 2.11.3 Selection of potential targets

The GeneCards database (https://www.genecards.org/) (Stelzer et al., 2016) offers detailed data on all labelled and predicted individual genes from 150 digital sources. The databases were used to identify genes related to breast cancer. The Venny 2.1.0 database (https://bioinfogp.cnb.csic.es/tools/venny/) (Nagam, 2020) was used to screen for selected targets associated with both BA and the disease (Yuan et al., 2021).

#### 2.11.4 Protein interaction mapping

The STRING database (https://string-db.org/) (Szklarczyk et al., 2023) contains over fifty-two million macromolecules from more than 1,100 species, primarily used to establish protein–protein interactions (PPIs). To analyse the 25 selected targets, they were entered into STRING. The PPI network was retrieved and loaded into Cytoscape 3.7.1 (https://cytoscape.org/download.html) (Shannon et al., 2003). Topological analysis was performed using the node degree value exceeding the median as the criterion for screening significant targets (Yuan et al., 2021).

#### 2.11.5 Binding site prediction

Predicting binding sites is essential in drug discovery and development. Various *in silico* tools, including PUResNet (Kandel et al., 2021), CASTp (Tian et al., 2018), and BSpred (Mukherjee and Zhang, 2011), employ surface cavity detection for this purpose. In this study, CASTp was used to identify potential binding sites.

#### 2.11.6 Molecular docking

AutoDockTools-1.5.7 (Morris et al., 2009) (Trott and Olson, 2010) was software for molecular docking. BIOVIA Discovery Studio (https://discover.3ds.com/discovery-studio-visualizer-download) is used (Khamto et al., 2021) for visualising ligands and proteins. RSCB PDB (https://www.rcsb.org/) is a database that contains 10,000 gene targets (Xiang et al., 2021) and analyses the active site of the protein CASTp. To improve the reliability of the molecular docking results, proteins were obtained from the PPI network and downloaded in PDB format. The 3D structures of the components were sourced from the PubChem database (https://pubchem.ncbi.nlm.nih.gov/). These structures were then processed in AutoDockTools-1.5.7, where they were hydrogenated and charged, and their numbers of rotatable bonds were calculated. The lowest energy pose was selected for analysis using the clustering tool in AutoDockTools-1.5.7. High-resolution 3D and 2D representations of small molecules and macromolecules were generated and examined using the BIOVIA Discovery Studio visualisation tool, highlighting the associated protein residues and binding interactions. It is commonly accepted that a lower docking binding free energy, specifically below −4 kcal/mol, indicates a stronger binding capacity (Yuan et al., 2021).

#### 2.11.7 Molecular dynamics simulation

The protein–ligand complex was analysed further through a 500 ns molecular dynamics simulation (MDS) to mimic stability, flexibility, and compactness in the real system. The MDS was performed using GROMACS version 2024.1 with the CHARMM36 force field and TIP3P water model (Sousa da Silva and Vranken, 2012). The complex was added in a cubic solvation box, and ions were further added to neutralise and equilibrate the system. The energy minimisation was carried out with a maximum force set to lower than 1,000 kJ/mol, and the system was then subjected to a two-phase equilibration. The initial phase was NVT equilibration at 300 K, controlled by a V-rescale thermostat, followed by NPT equilibration (Van Der Spoel et al., 2005). Finally, 500 ns MDSs were performed to evaluate the dynamic behaviour and stability of the protein and protein–ligand complexes (Wicaksono et al., 2024). The MDS parameters, including root-mean square deviation (RMSD), root-mean square fluctuation (RMSF), the radius of gyration (Rg), solvent accessible surface area (SASA), and intramolecular hydrogen bonding, were evaluated.

### 2.12 Assessment of anticancer activity

#### 2.12.1 Cell viability assay

Cell viability (CV) of BA was compared to MCF-7 BC cells, L929 healthy fibroblast cells, and isolated peripheral blood mononuclear cells (PBMCs) for CV using the cytotoxicity assay with minor modifications (Ignatova et al., 2011). In brief, 10,000 cells/well were plated in a 96-well plate and cultured overnight. Following incubation, the medium was discarded, and cells were exposed to different aliquots of BA. The + ve control was 5-fluorouracil (5-FU), and the − ve control was untreated cells (Vyas et al., 2025). Treatments were incubated for an additional 24 h under the same conditions. After treatment, media were gently aspirated, and 10 μL of the MTT solution (5 mg/mL in PBS) was added to each well. The plate was kept at 37 °C for 4 h to allow viable cells to convert the MTT into purple formazan crystals. One hundred microlitres of DMSO were added to each well in order to dissolve the crystals. OD was measured at 545 nm using a microplate reader (Synergy H1, BioTek, United States). The % of cell viability was calculated using the following formula:

$$\%\text{ Viability}=\left(\frac{O.D.\;\text{of}\;\text{the}\;\text{treatment}\;}{O.D.\;\text{of}\;\text{Control}}\right)\times 100$$

#### 2.12.2 Neutral red cellular uptake assay

To further analyse the cytotoxic activity of BA, the neutral red uptake (NRU) assay was conducted according to a previously mentioned protocol (Siddiqui et al., 2015), with minor modifications. MCF-7 cells (10000 cells per well) were seeded in 96-well plates and incubated for 24 h. Once the cells settled and attached, they were exposed to differing concentrations of BA and incubated overnight (Kumar et al., 2025). After treatment, the medium was changed to culture medium with neutral red dye (50 μg/mL), and cells were incubated for an additional 3 h to allow dye incorporation. After incubation, the wells were rinsed gently with a solution of 0.5% formaldehyde and 1% calcium chloride to remove unincorporated dye. Next, a 1% acetic acid and 50% ethanol destaining solution was added to every well to remove the absorbed dye from cells. O.D. at 545 nm was recorded using a microplate reader to assess CV (Siddiqui et al., 2020).

#### 2.12.3 Morphological observations

Morphological alterations in cells after BA treatment were measured with a previously developed method (Liu et al., 2012), with minor adjustments. In brief, 50,000 cells/well were plated in a 24-well plate and incubated overnight. After that, cells were treated with various concentrations of BA and incubated for an additional 24 h under the same conditions (Yadav et al., 2025). Following the treatment duration, the cells were gently exposed to PBS to remove any remaining media or compound. Morphological alterations were then observed using an EVOS FLoid Cell Imaging Station fluorescence microscope for visual comparison between treated and control cells.

#### 2.12.4 Reactive oxygen species generation

ROS production after BA treatment was calculated following a previously described protocol (Yadav et al., 2020), with slight modifications. In brief, 5 × 10^4^ cells per well were seeded in a 24-well plate and incubated overnight. The cells were treated with varying aliquots of BA and incubated for an additional 24 h under the same conditions. Following treatment, the cells were washed with PBS under gentle conditions and stained with 20 µM DCFDA dye. The plate was held for 20 min in the dark. Subsequently, images were recorded using the EVOS FLoid Imaging Station.

#### 2.12.5 Determination of nuclear morphology and DNA fragmentation with Hoechst 33342 staining

Hoechst 33342 is a cell-permeant fluorescent dye that selectively stains nuclei by binding to the minor groove of DNA and with a preference for adenine–thymine-rich sequences (Atale et al., 2014). Nuclear staining with Hoechst 33342 was performed following a previously mentioned protocol (Ansari et al., 2016), with slight modifications. Cells were seeded at 5 × 10^4^ cells per well in a 24-well plate and incubated overnight. After 24 h, cells were treated with different aliquots of BA and incubated for an additional 24 h. After treatment, the medium was removed, and 10 μL of Hoechst 33342 working solution (1 μg/mL in PBS) was added to each well (Verma et al., 2024). The cells were then maintained at 37 °C for 5 min in the dark. Nuclear changes were then imaged and visualised upon incubation using the EVOS FLoid Cell Imaging Station.

#### 2.12.6 Assessment of apoptosis using propidium iodide staining

Apoptotic alterations in BA-treated cells were observed by propidium iodide (PI) staining, following a method previously mentioned (Kamble et al., 2022), with some modifications. A total of 5 × 10^4^ cells per well were seeded into a 24-well plate and incubated. After 24 h, the cells were treated with various BA aliquots and incubated for 24 h. Cells were then gently washed after treatment and stained with 1 μg/mL PI (prepared from a 1 mg/mL stock solution) at the final concentration. The plate was kept in the dark at 37 °C for 10 min to enable dye binding to the DNA of membrane-damaged (apoptotic or necrotic) cells. Following staining, cells were instantaneously visualised and imaged using the EVOS FLoid Imaging Station to determine apoptosis.

#### 2.12.7 Assessment of lysosomal activity using LysoTracker Red DND-99

Acidic organelles, particularly lysosomes, were imaged using LysoTracker Red (100 nM) according to a previously reported protocol (Guerra et al., 2019), with minor adjustments. In brief, 5 × 10^4^ cells per well were plated in a 24-well plate and kept overnight. Following cell attachment, various aliquots of BA were applied, and the cells were cultured for an additional 24 h under the same conditions. After treatment, the cells were stained with LysoTracker Red (100 nM) and kept in the dark for 30 min. Cells were immediately imaged after staining using the EVOS FLoid Imaging Station to evaluate changes in lysosomal integrity and fluorescence intensity.

#### 2.12.8 Dual staining: MitoTracker Red CMX-ROS and Hoechst 33342

Simultaneous staining with MitoTracker Red CMX-ROS and Hoechst 33342 was carried out to observe mitochondrial integrity and nuclear morphology (Chen et al., 2020). Approximately 5 × 10^4^ cells per well were plated in a 24-well plate and kept for 24 h. After that, cells were treated with various aliquots of BA and kept overnight. Cells were stained with 10 μL of MitoTracker Red and kept for 20 minutes at 37 °C after treatment to mark active mitochondria. Then, the MitoTracker dye was washed off, and 10 µL of Hoechst 33342 working solution was pipetted into each well. The cells were kept for another 15 minutes at 37 °C to stain the nuclei. Following staining, cells were washed carefully and imaged using the EVOS FLoid Imaging Station to observe mitochondrial and nuclear dynamics in real-time.

#### 2.12.9 Assessment of mitochondrial membrane potential through JC-1

Mitochondrial membrane potential (MMP) was assessed according to a previously described protocol (Khanam et al., 2018), with minor adjustments. In brief, 5 × 10^4^ cells per well were plated in a 24-well plate and incubated overnight. Cells were treated with various aliquots of BA and kept for another 24 h. Upon treatment, the cells were incubated with 300 nM JC-1 dye for 30 min. Post-staining, MMP alterations were observed using the EVOS FLoid Imaging Microscope.

#### 2.12.10 Dual staining with AO/EtBr for early and late apoptosis detection

Dual staining was carried out to identify apoptosis, as per a previously reported protocol (Palanivel et al., 2020), with some modifications. Approximately 5 × 10^4^ cells/well were seeded into a 24-well plate and maintained for 24 h. Cells were treated with different aliquots of BA and maintained for 24 h. Cells were stained with 5 μL of acridine orange and ethidium bromide after treatment, both of which had been prepared at a concentration of 5 mg/mL. The stained cells were immediately visualised using the EVOS FLoid Imaging Station to identify morphological changes corresponding to early and late apoptosis.

#### 2.12.11 Cell migration/scratch assay

A scratch assay was performed to evaluate the impact of BA on cell migration, using a previously reported protocol (Chen and Nalbantoglu, 2014) with minor adjustments. Approximately 5 × 10^4^ cells/well were plated into a 6-well plate and allowed to fully confluence. Upon reaching confluency, a gentle scratch was created across the cell monolayer using a 200 µL sterile pipette tip to mimic a wound. The cells were imaged on the EVOS FLoid Imaging Station to record the primary wound area after scratching. The cells were then treated with the IC_50_ value of BA and kept overnight. The % of wound area was calculated using the following formula.

$$\text{Wound area }\%=\frac{\text{At}}{A0}\times 100,$$

where At is the wound area after treatment and A0 is the wound area before treatment.

### 2.13 Statistical analysis

Data collection and summarisation were initially carried out using a Microsoft Excel Worksheet; each experiment was repeated at least three times (n = 3) in triplicate. All experimental data were presented as the mean ± SD. A one-way analysis of variance was used to compare the groups. Statistical analysis was performed using GraphPad Prism 8.0 and Origin Pro 2024 (Origin Lab Corporation, Hampton, MA, United States). *p* < 0.05 was considered to indicate a statistically significant difference. All images were captured at a resolution corresponding to a scale bar of 100 µm.

## 3 Results

### 3.1 BA extraction preparation

The extract of BA was prepared using maceration, bath sonication, and probe sonication. Initially, 5 g of SB root powder were taken, and d/w, ethanol, and methanol were used as solvents, resulting in different yield percentages for the various solvents and ratios. Supplementary Table S1 provides the details of the amount of BA produced and the yield percentage.

### 3.2 UV–VIS spectroscopy

**FIGURE 2:**
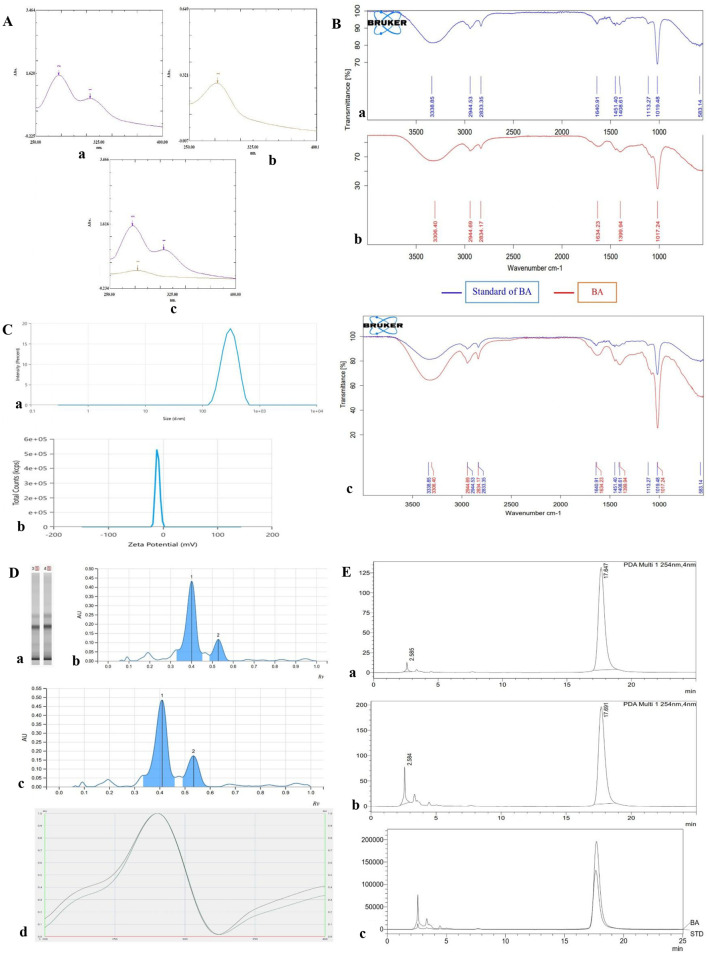
**(A)** UV–VIS spectrum. (a) Standard BA; (b) BA; (c) overlay spectrum of the standard BA and extracted BA. **(B)** FT-IR spectrum of BA. (a) Spectrum of standard BA; (b) spectrum of BA; (c) overlay spectrum of standard BA and BA to observe the shifting (wavenumber) of the functional group. **(C)** Size analysis showing the average diameter of BA. (a) The typical size of BA is 385.4 nm, and its PDI value is 0.4; (b) zeta potential of BA is −9.302. **(D)** HPTLC spectra. (a) Silica plate after separation (b); standard BA (c); BA (d); overlay spectrum of standard BA and BA was observed at retention factor (RF) values of 0.328 and 0.333, confirming the presence of BA. **(E)** HPLC graphs: (a) standard BA; (b) BA; (c) overlay graphs of standard BA and BA were observed at retention times (Rts) of 17.647 and 17.691, confirming the presence of BA.

The characteristic peak of BA, a key phytoconstituent of BA, exhibits a maximum absorption within a range of 277–283 nm, reaching a maximum absorption value of 1.5, as illustrated in Figure 2A.

### 3.3 Functional group analysis by FT-IR

The distinctive functional groups in BA were identified using FT-IR. The sample was scanned from 3,500 to 1,000 cm^-1^. Qualitative phytochemical functional group analysis of standard BA and BA, with FT-IR wave number (cm^−1^), is mentioned in Supplementary Table S2. In contrast, individual FT-IR spectra of standard BA and extracted BA and merged spectra are represented in Figure 2B.

### 3.4 Zeta potential and size distribution measurement

Particle size and PDI of the BA formulation were determined using the Zetasizer. As evident from Figure 2C, BA was found to have an average size of 385.4 nm. The PDI was 0.4, which is less than 1, meaning that the particle distribution was relatively uniform and homogeneous.

### 3.5 HPTLC analysis

High-performance thin-layer chromatography (HPTLC) analysis was carried out to compare the standard BA with BA. Following development, the silica gel plate was observed at 286 nm, which is the λ_max_ of BA. The comparison revealed the presence of BA in the extract, as indicated by the similar Rf values and UV absorption characteristics at 286 nm. The developed HPTLC plates displayed distinct BA bands, confirming the compound’s successful extraction and identification, as shown in Figure 2D.

### 3.6 HPLC analysis

HPLC analysis was performed to compare the standard BA with BA. The characteristic peak of the BA standard was observed at a retention time of approximately 17.6 min. BA was then analysed, and its chromatogram was compared with the BA standard. The extract showed a peak at the same retention time as the BA standard, confirming the presence of BA in the extract shown in Figure 2E.

### 3.7 LC-MS analysis

**FIGURE 3:**
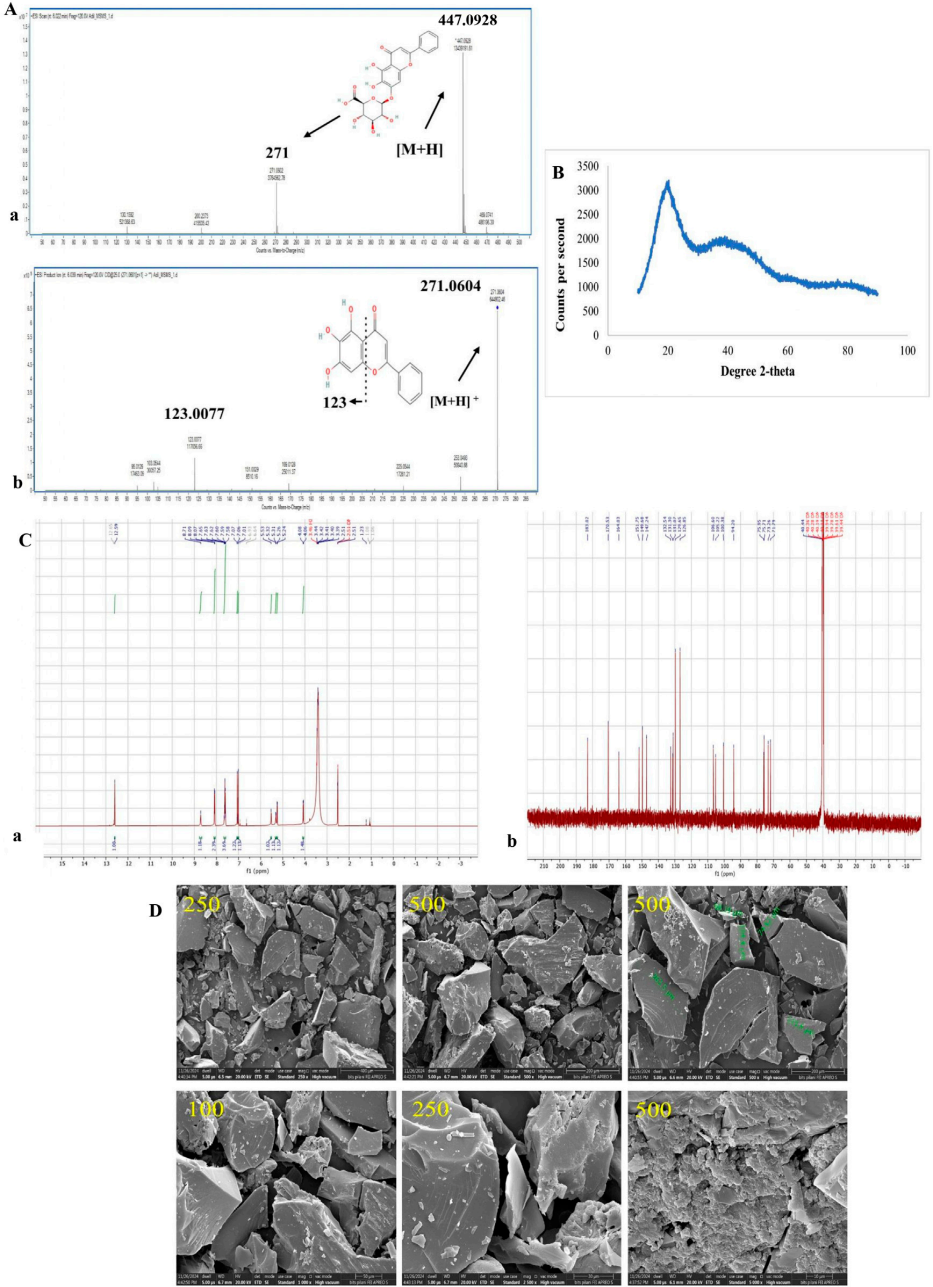
**(A)** Mass spectra of fragmented ions of (a) baicalin and (b) baicalein as obtained from LC–MS/MS. (a) The MS/MS fragmentation pattern of baicalin has a molecular ion [M–H]-at m/z 447, which corresponds to its molecular weight. The major fragment ions are m/z 271, representing the loss of a glucuronide moiety, which establishes the presence of a flavone backbone. (b) Fragmentation of baicalein, with a [M–H]^−^ molecular ion at m/z 271, and additional fragment ions at m/z 123 corresponding to typical cleavages of the flavone skeleton. These spectra confirm the structural verification and molecular identity of baicalin and its aglycone baicalein. **(B)** XRD graph of BA. The diffractogram reveals a broad halo with the absence of sharp crystalline-like peaks, indicating the amorphous nature of BA. **(C)** NMR spectrum analysis of BA: (a) ^1^H NMR spectrum and (b) ^13^C NMR spectrum. (a) ^1^H NMR spectrum, with typical proton signals for aromatic protons, hydroxyl groups, and the sugar moiety of BA. (b) ^13^C NMR spectrum, showing the carbon chemical shifts for both the flavone backbone and the glucuronide sugar unit. The chemical shifts obtained ensure the structural identity and purity of BA. (D) Morphological representation by scanning electron microscopy, images of baicalin at different magnifications of 400 µm, 200 µm, 30 µm, and 10 µm.

The analysis was carried out in +ve ion mode, where the electrospray interface of BA and baicalein (BE) resulted in [M + H]^+^ ions at m/z 447.0928 and 271.0604, respectively. Fragmentation spectra were acquired for each charged molecule. The most significant fragment ions identified in the spectrographs were used to establish the multiple reaction monitoring (MRM) shifts: m/z 271.0604→123.0077 for baicalein and m/z 447.0928→271.0604 for baicalin, as shown in Figure 3A.

### 3.8 XRD analysis

XRD analysis revealed that BA exhibited a broad diffraction peak between 2θ values of 6° and 18°, consistent with its amorphous nature, as shown in Figure 3B. The non-appearance of sharp peaks confirms its non-crystalline structure. This amorphous form is most commonly linked to better solubility and dissolution characteristics than its crystalline analogue, which can be useful in increasing its bioavailability.

### 3.9 NMR analysis

In the ^1^H NMR spectrum of BA, different peaks were observed at 6–8 ppm, corresponding to aromatic protons, and a singlet at 12.5 ppm related to a hydroxyl proton involved in intramolecular hydrogen bonding. The anomeric proton of the glucuronic acid moiety was identified at 4.5–5.5 ppm. Additional sugar protons resonated in the 3–4.5 ppm range. In the ^13^C NMR spectrum, the carbonyl carbon of the flavone structure was detected at 182–184 ppm, with aromatic carbons appearing between 94.5 and 161.2 ppm. The anomeric carbon of the sugar unit resonated at ∼100 ppm, while other sugar carbons were observed between 60 and 80 ppm, as shown in Figure 3C.

### 3.10 SEM of BA

The analysis of BA was conducted to investigate 400 µm, 200 µm, 50 µm, 30 µm, and 10 µm. The shape of BA was irregular, and the size was 68.19–263.5 µm, as shown in Figure 3D.

### 3.11 Total phenolic content

**FIGURE 4:**
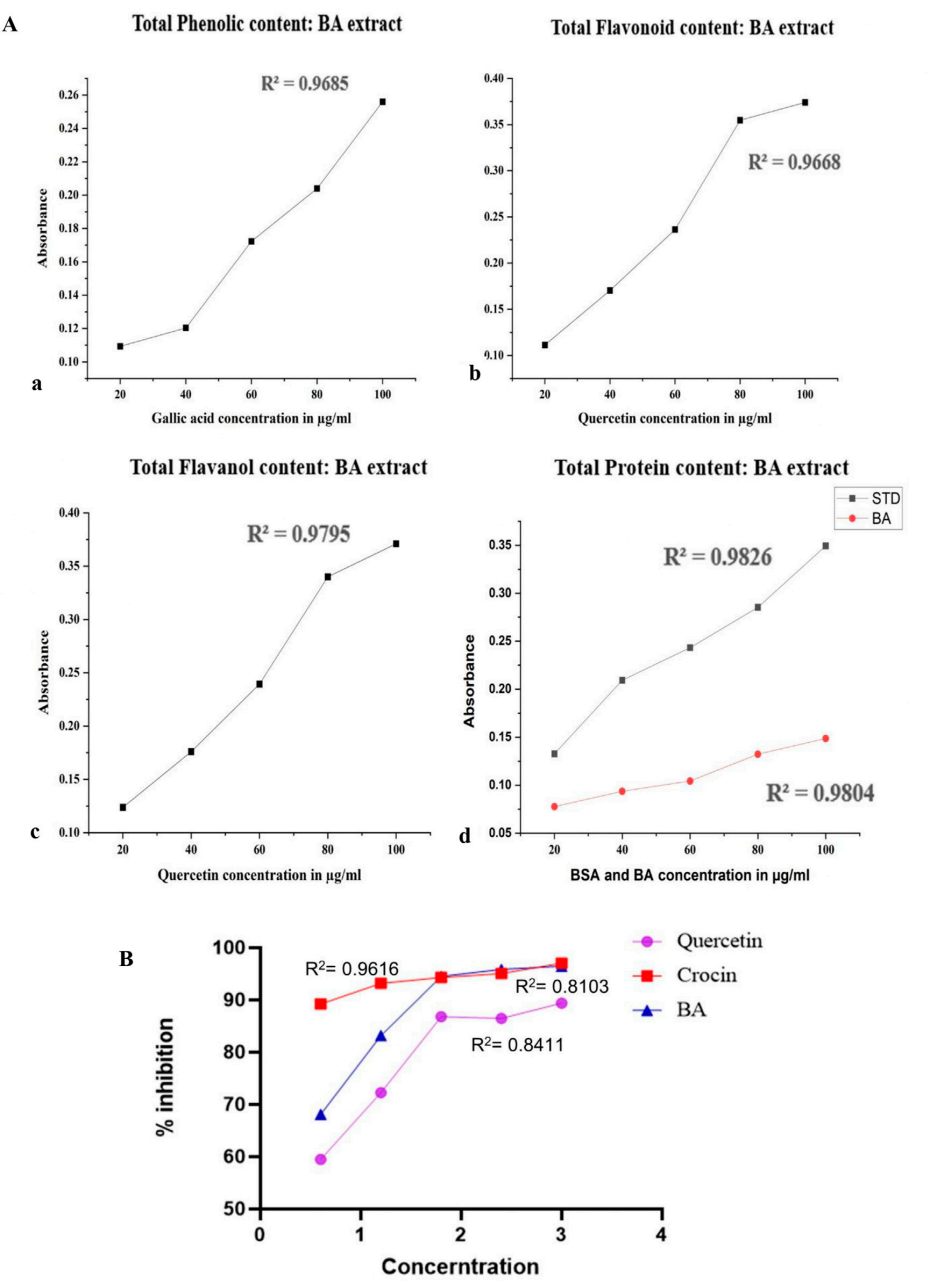
**(A)** Phytochemical screening. (a) TPC in BA using standard gallic acid at various concentrations (20–100 μg/mL). All the values were presented as the mean ± S.D. (b) Total flavonoid content in BA was extrapolated using standard quercetin at various concentrations (20–100 μg/mL). All the values were presented as the mean ± S.D. (c) Total flavonol content in BA was extrapolated using standard quercetin at various concentrations (20–100 μg/mL). All the values presented as the mean ± S.D. were determined in triplicate. (d)The total protein content in BA was extrapolated using standard BSA at various concentrations (20–100 μg/mL). All the values presented as the mean ± S.D. were determined in triplicate. **(B)** % Inhibition of albumin denaturation. All the values presented as the mean ± S.D. were determined in triplicate.

The TPC of BA, shown as GAE, was measured using the Folin–Ciocalteu method. The TPC in BA was assessed to be 99.9 ± 0.27 mg/g GAE dw, based on the gallic acid calibration curve depicted in Figure 4A (a).

### 3.12 Total flavonoid content

The TFC in BA was assessed using a quercetin equivalent standard calibration curve. The quercetin standard curve showed that BA contained 80.4 ± 2 mg QE per Gram of TFC, as shown in Figure 4 (b).

### 3.13 Total flavonol content

The TFolC in the BA was assessed using the analytical standard curve of quercetin, as illustrated in Figure 4(c). The extract’s total flavonol content was 79.4 ± 2 mg/g QE.

### 3.14 Total protein content

The total protein content in BA was extrapolated using the standard calibration curve of BSA mentioned in Figure 4D. The total protein content results revealed that the redox activity of BA was significantly enhanced in a dosage-dependent manner.

### 3.15 Anti-inflammatory activity

The AIA of BA, crocin, and quercetin was assessed, and it showed that BA has a similar anti-inflammatory profile to crocin, while it has greater anti-inflammatory activity than quercetin. The results exhibited a significant increase in AIA with % inhibition of albumin denaturation at 68%, 83%, 94%, 95%, and 96%, respectively, in a dose-dependent manner, as shown in Figure 4B.

### 3.16 DPPH assay

**FIGURE 5:**
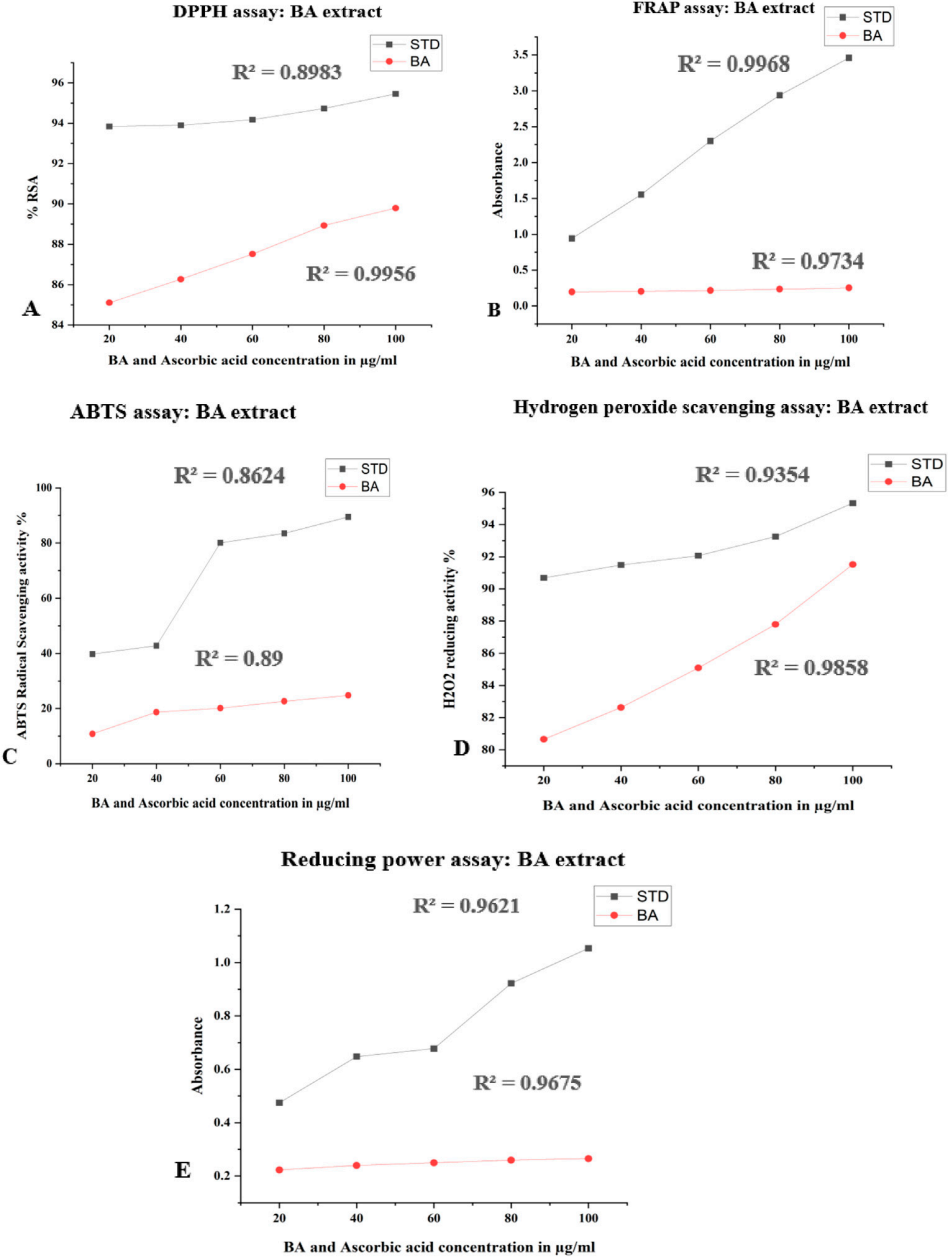
Antioxidant activity. **(A)** BA showed a significant increase in RSA, and a maximum % RSA was reported at 100 μg/mL. Values were represented as the mean ± S.D. **(B)** Ferric reduces the antioxidant power of BA. A significant increase in the absorbance was observed as the concentration of BA and the standard drug increased. Values were represented as the mean ± S.D. **(C)** ABTS, scavenging activity. BA with AA (standard drug), an antioxidant property to scavenge ABTS^+^ radical, increases in a concentration-dependent manner. All values were represented as the mean ± S.D. **(D)** BA showed H_2_O_2_-lowering action, increasing in a concentration-dependent way. Values were represented as the mean ± S.D. **(E)** BA showed an efficient increase in reducing power (Fe^3+^ to Fe^2+^) in a concentration (20–100 μg/mL)-dependent manner. Values were represented as the mean ± S.D.

The FRSA of BA was determined using the DPPH assay, which demonstrated effective radical scavenging activity (RSA) that increased with concentration, as illustrated in Figure 5A. BA reduced the DPPH solution’s purple colour to a yellowish colour by donating electrons to the free radicals. BA achieved an IC_90_ value of 100 μg/mL for scavenging free radicals, whereas the standard AA showed an IC_90_ value of <20 μg/mL.

### 3.17 FRAP assay

The analysis was performed to further evaluate the BA antioxidant capacity, expressed as ferrous sulphate equivalents (mM/g of sample). As shown in Figure 5B, BA exhibited increasing ferric-reducing antioxidant activity (Fe^3+^ to Fe^2+^) in a dose-dependent manner. The recorded absorbance values were 0.196, 0.203, 0.216, 0.234, and 0.252 at 20–100 μg/mL concentrations.

### 3.18 ABTS assay

The RSA of the BA was evaluated using the ABTS method. The scavenging activity increased with higher concentrations of BA and the standard. BA demonstrated an antioxidant capacity to scavenge ABTS^+^ radicals, ranging from 10% to 24%. In contrast, PC, AA (20–100 μg/mL), exhibited an RSA between 39%–89% and BA 10%–24%, as shown in Figure 5C.

### 3.19 H_2_O_2_ scavenging capacity

The capacity of BA to scavenge H_2_O_2_ is illustrated in Figure 5D. The extract demonstrated effective scavenging of hydrogen peroxide free radicals in a concentration-dependent manner (20–100 μg/mL), achieving its highest activity with an IC90 value of 100 μg/mL.

### 3.20 Reducing power assay

The results of evaluating the BA’s ability to convert Fe^3+^ to Fe^2+^ are depicted in Figure 5E. The findings indicated that the reducing power of BA increased significantly with dosage (20–100 μg/mL).

### 3.21 Haemolysis assay

**FIGURE 6:**
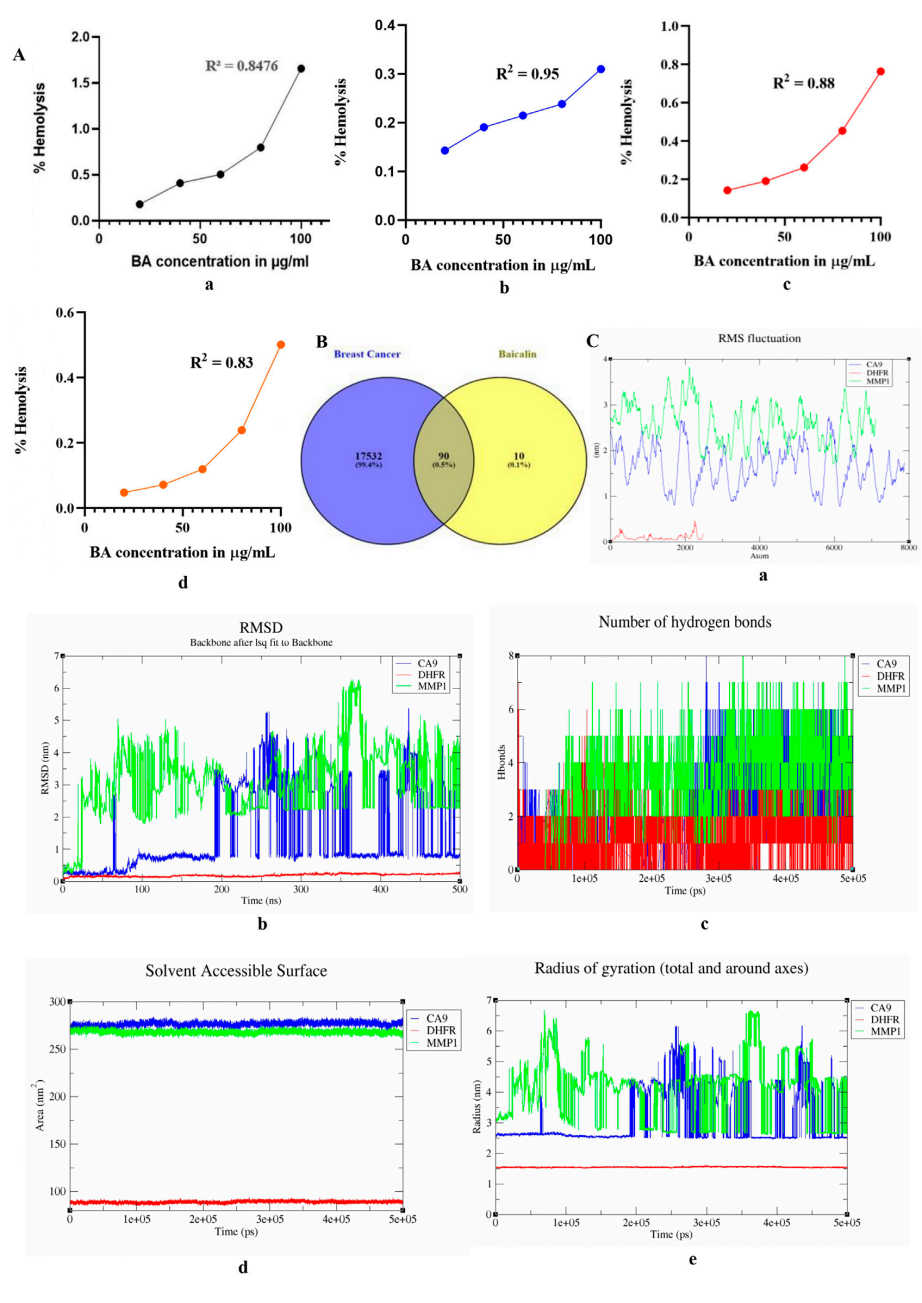
**(A)** Biocompatibility analysis of BA using a haemolysis assay (a–d). Graphical representation of the % haemolysis of BA (20–100 μg/mL) showed a reduction in RBCs. The data were represented as the mean ± S.D., with a significant ***p-value (<0.0001). **(B)** Venn diagram illustrating BA and BC-associated targets, which highlights their overlapping molecular pathways. **(C)** Analysis of the molecular dynamics simulation parameters over a 500-nanosecond timeframe for the proteins CA9, DHFR, and MMP1. (a) Root Mean Square Fluctuation (RMSF) values highlight the flexibility and movement of individual amino acid residues within the protein structures. (b) Root Mean Square Deviation (RMSD) profiles depict the overall structural stability of each protein throughout the simulation. (c) Total number of hydrogen bonds formed during the simulation, reflecting the stability and intermolecular interactions within the protein structures. (d) Solvent-accessible surface area (SASA) analyses reveal changes in protein surface exposure to the solvent environment. (e) Radius of gyration (Rg) measurements provide insight into the compactness and folding behaviour of the proteins over time.

*In vitro* testing was performed to assess the biocompatibility of BA and its safety for biological use. Fresh erythrocytes (red blood cells, RBCs) were exposed to various concentrations of BA (20–100 μg/mL). The results showed varying levels of haemolysis, with percentages of (0.178 ± 0.0015, 0.409 ± 0.007, 0.503 ± 0.006, 0.797 ± 0.009, and 1.65 ± 0.001), (0.143164, 0.190885, 0.214746, 0.238607, 0.310188), (0.143164, 0.190885, 0.262467, 0.453352, 0.763541), and (0.047721, 0.071582, 0.119303, 0.238607, 0.501074) at the respective concentrations, as illustrated in Figures 6A (a–d). BA demonstrated good biocompatibility as it did not cause significant erythrocyte lysis or haemoglobin release into the extracellular environment. This was confirmed by the centrifugation step and absorbance measurements (λ = 540 nm).

### 3.22 Pharmacokinetic study of baicalin

The PubChem database was used to collect information on BA’s chemical composition. Information, such as its widely used name, compound CID, and canonical structural SMILES, was used to identify potential targets through the related repository. ADMET analysis revealed that, despite low solubility, oral drug absorption, and poor melanoma permeation, BA is a critical drug indicator. The results in Supplementary Table S3 indicated that BA had low GI absorption. Additionally, BA did not inhibit any of the five cytochrome (CYP) subtypes essential for drug excretion. The toxicological profile demonstrated that BA was non-toxic to the liver or skin. The findings on drug-likeness and medicinal chemistry support the potential of BA as a drug candidate.

### 3.23 Evaluation of potential targets

By analysing the targets screened from the GeneCards database, a total of 17,622 targets were recognised as being linked to BC. Upon merging these with the 100 targets of BA through Venny 2.1.0, 16 common targets were predicted as potential candidates, as depicted in Figure 6B.

### 3.24 PPI network construction and analysis

After integrating the STRING database, the protein interaction mapping network was imported into Cytoscape 3.7.1. As shown in Supplementary Table S2, DHFR (degree = 13), CA9 (degree = 12), and MMP1 (degree = 11) were selected at higher degrees than the median degree = 8.

### 3.25 Active site prediction

Active site prediction was carried out by identifying structural cavities. This approach operates on the premise that key active sites, including binding regions, tend to be structurally and locationally preserved, including various proteins. As a result, the forecast sites are considered significant for binding interactions, which play a crucial role in drug design and functional analysis. The CASTp tool was employed to identify multiple potential active sites on proteins, with each site evaluated based on surface cavity size to determine the most suitable one. Detailed information on these identified binding sites, including specific residues, is provided in Supplementary Table S4.

### 3.26 Molecular docking and analysis

The top 25 targets for molecular docking were selected based on protein interaction mapping. The docking scores and the interactions of BA with these targets are provided in Supplementary Table S5. The systematic analysis of the PPIs and molecular docking scores identified CA9 (PDB ID: 6Y74), DHFR (PDB ID: 1RF7), and MMP1 (PDB ID: 3SHI) as the top three targets.

### 3.27 MDS analysis

The 500 ns MDS was assessed using GROMACS. The trajectories for RMSD, RMSF, Rg, and H-bond were obtained, indicating the overall stability, flexibility, rigidity, and compactness of the system. The SASA was also analysed, suggesting structural or binding site changes as shown in Figures 6C (a–e).

### 3.28 Cell viability assay

**FIGURE 7:**
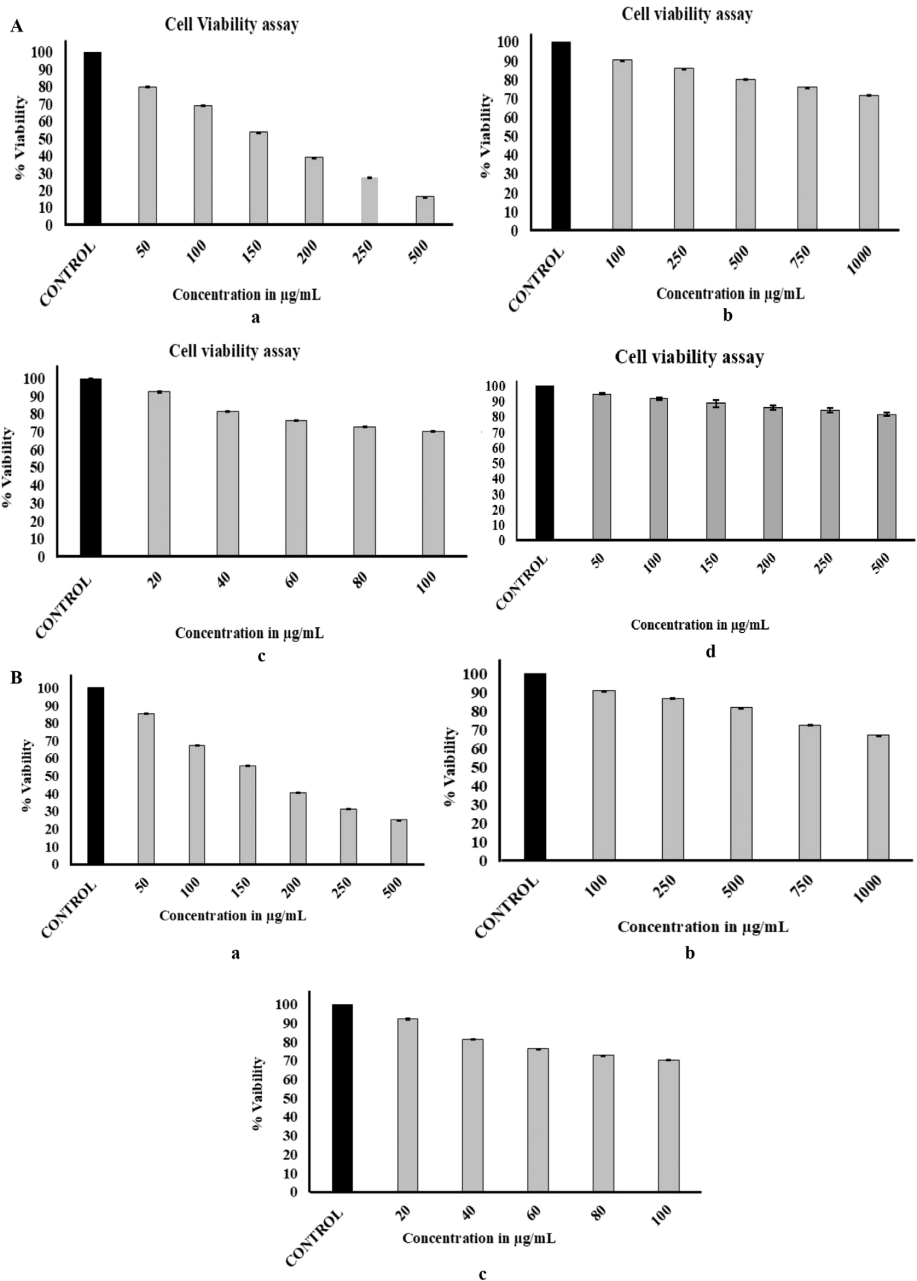
**(A)** Cell viability assay representing the cytotoxicity profiles of BA and 5-FU on MCF-7 BC cells, L929 normal fibroblast cells, and PBMCs. (a) The cytotoxic effect of various concentrations of BA on MCF-7, the dose-dependent reduction in cell viability, indicates significant cytotoxic potential of BA against cancer cells. (b) Cytotoxicity of BA on L929 normal fibroblast cells, representing healthy cells, to assess the biocompatibility and selective cytotoxicity of BA. Results show comparatively lower cytotoxic effects on normal cells, indicating selective targeting of cancer cells by BA. (c) The cytotoxic profile of the standard anti-cancer drug 5-FU was used as a positive control on MCF-7 cells. (d) The results demonstrated negligible cytotoxicity of BA on PBMCs, indicating its admirable biocompatibility and selectivity towards cancer cells over normal immune cells. **(B)** NRU assay showing the cytotoxic effects of BA and 5-FU on MCF-7 cells and L929 normal fibroblast cells (a) BA exhibited a dose-dependent decrease in the viability, indicating its strong cytotoxic potential against BC cells. (b) In L929 normal fibroblast cells, BA showed minimal cytotoxicity, suggesting good biocompatibility and selective action toward cancer cells. (c) 5-FU, used as a standard drug, also showed expected cytotoxic effects on MCF-7 cells and served as a positive control. Data are expressed as the mean ± SD (n = 3), and statistical significance was analysed using a one-way ANOVA, followed by Tukey’s *post hoc* test (*p* < 0.05).

The CV assay showed that BA and 5-FU suppressed the growth of MCF-7 BC cells in a concentration-dependent manner, with IC_50_ values calculated at 160 μg/mL and 410 μg/mL, respectively. In the case of L929 healthy mouse fibroblast cells and PBMCs, a similar decreasing trend was observed; however, the IC_50_ value was significantly higher at 2 mg/mL and 1.5 mg/mL, respectively. This indicates that BA is more toxic to cancer cells than to normal cells. The results are presented in Figures 7A (a–d).

### 3.29 Neutral red cellular uptake assay

The cytotoxic response of BA as observed by the NRU assay is shown in Figures 7B (a–c). The cells treated with various concentrations of BA decreased the CV in a dose-dependent manner. Similar to the MTT assay, cells exposed to BA exhibited less toxicity compared to 5-FU and were safer for healthy cells, L929.

### 3.30 Cell morphology analysis

**FIGURE 8:**
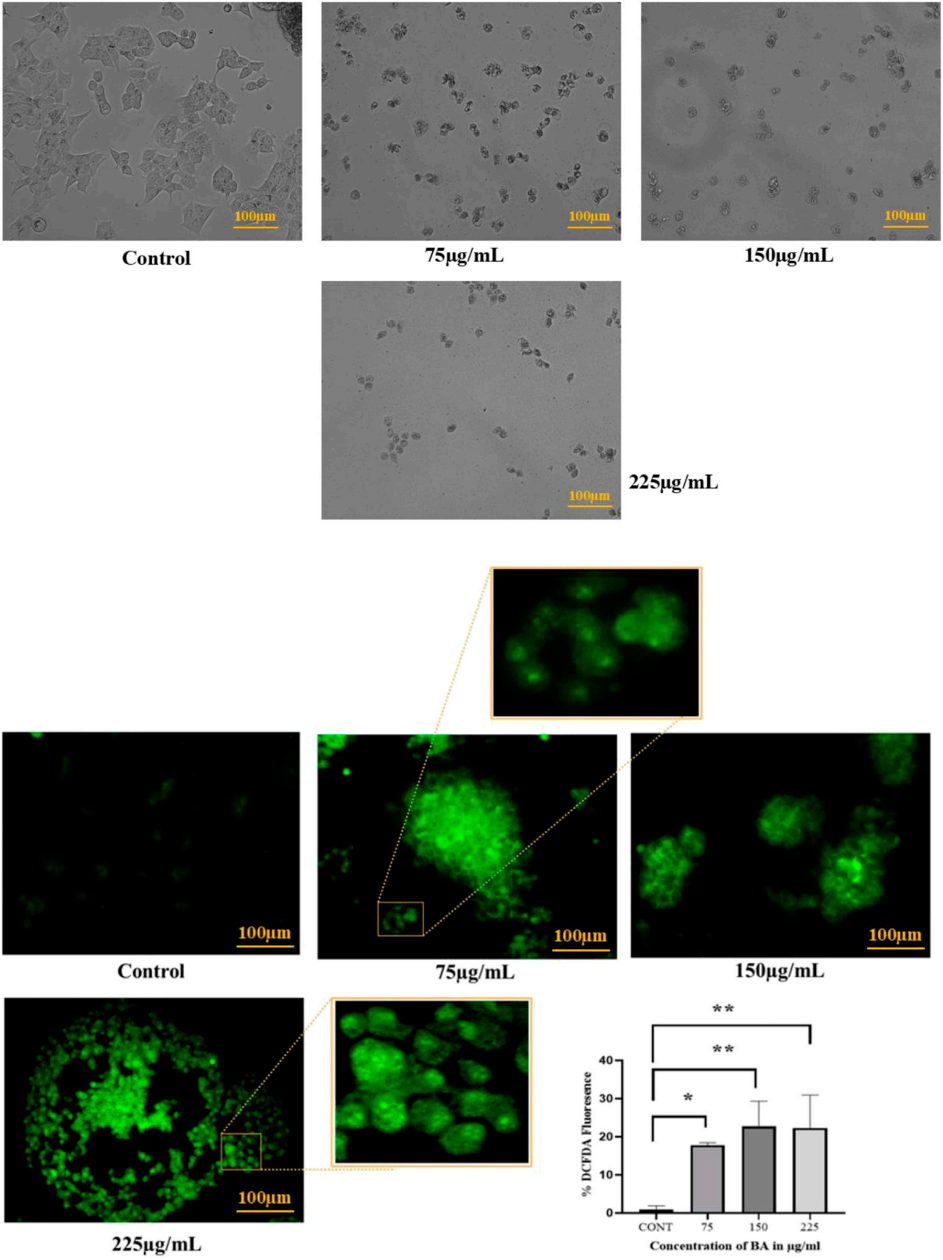
**(A)** Morphological changes in MCF-7 cells were observed under a microscope after treatment with BA. Cells exhibited the most distinct changes at the highest concentration, indicating significant cytotoxic effects. **(B)** ROS production in MCF-7 cells was observed under a fluorescence microscope after treatment with BA. A dose-dependent increase in ROS levels was noted, with higher concentrations of BA inducing stronger fluorescence signals. This indicates an increase in oxidative stress within the cancer cells. Untreated cells showed minimal ROS generation.

Morphological changes in cells were visually identified using the fluorescent imaging microscope after the exposure of MCF-7 cells to various aliquots of BA. At the highest concentration, the cells lost their normal elongated shape and were almost spherical, as shown in Figure 8A.

### 3.31 ROS generation

A progressive increase was observed with increasing concentrations of BA, resulting in an increase in ROS levels. Increased ROS accumulation is reported to cause oxidative stress, which has the potential to induce apoptotic processes in cancer cells. Fluorescence intensity, as a marker of ROS levels, was maximum at the highest dose, indicating increased oxidative damage and high pro-apoptotic activity at high concentrations, as shown in Figure 8B.

### 3.32 Determination of nuclear morphology with Hoechst 33342 staining

**FIGURE 9:**
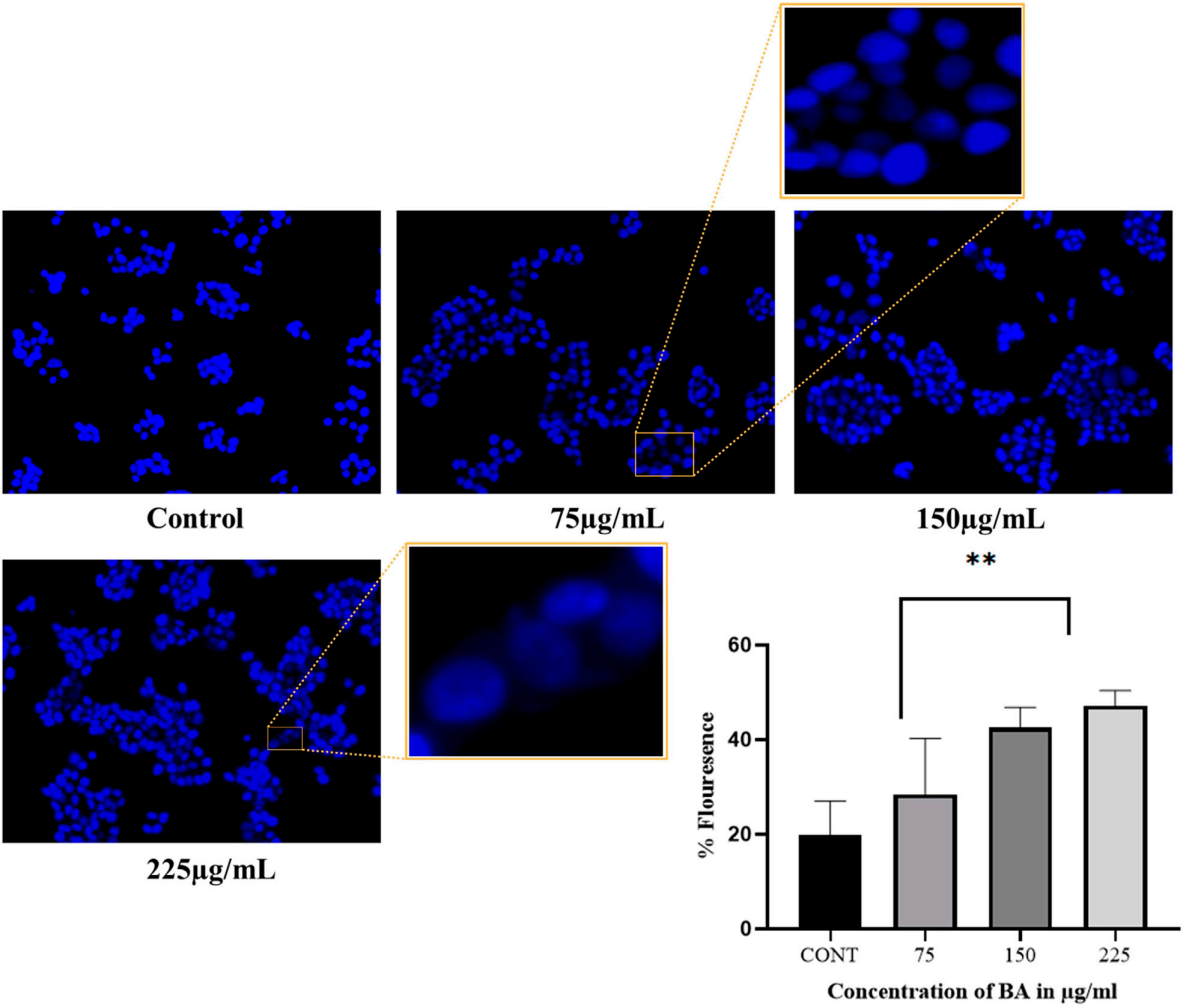
Nuclear morphological alterations in cells were visualised by Hoechst 33342 staining after treatment with BA. A dose-dependent increase in chromatin condensation and nuclear fragmentation was observed, indicating apoptosis.

The effect of BA on MCF-7 cells was assessed by Hoechst staining, which is a dye that binds specifically to nuclear DNA. In Figure 9, clear nuclear morphological changes were shown after treatment. These changes, such as condensation and fragmentation of nuclei, increased with higher concentrations of BA, suggesting dose-dependent induction of nuclear damage.

### 3.33 Apoptotic cell identification using propidium iodide staining

**FIGURE 10:**
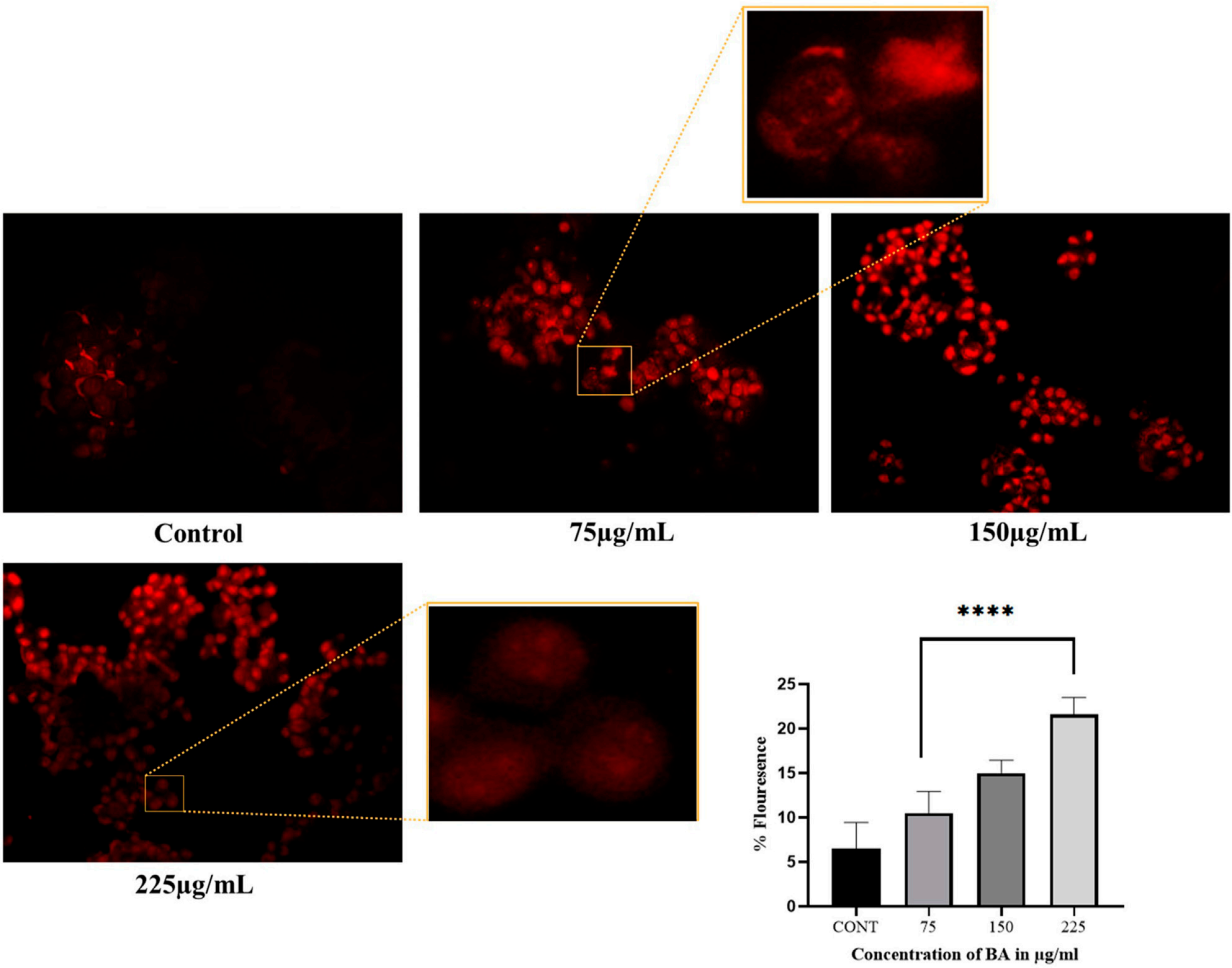
PI staining was used to evaluate membrane integrity in cells after BA treatment. A dose-dependent increase in red fluorescence was observed, indicating loss of membrane integrity and late-stage apoptosis or necrosis. Higher concentrations of BA resulted in a greater number of PI-positive cells. Untreated control cells showed minimal or no PI uptake.

PI is a cell-permeant dye that specifically stains the DNA of apoptotic or dying cells, producing red fluorescence. It is unable to penetrate the intact membranes of live cells; fluorescence in untreated controls is minimal, reflecting the integrity of the membranes. We also have a distinct, dose-related increase in PI fluorescence intensity (Figure 10). This progressive increase in fluorescence indicates that as the concentrations of the treatment increased, progressively more cells were unable to maintain membrane integrity, culminating in cell death.

### 3.34 Assessment of lysosomal activity *via* LysoTracker Red DND-99 staining

**FIGURE 11:**
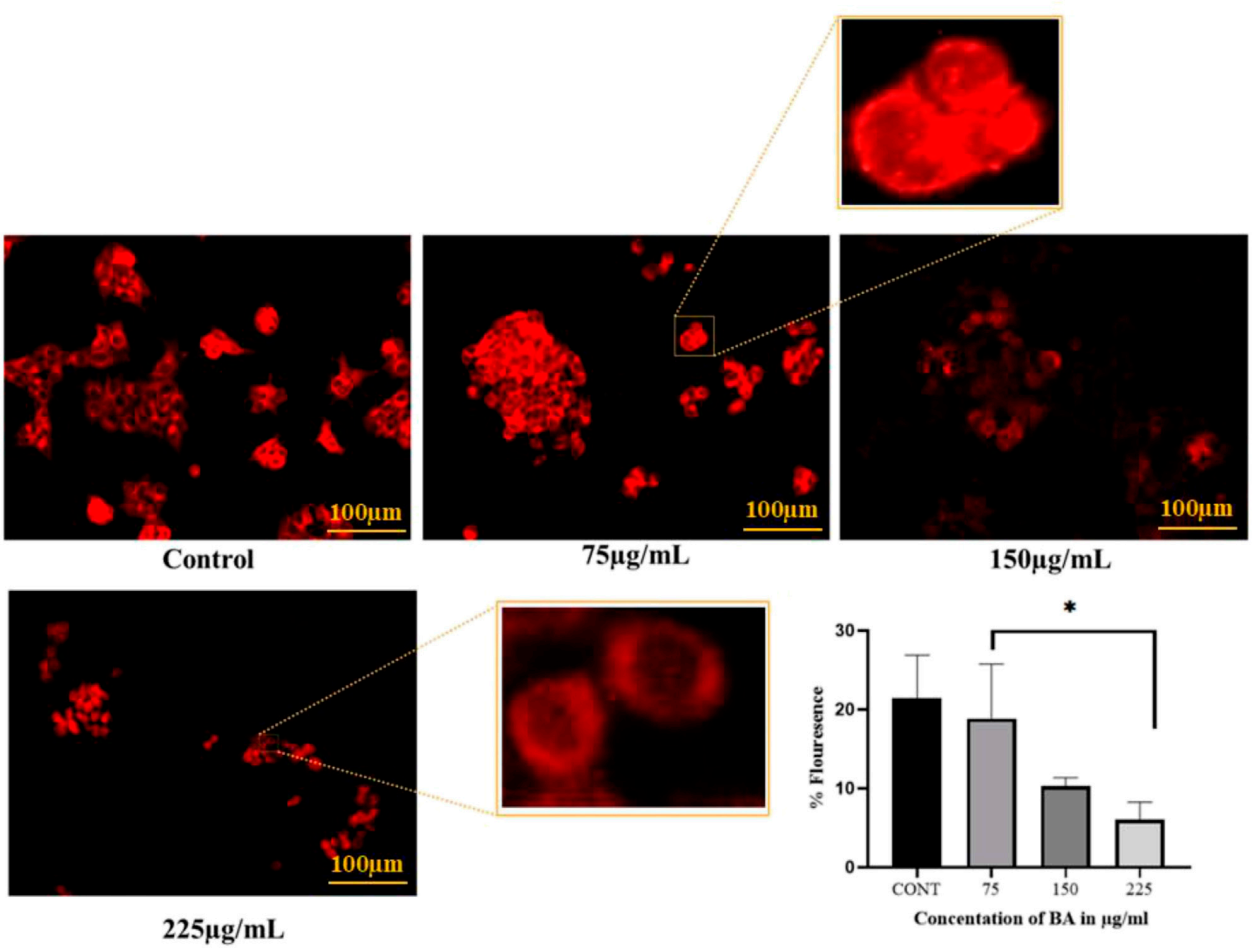
Lysosomal activity in MCF-7 cells was assessed using LysoTracker Red DND-99 staining after treatment with BA. In control cells, lysosomal integrity remained intact, resulting in bright LysoTracker Red fluorescence. However, with increasing concentrations of BA, lysosomal membrane integrity was compromised, resulting in a reduction in fluorescence intensity. This dose-dependent decrease indicates lysosomal destabilisation and impaired lysosomal function in treated MCF-7 cells.

To assess lysosomal integrity in BA-treated cells, LysoTracker Red DND-99 dye was used, which is specific for staining acidic compartments such as lysosomes. In controls, intense red fluorescence was present, reflecting functional and intact lysosomes. However, with enhanced BA concentrations, a significant, dose-dependent decrease in fluorescence intensity was evident. This reduction indicates a disruption in lysosomal acidity, which is presumably caused by pH changes during apoptosis, suggesting lysosomal implication in BA-induced cell death, as shown in Figure 11.

### 3.35 MitoTracker Red CMX-ROS and Hoechst 33342 merge staining

**FIGURE 12:**
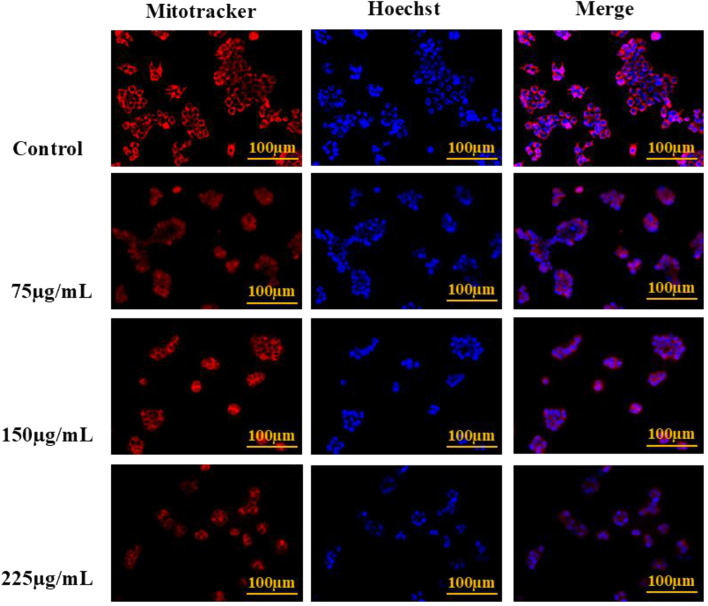
Mitochondrial and nuclear alterations in MCF-7 cells after BA treatment, visualised using MitoTracker Red CMX-ROS and Hoechst 33342 staining. MitoTracker Red staining showed a dose-dependent reduction in mitochondrial membrane potential, evident by decreased red fluorescence intensity in treated cells. Hoechst 33342 staining revealed nuclear condensation and fragmentation, indicating apoptosis. The merged images confirm mitochondrial dysfunction alongside nuclear damage in BA-treated cells compared to untreated cells with intact mitochondria and normal nuclei.

Mitochondrial membrane potential and nuclear morphology were assessed using MitoTracker Red CMX-ROS and Hoechst 33342 dual staining. MitoTracker selectively accumulates in active mitochondria due to their intact negative membrane potential, emitting bright red fluorescence, while Hoechst stains the nuclei by binding to DNA, emitting blue fluorescence. In untreated control cells, intense red mitochondrial staining with well-defined blue nuclei indicated healthy mitochondrial function and intact nuclear morphology. We found a dose-dependent reduction in MitoTracker fluorescence and disruption of mitochondrial membrane potential, and Hoechst staining revealed nuclear condensation and fragmentation at higher concentrations, indicative of apoptosis, as shown in Figure 12.

### 3.36 Monitoring mitochondrial membrane potential using JC-1 fluorescent staining

**FIGURE 13:**
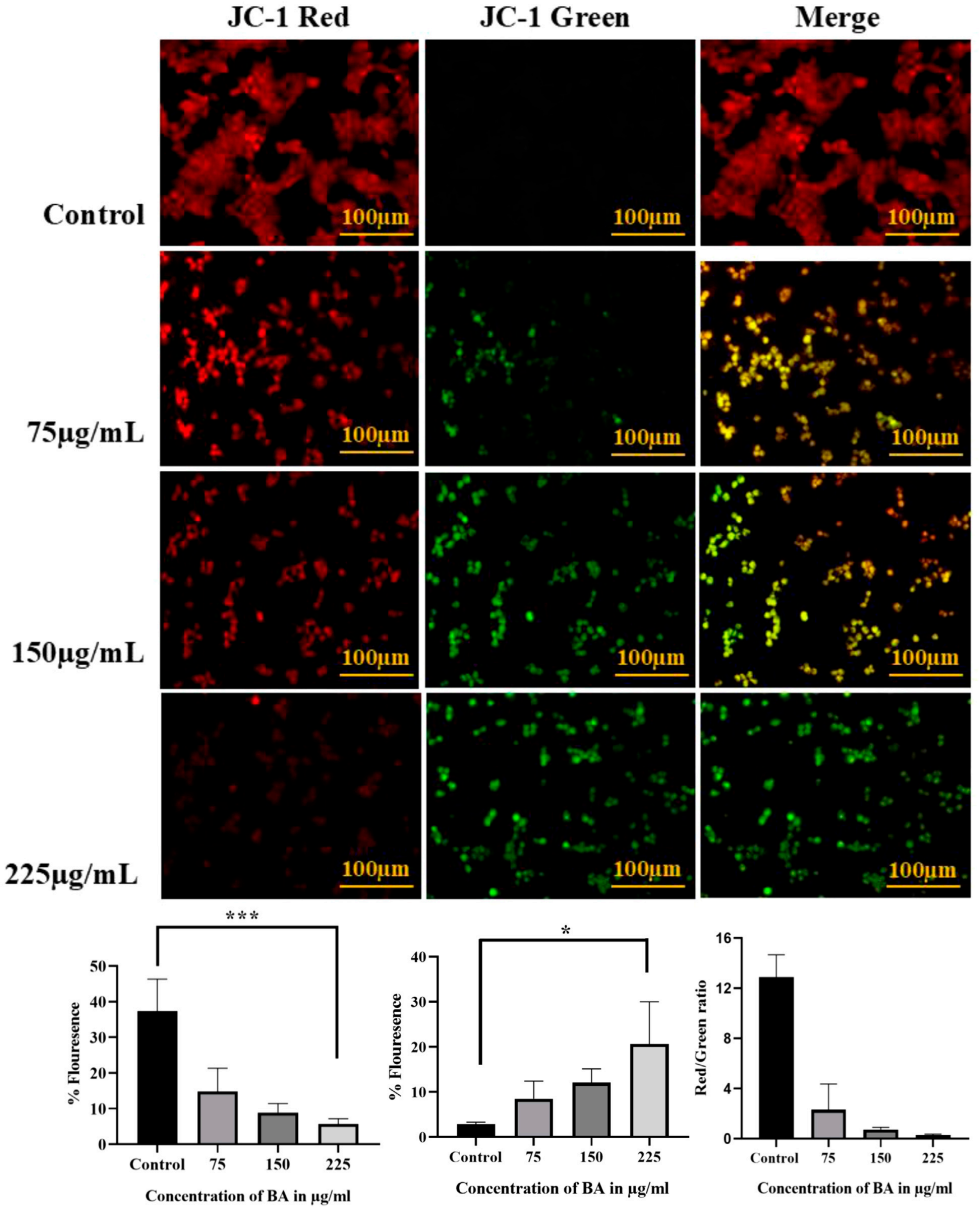
MMP (ΔΨm) changes in cells after BA treatment, evaluated by JC-1 dye staining. In untreated cells, strong red fluorescence was observed due to the formation of JC-1 aggregates in healthy mitochondria. With increasing BA concentrations, a shift from red to green fluorescence was detected, indicating mitochondrial depolarisation. The merged images show a dose-dependent increase in the green-to-red ratio, confirming the loss of mitochondrial membrane potential and early apoptotic events.

MMP (ΔΨm) was measured with JC-1 dye, a potent fluorescent probe that is accumulated in mitochondria in a membrane potential-dependent manner. In healthy cells, JC-1 forms aggregates in the mitochondria, producing red fluorescence. In contrast, in mitochondria-dysfunctional cells, it exists as a monomer, exhibiting green fluorescence. Under control cells, intense red fluorescence with minimal green signal demonstrated good mitochondrial function. With progressively higher levels of BA, however, a distinct concentration-dependent transition from red to green fluorescence was observed, indicative of progressive loss of mitochondrial membrane potential, a key early sign of apoptosis, as illustrated in Figure 13. The control group showed a ratio of 12.95, whereas baicalin-treated cells yielded lower ratios of 1.74 (75 μg), 0.74 (150 μg), and 0.28 (225 μg).

### 3.37 Visualisation of early and late apoptosis using acridine orange/ethidium bromide dual staining

**FIGURE 14:**
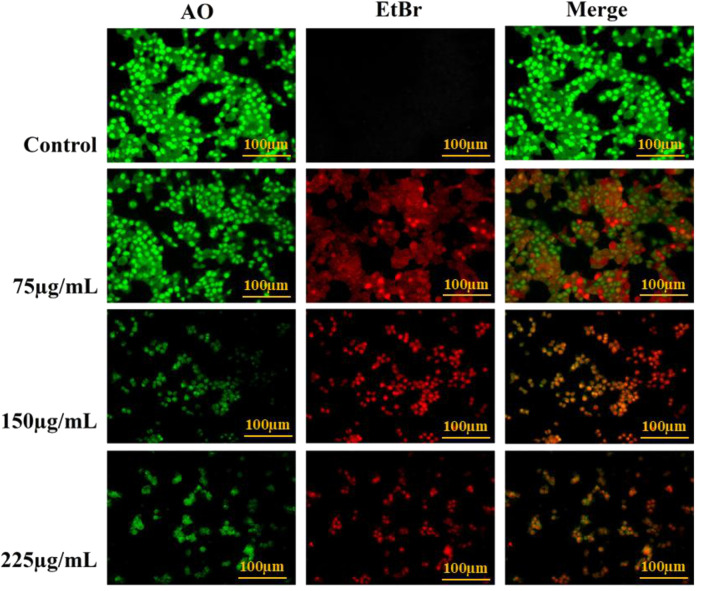
Detection of early and late apoptotic cells in MCF-7 cells after BA treatment using AO/EtBr dual staining. A dose-dependent increase in apoptotic cell population was observed. Early apoptotic cells exhibited green fluorescence with condensed chromatin, while late apoptotic and necrotic cells showed orange to red fluorescence due to EtBr uptake. Untreated cells displayed uniform green fluorescence with intact nuclei, indicating that they were healthy and viable.

AO/EtBr double staining is an efficient and commonly applied method used to differentiate between early and late apoptotic cells. AO can penetrate all cells, staining them green, but EtBr can enter only cells with damaged membranes, staining them red. In this double-staining technique, early apoptotic cells are stained a yellowish-orange colour as a result of partial EtBr uptake, while late apoptotic cells are stained a bright orange to red colour. We observed a dose-dependent decrease in green fluorescence and the emergence of yellow- to red-stained cells, indicating the progressive induction of apoptosis with increasing doses of BA, as shown in Figure 14. The findings clearly indicate a dose-dependent decrease in mitochondrial membrane potential after BA treatment.

### 3.38 Cell migration assay

**FIGURE 15:**
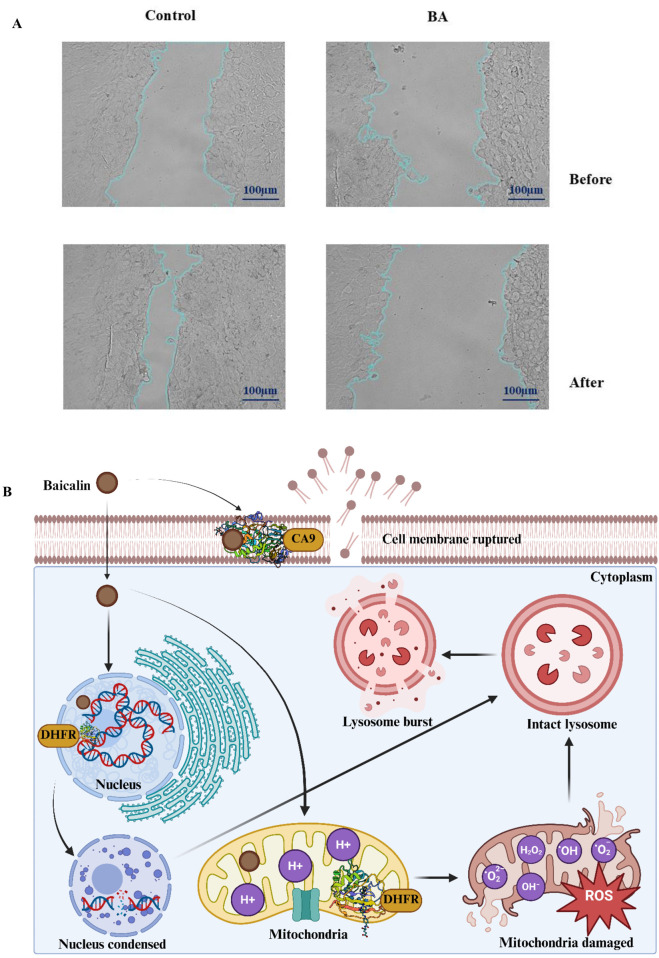
**(A)** Effect of BA on MCF-7 cell migration as assessed by the scratch assay. Images of wound healing at 0 h and 24 h in untreated and BA-treated groups. In the control group, the wound area decreased from 34% to 11%, indicating active cell migration and wound healing. The BA-treated group (IC_50_ concentration) showed an increase in wound area from 43% to 59% after 24 h, demonstrating significant inhibition of cancer cell migration. **(B)** Baicalin-mediated cell death in breast cancer-derived MCF-7 cells. BA initially disrupts the cell membrane by interacting with Carbonic Anhydrase IX (CA9), as indicated by in silico studies. CA9 is located within the plasma membrane of cancer cells and may lead to the dysregulation of acidic pH levels in these cells. Following its translocation to the cytoplasm, BA is transported to the nucleus, where it interacts with genetic material, resulting in DNA damage and promoting nuclear condensation. In silico analyses demonstrate that BA also interacts with Dihydrofolate Reductase (DHFR), which might adversely affect the DNA synthesis process and induce DNA damage-mediated cell death. Concurrently, BA also impairs mitochondrial functionality by elevating oxidative stress through its interaction with both DHFR and proton pumps. It also increases the mitochondrial permeability and lowers the mitochondrial membrane potential, driving the cell to death. Collectively, these mechanisms drive the cell toward apoptosis, culminating in lysosomal rupture and the subsequent death of the cancer cell.

The assay was executed to evaluate the anti-migratory effect of BA on MCF-7 BC cells. At 0 h, the control group’s initial wound area was approximately 34%, which decreased considerably to 11% after 24 h of incubation, indicating a high level of wound healing through active cell migration. BA treatment at an IC_50_ concentration had an original wound area of approximately 43%, which increased to approximately 59% after 24 h, as shown in Figure 15A. This indicates that BA strongly suppressed cell migration and proliferation, hindering wound healing and thus expanding the scratch area in a time-dependent manner. These findings suggest that BA exhibits strong anti-migratory activity against MCF-7 cells, indicating its potential in inhibiting cancer cell metastasis.

## 4 Discussion

Breast cancer remains one of the most life-threatening cancers in women worldwide. Even though traditional treatments such as chemotherapy and radiation are used, they are usually accompanied by serious side effects, high toxicity, and drug resistance. Natural products have emerged as promising alternatives in this regard as they exhibit multi-targeted activities, lower toxicity, and improved biocompatibility. One such natural compound is BA, a bioactive flavonoid isolated from *Scutellaria baicalensis*, which was profiled in this study using an integrated *in vitro* and *in silico* method to assess its biochemical profile and anti-breast cancer activity. In this study, BA has been effectively extracted by employing three phytoextraction techniques: probe sonication, bath sonication-assisted extraction, and maceration. Of these, the maximum recovery of 32% was achieved using 60% methanol in ultrasound probe sonication. The increased recovery can be attributed to the improved mass transfer and solvent penetration due to the ultrasonic waves. Our results are consistent with earlier findings of Lee et al. (2014), who reported a maximum yield of 43% was maximum when 60% ethanol was used, and Yang et al. (2013), who reported a yield of 50% for the same concentration of the solvent. These studies lend support to the hypothesis that moderate aqueous methanolic solvent concentrations, such as 60%, are ideal for extracting polar bioactive compounds, including BA. UV–VIS spectroscopy indicated characteristic absorption peaks for BA in the range 280–285 nm, corresponding to the presence of conjugated flavone chromophores. This finding is consistent with previously reported results, which show similar peak profiles for BA at 277 nm (Yang et al., 2012). FT-IR spectroscopy confirmed the presence of major functional groups in BA, with strong absorption bands corresponding to hydroxyl and aromatic groups, characteristic of the flavonoid structure. The same finding is reported by Dmitri et al. (2023) with similar FT-IR peaks identified in their research on BA, thus validating our results. Zeta potential and particle size analysis identified a moderately negative surface charge of −9.302 mV and nanoscale particle size. The negative zeta potential is probably attributed to the presence of ionizable functional groups, especially carboxyl and hydroxyl groups, on the surface of BA or its conjugated phytochemicals’ surface. Such groups would tend to deprotonate under aqueous conditions, which contributes to the overall negative surface charge (Gulati et al., 2024; Yadav et al., 2023a). A zeta potential value within this range, though not strongly negative, nonetheless holds enough electrostatic repulsion to obviate particle agglomeration and improve colloidal stability. This was also evidenced by a PDI of less than 0.3, which indicates the presence of uniform and homogeneously distributed particles. In drug delivery and BC treatment, a negatively charged surface is preferred for long-term circulation in the blood as it can minimise opsonisation and the resultant elimination by the reticuloendothelial system (RES). Negatively charged particles have also demonstrated greater tumour accumulation due to the enhanced permeability and retention (EPR) effect, which makes them attractive carriers for targeted cancer therapy (Yadav et al., 2023b). HPTLC analysis confirmed the presence of BA through a comparison of retention factor (Rf) values. The Rf value observed for the sample was 0.33, closely aligning with the standard Rf value of 0.32 for BA, thereby validating its identification. These findings are consistent with earlier research conducted by Adin et al. (2022), which reported an Rf value of 0.49 for BA. Our study explores the HPLC profiling of BA, which shows a retention time of approximately 17.691 min, very close to the standard for BA, 17.647 min, thereby ascertaining both its presence and high purity. This finding validates the previously reported findings of Yang et al. (2013), which showed a retention time of 16.64 min for BA. LC–MS/MS in positive ion mode also confirmed the structural identity of BA. A precursor at m/z 447.0928 yielded a dominant fragment at m/z 271.0604, indicative of the loss of glucuronide and formation of BE. The fragmentation was consistent with reported studies (Chung et al., 2012), supporting our interpretation. XRD analysis showed a wide peak between the 2θ values of 6°–18°, which indicated the amorphous character of BA. The amorphous nature of BA is preferable in drug formulations as amorphous materials exhibit greater solubility and bioavailability (Li et al., 2018). ^1^H and ^13^C NMR spectroscopy provided clear structural evidence of BA. Aromatic region chemical shifts (6–8 ppm) and glucuronic acid protons (3–5.5 ppm) were definitely present, whereas ^13^C signals supported the flavone skeleton and sugar part. The values were consistent with literature-reported ^1^H and ^13^C NMR data (Zhang et al., 2016). SEM examination showed irregularly shaped particles with a smooth surface morphology, which can enable cellular uptake. A delicate aggregation has been observed, which could be attributed to the drying process during sample preparation. The study quantifies the presence of secondary metabolites through TPC at 99.9 ± 0.27 mg GAE/g, TFC at 80.4 ± 2 mg QE/g, and TFolC at 79.4 ± 2 mg QE/g, suggestive of a flavonoid-rich profile of BA, as reported by several studies, along with some protein content in BA itself. BA exhibited strong antioxidant activity in various antioxidant assays, which clearly resembles previously reported studies conducted by Peng-Fei et al. (2013). The DPPH assay reconfirmed strong radical scavenging ability with an IC_90_ < 20 μg/mL. The combination of FRAP, ABTS, and RPA assays effectively demonstrated the strong antioxidant activity of BA by assessing its ability to donate electrons or hydrogen atoms for scavenging ROS radicals. Specifically, the FRAP assay evaluates BA’s reducing capability as it can reduce Fe^3+^ to Fe^2+^, providing a reliable index of redox potential. The ABTS assay measures BA’s effectiveness in quenching ABTS^+^ radicals, which parallels the conditions of oxidative stress typically found in biological systems. Additionally, the RPA assay further substantiated the electron-donating properties of BA, highlighting its overall antioxidant efficacy. Significantly, H_2_O_2_ scavenging activity revealed effective neutralisation of ROS, re-asserting its capability in eliminating oxidative stress, a characteristic of cancer progression. BA also had moderate anti-inflammatory activity, as suggested by its capacity to scavenge inflammatory mediators *in vitro*, further supporting its role as a chemopreventive agent. The haemolysis assay showed low hemolytic activity, from 0.17% to 1.65% over concentrations of 20–100 μg/mL, indicating BA’s superior biocompatibility and non-toxicity towards normal red blood cells. To unravel the molecular mechanisms, a network pharmacology and molecular docking strategy is employed. PubChem, GeneCards, STRING, and SwissTargetPrediction assisted in the identification of crucial BC-related targets for BA. These targets were selected for their roles in regulating cancer cell proliferation, apoptosis, and metastasis. Molecular docking demonstrated CA9, DHFR, and MMP1 as the top three targets, showing good binding affinities (in kcal/mol) of BA with all the chosen proteins. Hydrogen bonds and hydrophobic interactions are suggested to be stable and specific ligand–receptor interactions. Prediction of a binding site by CASTp helped define active pockets important for BA binding. Molecular dynamics simulation (500 ns) confirmed the stability of BA–protein complexes. The RMSD analysis of CA9, DHFR, and MMP1 with BA during a 500 ns simulation of the DHFR revealed favourable stability ranging between 1 and 500 ns. However, CA9 was stable initially for 55 ns, and 100–180 ns later, it exhibited drastic deviations. However, MMP1 did not show any stability throughout the 500 ns simulation. For Rg and SASA analysis, the complexes displayed SASA values of DHFR, CA9, and MMP1 ranging from 50 to 60 nm, 275–285 nm, and 260–275 nm, respectively, while Rg values ranged between 1.5 Å, 2.6 Å, and 3.2 to 6.5 Å, respectively. The SASA of DHFR was more stabilised compared to CA9 and MMP1. The H-bond analysis of CA9 and MMP1 revealed that they initially formed 6–7 hydrogen bonding interactions and later formed a maximum of eight hydrogen bonding interactions, which account for their strong affinity. DHFR initially showed seven hydrogen bonds and later showed only three hydrogen bonds, and RMSF analysis offered insights into the residue stability of the examined complexes, revealing that the newly discovered compounds demonstrated enhanced residue stability. The analysis of these parameters indicates that the complexes remain structurally compact throughout the trajectory. Overall, MDS revealed that the protein–ligand complex of DHFR–BA is more stable compared with CA9 and MMP1.

Pharmacokinetic profiling of baicalin holds several discrepancies that limit its efficacy in the body. Extensive first-pass metabolism, poor bioavailability, and an ephemeral half-life, mainly due to the glycosyl group present in its ring structure. As a glycosidic flavone, baicalin is highly polar, which limits its transmembrane passive diffusion, resulting in poor absorption in the intestinal tract. Its aglycone counterpart, baicalein, is more lipophilic in nature and thus possesses far greater permeability and absorbability in the gastrointestinal tract. Several pharmacokinetic studies have demonstrated that baicalin involves complex processes in the body, including hydrolysis within the gastrointestinal tract, enterohepatic cycling, carrier-mediated transport across membranes, systemic metabolism, and eventual excretion *via* bile and urine (Huang et al., 2019). β-Glucuronidase enzymes from intestinal bacteria quickly hydrolyse baicalin upon oral administration to yield baicalein. Upon absorption, baicalein is again converted into baicalin by UDP-glucuronosyltransferase (UGT) enzymes present in systemic circulation. Since baicalein is better absorbed, such a conversion from baicalin to baicalein becomes a determining factor for the overall absorption of baicalin. Lai et al. (2003) exemplified the same: baicalin reached its peak plasma concentration (C_max_) after approximately 396 min, whereas baicalein attained C_max_ within 10 min, indicating that the hydrolysis of baicalin by gut enterobacteria is the rate-limiting step for its absorption (Lai et al., 2003). Due to the high polarity of baicalin, its disposition in the body is greatly influenced by carrier-mediated transport as opposed to passive diffusion. Zhang et al. (2007) and Kovács et al. (2015) reported that the transporters involved in the process are MRP3 and MRP4 as basolateral transporters and MRP2 and the breast cancer resistance protein (BCRP) as apical transporters.

MTT and NRU assays indicated dose-related cytotoxicity in MCF-7 BC cells with an IC_50_ value of 160 μg/mL. BA, on the other hand, had low toxicity against L929 fibroblast cells (IC_50_ = 2 mg/mL) and PBMCs, with an IC_50_ value of 1.5 mg/mL, emphasising its selective activity in targeting BC cells. The low cytotoxicity of PBMCs and L929 towards BA indicates that this compound has a minimal cytotoxic effect on healthy immune cells, consistent with previous findings that flavonoids with antioxidant and anti-inflammatory activities tend to preserve immune cell viability within pharmacologically relevant concentrations (Yang et al., 2021; Yan et al., 2023). Such biocompatibility with healthy cells is important for translational development as PBMCs are essential for immunomodulation and systemic responses in cancer therapy. The low toxicity observed also suggests that BA would not compromise immune integrity if it were used as a therapeutic adjunct, compared with many synthetic chemotherapeutics known to cause haematological toxicity. Hence, the PBMC assay results support the safety and possible clinical use of baicalin as a natural anticancer drug with low off-target cytotoxicity. The ROS generation was observed to be concentration-dependent in increasing the intracellular ROS levels, implying that BA triggers apoptosis *via* oxidative stress pathways; this mechanism is shown in Figure 15B. Further exploration of the mitochondrial contribution was conducted using JC-1 staining. JC-1 staining indicated a red-to-green shift of fluorescence in BA-exposed cells, signifying loss of mitochondrial membrane potential (ΔΨm), a characteristic of early apoptosis. Hoechst 33342 staining has revealed hallmarked nuclear morphological alterations in BC cells treated with BA, including chromatin and nuclear condensation and fragmentation, key indicators of apoptotic cell death. The nuclear alterations observed in our investigation corroborate the genotoxic effects of BA as Hoechst dye specifically binds to the minor groove of A–T-rich regions in DNA. These findings emphasise that BA not only promotes apoptosis but may also induce cell death *via* DNA damage, highlighting its potential importance in BC treatment, which totally correlates with the study conducted by Bernasinska-Slomczewska et al. (2024), suggesting that BA intercalates with dsDNA either directly or indirectly, resulting in genotoxic stress in cancer cells, which triggers apoptosis *via* the intrinsic pathway. Furthermore, our study delves into PI staining to confirm the cell membrane integrity in BA-treated cells, which is a characteristic feature of cell death (Kamble et al., 2022). PI is a DNA-intercalating, membrane-impermeable fluorescent dye that incorporates into the cell when membrane integrity is compromised. Our study showed that BA-exposed cells were highly PI-fluorescent, indicating that BA destabilises membrane integrity and induces cell death in BC cells. Our study also revealed that BA induces apoptosis and lysosomal destabilisation in BC cells, as indicated by the reduced fluorescence of LysoTracker Red DND-99 staining. To further confirm our study findings, AO/EtBr staining was performed, which revealed that the BC cells progressed from early to late apoptosis, indicated by a shift from green to orange-red nuclei upon increasing BA exposure. This finding completely correlates with a previously reported study (Palanivel et al., 2020). The results of Chen and Nalbantoglu (2014) correlate with the findings of our wound healing assay, which showed that BA impedes cell migration (wound size increased from 43% to 59%) in BA-exposed BC cells, compared to speedy wound recovery (wound size decreased from 34% to 11%) in BA-untreated cells. These results highlight the promising role of BA as an anti-metastatic agent and a potential drug candidate for BC treatment, inspiring further research and development in this area.

The *in vitro* data confirm that BA induces apoptosis in BC cells by generating ROS, lysosomal and mitochondrial damage, and nuclear injury. Its migration-inhibiting property further confirms its therapeutic value. Together with the selectivity for cancer cells, these observations highlight BA’s potential as a natural anti-breast cancer drug.

Pharmacokinetic profiling of BA has been conducted in several animal models. A tissue distribution experiment conducted in rabbits revealed that, following intravenous dosing, renal tissue contained the highest level of baicalin. When baicalin was administered in a liposomal formulation, it accumulated more in the lung tissue (Huang et al., 2019; Wei et al., 2016). Another study employing the Huang-Lian-Jie-Du-Tang herbal preparation in middle cerebral artery occlusion (MCAO) rats found that baicalin concentrations were highest in the lungs, rather than in the kidneys or liver, after oral dosing, which aligns with its major sites of action. This means that baicalin’s *in vivo* distribution is controlled by an intricate interaction between drug transporters and metabolic enzymes, which may also affect the pharmacokinetics of other drugs that depend on the same cytochrome P450 (CYP) enzymes or have a high protein binding affinity (Huang et al., 2019; Zhu et al., 2013). Although certain traditional Chinese medicines (TCMs) have been linked to side effects such as interstitial pneumonia and liver impairment, safety studies have demonstrated that baicalin is well-tolerated. Numerous cell and animal model experiments have established safe dosage levels that do not interfere with normal physiological processes. For instance, Hao et al. (2021) showed that 25–100 mg/kg baicalin effectively inhibited pulmonary inflammation, apoptosis, and alveolar injury in COPD models by activating HSP72 and blocking JNK pathway activation without causing weight loss or liver and kidney toxicity. Oral administration of baicalin at a dose of 100 mg/kg did not alter weight gain or cardiac function in rats. Serum cardiac marker levels (NT-pro BNP, BNP, cTnT, and CK-MB) and inflammatory markers (CRP, LDH, MCP-1, and TNF-α) remained unchanged, and histological analysis revealed normal myocardial morphology without inflammation or tissue damage (Feng et al., 2022; Meng et al., 2025). Baicalin has not been found to exhibit any evident toxicity in numerous animal models, such as allergic asthma and alcoholic fatty liver disease (Meng et al., 2025; Huang et al., 2025; Wang et al., 2020). No significant acute side effects of baicalin have been reported. Chronic toxicity can occur at high doses over long periods; however, it could cause allergic responses or fibrosis in the kidneys (Tang and Zhang, 2022). Baicalin appears to be much safer than other flavonoids, such as quercetin, which has been associated with hepatic stress and genotoxicity, or hesperidin and naringenin, which can damage epithelial barriers (Nakashima et al., 2020; Nakashima et al., 2023) It is also less toxic than its derivative, wogonin, which at higher doses (approximately 120 mg/kg) exhibited cardiotoxic effects and induced increases in body weight in mice (Qi et al., 2009).

BA is an overall promising candidate as an anticancer agent, especially for BC treatment. Its stability, good safety profile, and wide bioactivity indicate it as a rich lead compound of traditional Chinese medicine. However, issues persist over its poor bioavailability and unpredictable pharmacological action. Ongoing developments in formulation and drug delivery technologies are paving the way for improved baicalin formulations with enhanced bioavailability. This results in increased therapeutic activity, positioning baicalin as a more promising candidate for future anticancer treatments.

## 5 Limitations and future directions

Although the current research provides significant insights into the antioxidant, biocompatibility, and anticancer effects of baicalin, some limitations should be considered. The mechanistic understanding of apoptosis and oxidative stress has been limited to morphology and fluorescence-based assays. Molecular-level validation by qPCR or Western blotting of apoptotic markers is essential to ensure the implicated signalling pathways. Second, although BA exhibited significant selectivity toward MCF-7 cells compared to normal PBMCs and fibroblasts, normal mammary epithelial cell lines would be included in future work to make the safety comparison more physiologically relevant. For future perspectives, research will continue to explore nanoformulation strategies to enhance baicalin’s solubility, stability, and cellular uptake, thereby increasing its bioavailability and anticancer activity. Moreover, molecular-level confirmation of the mitochondrial apoptotic pathway and *in vivo* assessment in BC models are proposed to determine the translational significance of BA. Collectively, these studies will lay the groundwork for the development of BA as a safe, effective, and naturally occurring therapeutic agent for the treatment of BC.

## 6 Conclusion

This study introduces BA, a natural compound extracted from *S. baicalensis*, as a selective anticancer agent against breast cancer cells. Through thorough physicochemical characterisation and a series of *in vitro* experiments, BA was revealed to have high cytotoxic activity against MCF-7 cells with minimal toxicity towards healthy cells. Mechanistic explorations revealed that BA induces apoptosis through ROS production, mitochondrial membrane depolarisation, lysosomal destabilisation, and nuclear damage. Additionally, BA potently inhibited cell proliferation, implying its ability to inhibit metastatic development. Computational analyses also evidenced BA’s potent binding affinity with the primary cancer-related targets CA9, DHFR, and MMP1; molecular dynamics simulations validated the stability of these interactions. The study is limited to *in vitro* and *in silico* systems, and further *in vivo* experiments must be conducted to validate the current research findings, which are critical for assessing systemic toxicity, pharmacokinetics, and bioavailability and informing the development of future anticancer drugs. Nanoformulation methods or combinational approaches should be investigated in future research to improve delivery and efficacy. Despite these limitations, the results firmly establish baicalin as a multi-targeted, biocompatible, and mechanistically diverse candidate with therapeutic value in the treatment of breast cancer.

## Data Availability

The original contributions presented in the study are included in the article/Supplementary Material; further inquiries can be directed to the corresponding author.

## Abbreviations

BC, breast cancer; BA, baicalin; BE, baicalein; FT-IR, Fourier-transform infrared spectroscopy; HPTLC, high-performance thin-layer chromatography; HPLC, high-performance liquid chromatography; NMR, nuclear magnetic resonance; SEM, scanning electron microscopy; XRD, X-ray diffraction; RBC, red blood cell; DPPH, 2,2-diphenyl-1-picrylhydrazyl; FRAP, ferric-reducing antioxidant power; RPA, reducing power assay; ABTS, 2,2′-azino-bis(3-ethylbenzothiazoline-6-sulfonic acid; H_2_O_2_, hydrogen peroxide; AIA, anti-inflammatory activity; FBS, fetal bovine serum; DMEM, Dulbecco’s modified Eagle medium; RPMI, Roswell Park Memorial Institute; PBS, phosphate-buffered saline; MTT, 3-(4,5-dimethylthiazol-2-yl)-2,5-diphenyltetrazolium bromide; H_2_DCFDA, 2′,7′-dichlorodihydrofluorescein diacetate; AO, acridine orange; EtBr, ethidium bromide; CV, cell viability; MDS, molecular dynamics simulation.
