# Supplementary material for: Exploring the therapeutic potential of baicalin against MCF-7 breast cancer cells: biochemical, in vitro, and computational perspectives
Source: Front Pharmacol. 2026 Jan 9;16:1698631. doi: 10.3389/fphar.2025.1698631 (PMC12828673; doi:10.3389/fphar.2025.1698631)
Supplement: Supplementary file 1 [file Supplementaryfile1.docx]

Supplementary Material

# **Supplementary Figures and Tables**

## **Supplementary Figures**


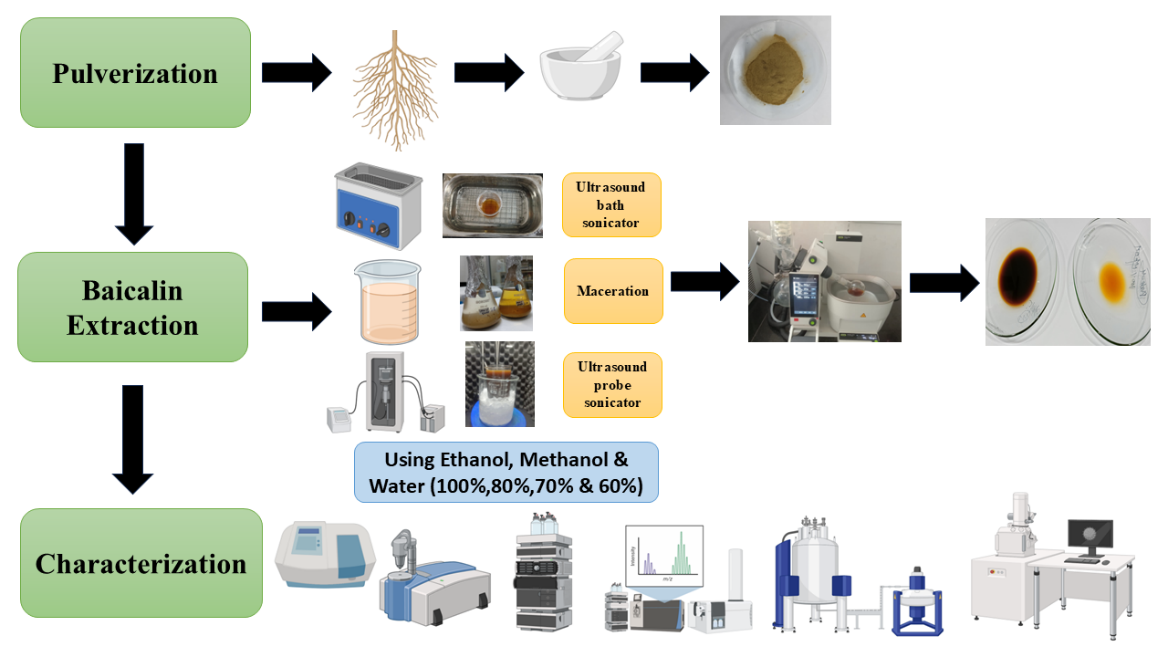


**Figure S1.** Detailed outline of the baicalin extraction process from *Scutellaria baicalensis*.


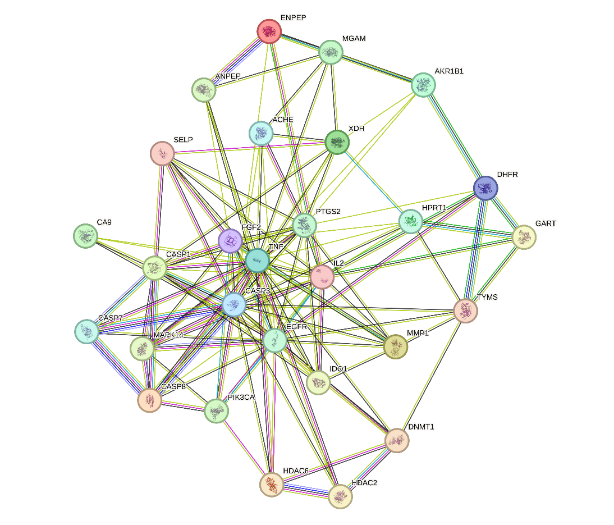


**Figure S2.** PPI network constructed to identify key molecular targets of baicalin against BC The network was generated using STRING.

## **Supplementary Tables**

**Table T1.** Extraction of BA by different methods and solvents.

|  | **60%** | | **70%** | | **80%** | | **100%** | |
| --- | --- | --- | --- | --- | --- | --- | --- | --- |
| **Probe Sonicator** |  | |  | |  | |  | |
| **Solvent** |  |  |  |  |  |  |  |  |
| Methanol | 1.60gm | 32% | 1.3gm | 26% | 1.0 gm | 20% | 0.17gm | 3.4% |
| Ethanol | 1.2gm | 24% | 1.0gm | 20% | 0.90gm | 18% | 0.05gm | 1% |
| Water | ̶ | ̶ | ̶ | ̶ | ̶ | ̶ | 1.05gm | 21% |
| **Bath Sonicator** |  |  |  |  |  |  |  |  |
| Methanol | 1.16gm | 23.2% | 1.15gm | 23% | 0.63gm | 12.6% | 0.10gm | 2% |
| Ethanol | 0.97gm | 19.4% | 0.91gm | 18.2% | 0.60gm | 12% | 0.01gm | 0.2% |
| Water | ̶ | ̶ | ̶ | ̶ | ̶ | ̶ | 0.72gm | 14.4% |
| **Maceration** |  |  |  |  |  |  |  |  |
| Methanol | 0.96gm | 19.2% | 0.90gm | 18% | 0.84gm | 16.8% | 0.08gm | 1.6% |
| Ethanol | 1.03gm | 20.6% |  | 18.4% | 0.66gm | 13.2% | 0.12gm | 2.4% |
| Water | ̶ | ̶ | ̶ | ̶ | ̶ | ̶ | 0.93gm | 18.6% |

**Table T2.** Chemical characterisation (FT-IR) analysis of the standard of BA.

| **S. No.** | **Wave number (cm^-1^) in the standard of BA** | **Wave number (cm^-1^) in SB extract** | **FTIR functional group** | **Type of bond** |
| --- | --- | --- | --- | --- |
| 1. | 3338.85 | 3306.40 | alcohols, phenols, | O-H stretch, |
| 2. | 2944.53 | 2944.69 | alkanes | C-H stretch |
| 3. | 2833.35 | 2834.17 | alkanes | C-H stretch |
| 4. | 1640.91 | 1634.23 | alkenes | C=C stretch |
| 5. | 1408.61 | 1399.94 | carboxylic acids, alkanes | O-H bend, |
| 6. | 1019.48 | 1017.24 | alcohols, ethers, esters | C-O stretch |

**Table T3.** Information about BA**.**

| **Properties** | **Parameters** | **Baicalin** |
| --- | --- | --- |
| Identity information | Molecular Weight | 447.09 |
|  | PubChem CID | 64982 |
|  | Canonical SMILES | C1=CC=C(C=C1)C2=CC(=O)  C3=C(C(=C(C=C3O2)OC4C(C  (C(C(O4)C(=O)O)O)O)O)O)O |
| Physicochemical properties | LogP | 0.1422 |
|  | Number of Rotatable Bonds | 4 |
|  | Number of H-bond Acceptors | 11 |
|  | Number of H-bond Donors | 6 |
|  | Surface Area | 178.474 |
|  | Fraction Csp3 | 0.24 |
|  | Molar Refractivity | 106.72 |
|  | TPSA | 187.12 Å |
| Log P | iLOGP | 1.59 |
|  | XLOGP3 | 1.11 |
|  | MLOGP | -1.63 |
|  | WLOGP | 0.14 |
|  | Consensus | 0.22 |
| Permeation | Water Solubility, Log S (ESOL) | -3.41 Soluble |
|  | GI absorption | Low |
|  | Log Kp (skin permeation) (cm/ s) | -8.23 cm/s |
|  | Intestinal absorption (human) | 39.765 |
| Distribution | P-gp substrate | Yes |
|  | Blood-brain barrier (BBB) permeant | No |
|  | VDss (human) | -1.725 |
|  | CNS permeability | -3.863 |
|  | Fraction unbound (human) | 0.349 |
|  | P-glycoprotein substrate | Yes |
|  | P-glycoprotein I inhibitor | No |
|  | P-glycoprotein II inhibitor | No |
| Metabolism | CYP2D6 substrate | No |
|  | CYP1A2 inhibitor | No |
|  | CYP2C19 inhibitor | No |
|  | CYP2C9 inhibitor | No |
|  | CYP2D6 inhibitor | No |
|  | CYP3A4 inhibitor | No |
| Excretion | Total Clearance | 0.121 |
|  | Renal OCT2 substrate | No |
| Toxicity | AMES toxicity | No |
|  | Max. tolerated dose (human) | 0.449 |
|  | hERG I inhibitor | No |
|  | hERG II inhibitor | No |
|  | Oral Rat Acute Toxicity (LD50) | 1.813 |
|  | Oral Rat Chronic Toxicity (LOAEL) | 2.104 |
|  | Hepatotoxicity | No |
|  | Skin Sensitisation | No |
| Drug likeness | Lipinski violations | No, 2 violations |
|  | Ghose violations | Yes |
|  | Veber violations | No, 1 violation |
|  | Bioavailability Score | 0.11 |
| Medicinal Chemistry | PAINS alert | 1 alert |
|  | Brenk alert | 1 alert |
|  | Lead likeness | No, 1 violation |
|  | Synthetic accessibility | 5.09 |

**Table T4.** Prediction of binding sites utilising CastP.

| **S. No.** | **Name of the protein** | **PDB ID** | **Active site residue** |
| --- | --- | --- | --- |
|  | ACHE | 4M0E | ASN 233, PRO 235, GLU 313, ILE 316, ASN 317, VAL 367, VAL 370, HIS 405, CYS 409, PRO 410, ALA 412, GLN 413, GLY 416, ARG 417, TYR 503, ALA 505, GLY 506, GLN 508, LEU 524, ALA 526, CYS 529, ALA 530, TRP 532, ASN 533, ARG 534, LEU 536, PRO 537, LYS 538, LEU 540, TRP 385, PRO 388 |
|  | AKR1B1 | 6F7R | THR 19, TRP 20, LYS 21, ASP 43, VAL 47, TYR 48, LYS 77, TRP 79, HIS 110, TRP 111, PHE 122, SER 159, ASN 160, GLN 183, TYR 209, SER 210, PRO 211, LEU 212, SER 214, PRO 215, ASP 216, TRP 219, LEU 228, ALA 245, ILE 260, PRO 261, LYS 262, SER 263, VAL 264, THR 265, ARG 268, GLU 271, ASN 272, VAL 297, CYS 298, ALA 299, LEU 300, LEU 301 |
|  | CA9 | 6Y74 | GLN 138, SER 139, HIS 140, TRP 141, TYR 143, VAL 152, SER 153, PRO 154, ALA 155, PRO 191, GLU 192, LEU 193, ARG 194, ARG 196, ASN 198, GLY 199, HIS 200, SER 201, GLN 203, THR 205, LEU 206, PRO 207, PRO 208, LEU 223, GLN 224, HIS 226, HIS 228, HIS 251, VAL 253, ALA 260, ARG 261, VAL 262, ASP 263, GLU 264, LEU 266, GLY 267, ARG 268, LEU 272, VAL 274, GLU 302, GLU 305, GLN 307, LEU 331, THR 332, THR 333, PRO 334, PRO 335, ALA 337, TRP 342, PRO 366 |
|  | CASP3 | 6CKZ | SER 29, GLY 30, ILE 31, SER 32, LEU 33, ASP 34, ASN 35, SER 36, TYR 37, LYS 38, MET 39, ASP 40, LYS 271, GLU 272, TYR 274, TYR 276, HIS 277, LEU 278 |
|  | CASP1 | 6BZ9 | ILE 176, PRO 177, ARG 178, HIS 237, GLY 238, ILE 239, CYS 244, HIS 248, CYS 285, GLY 287, ASP 288, VAL 338, ARG 341 |
|  | CASP7 | 6CL1 | GLU 146, GLU 147, ASN 148, VAL 149, ILE 159, LYS 160, ASP 161, THR 163, ALA 164, ARG 167, ARG 170, ILE 183, ALA 185, CYS 186, ARG 187, GLU 190, GLU 216, PHE 219, PHE 221, TYR 223, SER 224, THR 225, VAL 226, PRO 227, TYR 229, CYS 290, VAL 292, MET 294 |
|  | CASP8 | 3KJQ | LEU 254, SER 256, ILE 257, ARG 258, ASP 259, ARG 260, ASN 261, SER 316, HIS 317, GLY 318, ASP 319, TYR 324, GLN 358, ALA 359, CYS 360, GLY 362, ASP 363, TYR 365, VAL 410, SER 411, TYR 412, ARG 413, PRO 415, ASP 455, ASN 458 |
|  | DHFR | 1RF7 | ILE 5, ALA 6, ALA 7, ILE 14, GLY 15, MET 16, GLU 17, ASN 18, MET 20, TRP 22, ASN 23, LEU 24, PRO 25, ASP 27, LEU 28, ALA 29, TRP 30, PHE 31, LYS 32, GLY 43, ARG 44, HIS 45, THR 46, SER 49, ILE 50, ARG 52, LEU 54, PRO 55, ARG 57, LEU 62, SER 63, SER 64, LYS 76, SER 77, VAL 78, ILE 94, GLY 95, GLY 96, GLY 97, ARG 98, VAL 99, TYR 100, GLN 102, TYR 111, LEU 112, THR 113, THR 123 |
|  | FGF2 | 8OM6 | GLU 128, GLU 129, ASN 130, THR 134, PRO 161, GLY 162, GLN 163, ILE 166, LEU 167 |
|  | GART | 1MEN | ILE 7, SER 8, GLY 9, THR 10, GLY 11, SER 12, ASN 13, GLN 15, ALA 16, ILE 18, ASP 19, ARG 22, GLU 23, PRO 24, ASN 36, LYS 37, ALA 38, ALA 39, VAL 40, ALA 41, ASP 44, LYS 45, ARG 48, ALA 49, HIS 58, LYS 59, TYR 61, LYS 62, ASN 63, ARG 64, PHE 67, LEU 85, ALA 86, GLY 87, PHE 88, MET 89, ARG 90, ILE 91, LEU 92, GLY 94, VAL 97, ASN 106, ILE 107, HIS 108, PRO 109, LEU 112, PRO 113, LYS 116, GLY 117, SER 118, ASN 119, ALA 120, HIS 121, GLU 122, GLN 123, LEU 125, GLU 126, HIS 137, VAL 139, ALA 140, GLU 141, VAL 143, ASP 144, GLY 146, ILE 148, LYS 157, GLY 159, ASP 160, THR 161, VAL 162, ALA 163, THR 164, SER 166, GLU 167, ARG 168, LYS 170, LEU 171, GLU 173, HIS 174, ARG 0, SER 20, THR 21, ASN 25, ALA 28, GLN 29, ARG 158, LYS 175, PRO 178, ALA 179, LEU 14, ARG 54, VAL 55, ILE 56, ASN 57, LEU 60, GLU 66, SER 69, ALA 70, LEU 73, VAL 74, GLU 77 |
|  | HDAC2 | 7LTG | LEU 212, ARG 213, ILE 215, LYS 221, TYR 222, ASP 235, GLU 236, SER 237, TYR 238, GLY 239, GLN 240, PHE 242, LYS 243, PRO 244, SER 247, LYS 248, GLU 251, GLY 281, LYS 284, CYS 285, GLU 287, VAL 288, THR 291, PHE 292, GLU 324, ASN 355, PRO 357, GLU 358, MET 360, GLU 361, LYS 362, LYS 364, GLN 365, ARG 366, PHE 368, GLU 369, ASN 370, ARG 372, MET 373, LEU 44, ASN 45, TYR 46, GLY 47, TYR 49, ARG 50, GLY 232, ASP 234, SER 268, GLY 269, ASN 276, THR 278, VAL 279, LYS 280, THR 305, ARG 307, ASN 308, ARG 311, TYR 315, ILE 325, PRO 326, ASN 327, GLU 328, LEU 329, PRO 330, TYR 331, GLY 339, PRO 340, ASP 341, PHE 342, LYS 343, HIS 345, ILE 346, SER 347, PRO 348, SER 349, ASN 350, MET 351, ASN 353, HIS 58, LYS 59, ALA 60, THR 61, ALA 62, GLU 63, GLU 64, THR 66, LYS 67, TYR 68, SER 70, ASP 71, GLU 72, ILE 74, LYS 75, ARG 78, GLN 91, ARG 94, PHE 95, VAL 119, ALA 120, VAL 123, ASN 126, ARG 127, LYS 144, LYS 145, SER 146, GLU 147, SER 149, VAL 158, LEU 159, LEU 162, GLU 163, LEU 165, LYS 166, TYR 167, HIS 180, ASP 182, GLU 185, GLU 186, ALA 187, PHE 188, TYR 189, THR 190, THR 191, ARG 193, TYR 202, PHE 206, PRO 207, GLY 208, THR 209, GLY 210, ASP 211, ASP 214, GLY 216, ALA 217, GLY 218 |
|  | MGAM | 2QLY | GLU 114, HIS 115, LEU 278, GLU 279, GLY 282, ARG 283, PRO 284, ALA 285, LEU 286, PRO 287, SER 288, ALA 291, VAL 506, ALA 509, GLU 510, ALA 512, LYS 513, ARG 520, SER 521, PHE 522, ILE 523, GLY 533, LYS 534, PHE 535, ALA 536, ALA 537, ILE 565, PRO 566, MET 567, PHE 641, HIS 645, SER 646, THR 775, LYS 776, ASP 777, THR 778, VAL 779, ALA 780 |
|  | MMP1 | 3SHI | PRO 123, ASP 124, ARG 165, PRO 177, GLY 178, GLU 199, ASP 200, GLU 201, ARG 202, GLU 209, TYR 210, GLN 139, SER 142, ASN 143, THR 145, PRO 146, LEU 147, THR 148 |
|  | MAPK14 | 5LAR | VAL 30, GLY 33, ALA 34, TYR 35, GLY 36, VAL 38, ALA 51, LYS 53, LEU 55, SER 56, PRO 58, HIS 64, ARG 67, THR 68, ARG 70, GLU 71, LEU 74, LEU 75, MET 78, VAL 83, ILE 84, LEU 104, THR 106, HIS 107, LEU 108, MET 109, GLY 110, ALA 111, ASP 112, ILE 141, ASP 145, ILE 146, ILE 147, HIS 148, ARG 149, ASP 150, LYS 152, SER 154, ASN 155, ALA 157, ILE 166, LEU 167, ASP 168, PHE 169, GLY 170, LEU 171, ALA 172, ARG 173, HIS 174, GLU 178, GLY 181, TYR 182, VAL 183, ALA 184, TYR 323, GLN 325, PHE 327, GLU 328 |
|  | PTGS2 | 5F1A | ASN 34, CYS 36, HIS 39, PRO 40, CYS 41, GLN 42, ASN 43, ARG 44, GLY 45, VAL 46, CYS 47, MET 48, SER 49, THR 60, ARG 61, THR 62, GLY 63, PHE 64, GLU 73, LEU 75, THR 76, LYS 79, LEU 80, LYS 83, PRO 84, THR 85, PRO 86, ASN 87, VAL 89, HIS 90, TYR 91, LEU 93, THR 94, HIS 95, MET 113, TYR 115, VAL 116, THR 118, SER 119, ARG 120, SER 121, HIS 122, LEU 123, ILE 124, ASP 125, SER 126, PRO 127, PRO 128, THR 129, TYR 130, ALA 132, ASP 133, TYR 134, GLY 135, TYR 136, LYS 137, TRP 139, GLU 140, PHE 142, SER 143, LEU 145, THR 149, ARG 150, ALA 151, LEU 152, PRO 153, PRO 154, VAL 155, PRO 156, ASP 157, GLN 192, PHE 205, THR 206, PHE 209, LEU 224, GLY 225, HIS 226, GLY 227, VAL 228, ASP 229, ASN 231, GLY 235, GLU 236, THR 237, LEU 238, GLN 241, GLU 322, TRP 323, GLY 324, GLU 326, GLN 327, PHE 329, GLN 330, THR 331, ARG 333, LEU 334, VAL 344, ILE 345, TYR 348, VAL 349, LEU 352, SER 353, GLY 354, TYR 355, LEU 359, LEU 366, PHE 367, LYS 369, GLN 370, PHE 371, GLN 372, TYR 373, GLN 374, ASN 375, ARG 376, ILE 377, ALA 378, PHE 381, LEU 384, TYR 385, TRP 387, LYS 459, GLN 461, GLU 465, LYS 468, ARG 469, PHE 470, MET 471, LEU 472, LYS 473, ARG 513, PRO 514, ASP 515, ALA 516, ILE 517, PHE 518, MET 522, VAL 523, GLU 524, GLY 526, ALA 527, PHE 529, SER 530, LEU 531, LYS 532, GLY 533, LEU 534, GLY 536, ASN 537, VAL 538, SER 541, PRO 542, ALA 543, LYS 546, SER 548, THR 549, GLY 551, LYS 33, PRO 35, GLY 51, PHE 52, ASP 158, ASN 368, ARG 467, TYR 475, GLU 480, GLU 510, LYS 511, GLU 520 |
|  | TYMS | 5X66 | LYS 47, ASP 48, ASP 49, ARG 50, THR 51, THR 55, LYS 77, ARG 78, VAL 79, PHE 80, GLU 87, LEU 88, PHE 91, ILE 108, TRP 109, ALA 111, ASN 112, SER 114, ASP 116, PHE 117, ASP 119, SER 120, LEU 121, GLY 122, PHE 123, SER 124, VAL 134, TYR 135, TRP 139, TRP 182, PRO 184, LEU 187, PRO 188, LEU 189, MET 190, ALA 191, LEU 192, PRO 193, PRO 194, CYS 195, HIS 196, GLN 214, ARG 215, SER 216, GLY 217, ASP 218, GLY 220, LEU 221, GLY 222, PRO 224, PHE 225, ASN 226, SER 229, TYR 230, LEU 233, ASP 254, HIS 256, TYR 258, MET 311, ALA 312, VAL 313, LEU 67, ARG 68, ASP 69, GLU 70, LEU 73, TRP 81, LYS 82, LEU 85, GLU 86, GLU 207, TYR 235, ALA 238, HIS 239, GLY 242, LEU 243, LYS 244, PRO 245, ARG 274, PRO 275, PHE 276, PRO 277, LYS 278, LEU 279, ARG 280, ILE 281, LEU 282, ARG 283, LYS 284, ALA 293, GLU 294, PHE 296, GLN 297, ILE 298, GLU 299, GLY 300, TYR 301, ASN 302, PRO 303, HIS 304, PRO 305, VAL 158, GLN 160, GLN 162, ARG 163, VAL 164, ILE 165, ASP 166, THR 167, LYS 169, THR 170, ASN 171, ASP 173, ASP 174, ARG 175, ARG 176, ILE 177, ILE 178, ILE 240, GLU 286, LYS 287, ILE 288 |
|  | ENPEP | 4FYQ | TYR 74, PHE 103, SER 124, LYS 125, LYS 126, LEU 127, ASN 128, LEU 155, VAL 156, GLU 157, PRO 158, GLU 160, TYR 161, GLY 184, GLU 185, ALA 187, ASP 188, ASP 189, LEU 190, ALA 191, PHE 193, TYR 194, ARG 195, SER 196, GLU 197, TYR 198, MET 199, GLU 200, GLY 201, ARG 204, GLN 211, GLN 213, ALA 214, ALA 215, ASP 216, LYS 219, ARG 305, PRO 306, SER 307, ALA 308, ALA 311, HIS 313, LEU 346, PRO 347, ASP 348, PHE 349, ASN 350, ALA 351, GLY 352, ALA 353, MET 354, GLU 355, ARG 363, GLU 364, ASN 365, LEU 368, SER 375, SER 376, SER 377, ASN 378, GLU 380, ARG 381, THR 384, VAL 385, HIS 388, GLU 389, HIS 392, GLU 411, SER 415, GLU 418, TYR 419, LEU 434, LEU 437, ASN 438, ASP 439, TYR 441, ARG 442, ALA 445, VAL 446, ALA 448, LEU 449, SER 452, HIS 453, THR 457, ILE 462, THR 464, PRO 465, ALA 466, GLN 467, ILE 468, SER 469, GLU 470, LEU 471, PHE 472, ASP 473, ALA 474, ILE 475, TYR 477, SER 478, LEU 567, LEU 568, ASP 569, PRO 570, ASP 571, SER 572, ARG 631, ASN 663, PHE 666, ASN 667, ALA 669, SER 670, HIS 672, VAL 674, PRO 675, VAL 676, TYR 692, GLU 696, ALA 697, LEU 699, SER 700, SER 701, LEU 702, SER 703, TYR 704, PHE 705, LYS 706, LEU 707, ASP 710, TYR 715, MET 718, LYS 719, LEU 722, GLN 750, TYR 751, VAL 754, ASN 755, SER 758, THR 759, CYS 761, SER 762, ASN 763, GLY 764, GLU 769, PRO 790, ASN 791, SER 794, THR 795, CYS 798, ASN 799, ALA 802, GLN 803, PHE 816, ARG 817, ASN 818, ALA 819, THR 820, LEU 821, VAL 822, ASN 823, ALA 825, ASP 826, LYS 827, ARG 829, ALA 830, ALA 831, ALA 833, CYS 834, LYS 836, THR 848, LEU 849, ASN 850, PRO 851, ASP 852, LEU 853, ILE 854, ARG 855, LYS 856, GLN 857, ASP 858, ALA 859, THR 860, SER 861, THR 862, ILE 863, ILE 864, SER 865, ASN 868, ASN 869, VAL 870, ILE 871, TRP 884, LYS 886, LEU 887, PHE 888, ASN 889, ASP 890, SER 899, ASN 900, LEU 901, ILE 902, GLN 903, ALA 904, THR 906, ARG 907, ARG 908, THR 928, GLY 929, PHE 930, GLY 931, SER 932, GLY 933, ARG 935, ALA 936, GLN 939, ALA 940, GLU 942, LYS 943, THR 944, ALA 946, ASN 947, LYS 949, TRP 950, GLU 953, ASN 954 |
|  | DNMT1 | 4WXX | VAL 307, ILE 311, GLU 314, LYS 315, LYS 406, LEU 407, THR 408, CYS 409, PHE 410, SER 411, VAL 412, TYR 413, CYS 414, LYS 415, HIS 416, LEU 419, CYS 420, PRO 421, THR 424, TYR 443, ASP 445, ASP 446, SER 448, GLU 450, GLY 472, GLU 473, LYS 474, PRO 497, PHE 499, GLY 500, MET 502, GLU 504, LYS 505, ILE 506, TYR 507, LYS 510, ASN 519, SER 522, ILE 531, THR 534, VAL 535, PRO 536, PRO 537, SER 538, GLY 539, LEU 540, ASN 541, LEU 542, ARG 544, PHE 545, THR 546, GLU 547, ASP 548, VAL 557, VAL 558, GLU 559, GLN 560, VAL 561, GLU 562, SER 563, TYR 564, ASP 565, GLU 566, ALA 567, GLY 568, ASP 569, SER 570, ASP 571, GLU 572, GLN 573, PRO 574, MET 581, ARG 582, ASP 583, ILE 585, ALA 588, THR 591, GLY 593, ARG 596, ALA 597, GLN 598, ALA 599, ARG 600, ARG 601, ARG 609, LYS 611, ASP 612, ARG 613, GLY 614, PRO 615, THR 616, ALA 618, THR 620, THR 621, LYS 622, LEU 623, GLN 626, PHE 628, ASP 629, PHE 631, PHE 632, GLU 634, GLN 635, ILE 636, GLU 637, LYS 638, ASP 639, ASP 640, ARG 641, GLU 642, ASP 643, LYS 644, ASN 646, ALA 647, LYS 649, ARG 650, ARG 651, ARG 652, VAL 655, CYS 656, PRO 662, GLU 663, CYS 664, CYS 667, LYS 668, ALA 669, CYS 670, LYS 671, MET 673, VAL 674, LYS 675, PHE 676, GLY 677, GLY 678, SER 679, GLY 680, ARG 681, ARG 690, CYS 691, PRO 692, ASN 693, MET 694, ALA 695, MET 696, LYS 697, GLU 698, ALA 699, GLU 703, GLU 704, VAL 705, ASP 706, ASP 707, ASN 708, ILE 709, PRO 710, GLU 711, MET 712, PRO 713, SER 714, PRO 715, LYS 716, LYS 717, MET 718, GLN 720, GLY 721, LYS 722, LYS 723, LYS 724, LYS 725, GLN 726, ASN 727, LYS 728, VAL 734, GLY 735, GLU 736, LYS 749, CYS 751, ASP 753, ALA 754, GLU 755, THR 756, GLU 758, VAL 759, GLY 760, ASP 761, CYS 762, LYS 831, SER 840, GLU 841, ASN 842, TRP 843, GLY 847, GLY 848, MET 849, ASP 850, GLU 852, LEU 854, GLU 856, GLY 857, ASP 858, LYS 951, LEU 952, SER 953, ARG 958, PRO 959, ARG 960, LYS 961, GLU 962, PRO 963, VAL 964, ASP 967, LEU 968, ASN 984, LEU 985, ASP 986, ALA 987, GLU 989, PRO 990, TYR 991, ARG 992, ILE 993, GLY 994, ARG 995, ILE 996, LYS 997, GLU 998, HIS 1028, LYS 1029, SER 1030, THR 1031, PRO 1032, ALA 1033, SER 1034, TYR 1035, HIS 1036, ALA 1037, ASP 1038, ILE 1039, ASN 1040, LEU 1042, TYR 1043, TRP 1044, SER 1045, ASP 1046, GLU 1047, GLU 1048, VAL 1050, VAL 1062, GLU 1063, TYR 1064, GLY 1065, GLU 1066, ASP 1067, GLU 1070, CYS 1071, VAL 1072, GLN 1073, VAL 1074, TYR 1075, SER 1076, MET 1077, ARG 1082, PHE 1085, LEU 1086, GLU 1087, ALA 1088, ASN 1090, ALA 1091, GLU 1129, PRO 1130, GLU 1131, ILE 1132, GLU 1133, ARG 1140, PHE 1187, THR 1188, GLU 1189, ARG 1259, LEU 1282, ARG 1285, VAL 1288, ARG 1289, MET 1290, GLY 1291, GLN 1293, TYR 1304, GLY 1305, PRO 1320, GLY 1321, GLU 1322, LYS 1323, LEU 1324, LEU 1326, PRO 1328, GLU 1329, LEU 1331, HIS 1332, SER 1352, ASN 1353, THR 1355, PHE 1362, ILE 1365, TRP 1395, PHE 1396, ARG 1398, GLN 1399, LEU 1400, ARG 1401, GLY 1402, ALA 1403, GLN 1404, TYR 1405, GLN 1406, PRO 1407, ILE 1408, ARG 1410, GLY 1558, PRO 1560, THR 1562, TYR 1563, ARG 1564, PRO 1583, LYS 1586, ALA 1587, LEU 1590, GLU 1591, LYS 1593, LEU 1594, CYS 1595, LEU 1597, ALA 1598, LYS 1599, ALA 1600, ARG 1601, GLU 1602, SER 1603, SER 1605, ALA 1606, LYS 1607, ILE 1608, LYS 1609, GLU 1610, GLU 1611, GLU 1612, ALA 1613, ALA 1614, LYS 1615, ASP 1616 |
|  | EGFR | 4WKQ | ARG 2, SER 4, GLY 5, THR 6, ALA 7, GLY 8, ALA 9, ALA 10, LEU 11, LEU 12, ALA 13, LEU 14, LEU 15, ALA 16, ALA 17, LEU 18, CYS 19, PRO 20, ALA 21, SER 22, ARG 23, ALA 24, LEU 25, GLU 26, GLU 27, LYS 28, LYS 29, VAL 30, CYS 31, GLN 32, GLY 33, THR 34, SER 35, ASN 36, LYS 37, LEU 38, THR 39, LEU 41, GLY 42, ASP 46, HIS 47, SER 50, LEU 51, ARG 53, MET 54, ASN 56, ASN 57, CYS 58, GLU 59, VAL 60, LEU 62, GLY 63, ASN 64, GLU 66, THR 68, GLU 84, ALA 86, GLY 87, TYR 88, ARG 108, GLY 109, ASN 110, MET 111, TYR 112, GLU 114, ASN 115, GLU 142, LEU 144, HIS 145, SER 169, SER 170, ASP 171, PHE 172, ILE 213, ILE 214, CYS 215, ALA 216, ARG 222, CYS 223, ARG 224, GLY 225, LYS 226, SER 227, SER 229, ASN 234, CYS 236, ALA 237, ALA 238, GLY 239, LYS 284, TYR 285, SER 286, LYS 294, PRO 296, HIS 304, GLY 305, CYS 307, VAL 308, ASP 321, GLY 322, VAL 323, ARG 324, LYS 325, CYS 326, LYS 327, LYS 328, CYS 329, GLU 330, GLY 331, PRO 332, CYS 333, ARG 334, ILE 340, GLY 341, ILE 342, GLY 343, GLU 344, PHE 345, LYS 346, SER 348, LEU 349, SER 350, ILE 351, LYS 360, SER 364, ILE 365, SER 366, GLY 367, ASP 368, LEU 369, HIS 370, ILE 371, LEU 372, VAL 374, ALA 375, PHE 376, ARG 377, GLY 378, ASP 379, SER 380, PHE 381, THR 382, HIS 383, LEU 387, ASP 388, PRO 389, GLN 390, GLU 391, LEU 392, ASP 393, ILE 394, LEU 395, LYS 396, THR 397, VAL 398, LYS 399, GLU 400, ILE 401, THR 402, PHE 404, LEU 406, ILE 407, GLU 412, ASN 413, ARG 414, THR 415, ASP 416, LEU 417, HIS 418, LEU 423, GLU 424, ILE 425, ILE 426, GLY 428, ARG 429, THR 430, LYS 431, GLN 432, HIS 433, GLY 434, GLN 435, PHE 436, SER 437, LEU 438, ALA 439, VAL 440, VAL 441, THR 446, LEU 448, GLY 449, LEU 450, ARG 451, SER 452, LEU 453, LYS 454, GLU 455, ILE 456, SER 457, ASP 458, GLY 459, ASP 460, ILE 462, ILE 463, SER 464, GLY 465, ASN 466, ASN 468, TYR 471, LEU 480, PHE 481, GLY 482, GLN 486, LYS 487, THR 488, LYS 489, ILE 491, ASN 493, ARG 494, GLY 495, GLU 496, ASN 497, SER 498, CYS 499, LYS 500, ALA 501, THR 502, GLY 503, GLN 504, VAL 505, CYS 506, PRO 512, GLU 513, GLY 514, CYS 515, TRP 516, GLY 517, PRO 518, GLU 519, PRO 520, ARG 521, ASP 522, CYS 523, VAL 524, SER 525, CYS 526, ARG 527, ASN 528, VAL 529, SER 530, ARG 531, LYS 538, CYS 539, ASN 540, LEU 541, LEU 542, GLU 543, GLY 544, GLU 545, PRO 546, ARG 547, VAL 550, GLU 551, ASN 552, SER 553, GLU 554, CYS 555, GLN 557, MET 567, ARG 574, GLY 575, PRO 576, ASP 577, HIS 590, CYS 591, VAL 592, LYS 593, THR 594, ALA 597, GLY 598, VAL 599, MET 600, GLY 601, ASN 603, PRO 631, GLY 632, LEU 633, GLY 640, PRO 641, LYS 642, ILE 643, PRO 644, SER 645, ILE 646, ALA 647, ALA 653, LEU 655, LEU 656, LEU 657, LEU 658, VAL 659, VAL 660, ILE 664, ARG 671, GLU 690, PRO 691, LEU 692, THR 693, PRO 694, SER 695, LYS 714, ILE 715, LYS 716, VAL 717, LEU 718, GLY 719, SER 720, GLY 721, ALA 722, PHE 723, GLY 724, THR 725, VAL 726, LYS 728, VAL 738, LYS 739, PRO 741, VAL 742, ALA 743, ILE 744, LYS 745, GLU 746, ARG 748, LEU 760, ASP 761, GLU 762, ALA 763, VAL 765, ALA 767, LEU 782, THR 783, SER 784, THR 785, VAL 786, GLN 787, LEU 788, ILE 789, THR 790, GLN 791, LEU 792, MET 793, PRO 794, PHE 795, GLY 796, CYS 797, LEU 798, LEU 799, ASP 800, GLU 804, LYS 806, ILE 809, GLY 810, SER 811, GLN 812, TYR 813, LEU 814, LEU 815, ASN 816, TRP 817, CYS 818, VAL 819, GLN 820, ILE 821, ALA 822, GLY 824, MET 825, ASN 826, TYR 827, LEU 828, GLU 829, ASP 830, ARG 831, ARG 832, LEU 833, VAL 834, HIS 835, ARG 836, ASP 837, LEU 838, ALA 839, ALA 840, ARG 841, ASN 842, VAL 843, LEU 844, VAL 845, LYS 846, THR 847, PRO 848, GLN 849, HIS 850, VAL 851, LYS 852, ILE 853, THR 854, PHE 856, LEU 858, ALA 859, LYS 860, LYS 875, VAL 876, ILE 878, LYS 879, TRP 880, MET 881, ALA 882, LEU 883, GLU 884, SER 885, ILE 886, LEU 887, THR 940, ILE 941, ASP 942, VAL 943, ILE 946, ILE 981, GLN 982, GLY 983, ASP 984, GLU 985, ARG 986, MET 987, LEU 989, SER 991, PRO 992, THR 993, ASP 994, SER 995, PHE 997, TYR 998, ALA 1000, LEU 1001, GLU 1005, ASP 1006, MET 1007, ARG 1052, ASN 1053, GLY 1054, LEU 1055, GLN 1056, SER 1057, CYS 1058, PRO 1059, ILE 1060, GLU 1062, LEU 1066, GLN 1067, ARG 1068, TYR 1069, SER 1070, SER 1071, PRO 1073, THR 1074, ASP 1080, ILE 1082, ASP 1084, ARG 1121, ASP 1122, PRO 1123, HIS 1124, GLU 1137, TYR 1138, LEU 1139 |
|  | HDAC6 | 3PHD | ARG 506, ILE 507, LEU 508, GLY 521, ARG 522, CYS 523, LEU 524, THR 527, PRO 528, ARG 529, PRO 530, ALA 531, GLU 533, ALA 534, GLU 535, LEU 536, THR 538, CYS 539, GLU 552, LYS 553, THR 556, GLU 591, ALA 592, VAL 593, LEU 594, SER 595, GLU 597, VAL 598, LEU 599, ASN 600, ALA 603, VAL 604, VAL 605, ARG 606, PRO 607, PRO 608, GLY 609, HIS 610, HIS 611, ALA 612, GLU 613, GLN 614, ARG 631, HIS 632, GLN 634, THR 635, SER 637, GLY 638, HIS 639, ALA 640, LEU 641, ARG 642, ILE 643, LEU 644, ILE 645, VAL 646, ASP 647, TRP 648, ASP 649, VAL 650, HIS 651, HIS 652, GLY 653, ASN 654, GLY 655, THR 656, GLN 657, HIS 658, MET 659, PHE 660, ASP 663, VAL 666, LEU 667, TYR 668, MET 682, ALA 694, ALA 695, GLY 696, THR 697, GLY 698, PHE 699, THR 700, VAL 701, ASN 702, ALA 704, TRP 705, ASN 706, GLY 707, PRO 708, ARG 709, MET 710, GLY 711, ASP 712, ALA 713, TYR 715, LEU 716, ALA 717, TRP 719, LEU 722, LEU 724, PRO 725, ILE 726, ALA 727, TYR 728, GLU 729, PHE 730, ASN 731, PRO 732, LEU 734, VAL 735, SER 738, PHE 741, ASP 742, ARG 745, ASP 747, PRO 748, GLY 750, GLY 751, CYS 752, GLN 753, VAL 754, SER 755, PRO 756, GLU 757, GLY 758, TYR 759, ALA 760, HIS 761, LEU 762, MET 767, SER 790, ALA 792, LEU 817, ILE 820, THR 821, GLU 822, ILE 824, GLN 825, VAL 826, ARG 866 |
|  | HPRT1 | 1Z7G | VAL 67, LYS 69, GLY 70, GLY 71, LEU 102, LYS 103, SER 104, TYR 105, CYS 106, ASN 107, ASP 108, SER 110, GLU 134, ASP 135, ILE 136, ILE 137, ASP 138, THR 139, GLY 140, LYS 141, THR 142, LYS 166, ARG 170, PHE 187, VAL 188, LEU 193, ASP 194, TYR 195, ASN 196, GLU 197 |
|  | IDO-4 | 2D0T | SER 115, LYS 116, LYS 117, GLU 119, LEU 120, PRO 121, PRO 122, TYR 126, CYS 129, VAL 130, PHE 163, PHE 164, VAL 166, SER 167, VAL 170, GLU 171, PHE 185, PHE 214, ILE 217, VAL 221, PHE 226, PHE 227, ARG 231, LEU 234, SER 235, GLY 236, LYS 238, GLY 239, ASN 240, PRO 241, PHE 252, TRP 253, GLU 254, ASP 255, LYS 257, GLU 258, PHE 259, ALA 260, GLY 261, GLY 262, SER 263, ALA 264, GLY 265, SER 267, PHE 270, GLN 271, ASP 274, VAL 275, LEU 276, GLY 278, ILE 279, GLN 280, GLN 281, THR 282, ALA 283, GLY 284, GLY 285, GLY 286, HIS 287, ALA 288, ALA 289, GLN 290, PHE 291, LEU 292, GLN 293, ASP 294, MET 295, ARG 296, ARG 297, TYR 298, PRO 301, ARG 304, ASN 305, LEU 307, CYS 308, LEU 310, GLU 311, SER 312, ASN 313, PRO 314, SER 315, VAL 316, ARG 317, GLU 318, LEU 321, LEU 342, ARG 343, HIS 346, LEU 347, ILE 349, VAL 350, TYR 353, ILE 354, LEU 355, ALA 358, GLY 380, GLY 381, ASP 383, LEU 384, ASN 386, PHE 387, LEU 388, THR 390, VAL 391, ARG 392, SER 393, THR 394, GLU 396, LYS 397, SER 398, LEU 399, LEU 400, LYS 401, GLU 402, GLY 403, LYS 13, GLU 14, TYR 15, HIS 16, ASP 18, GLY 22, PHE 23, ALA 24, LEU 25, PRO 26, ILE 67, ASP 68, HIS 73, ARG 77, TRP 92, GLY 95, GLY 97, ASP 98, VAL 99, GLN 113, LEU 118, ILE 232, GLN 242, ASP 245, PRO 256, PRO 300, ALA 302, HIS 303 |
|  | IL2 | 1M47 | MET 1, LEU 7, ILE 10, ALA 11, LEU 12, LEU 16, THR 18, ALA 21, PRO 22, THR 23, THR 27, LYS 28, GLN 31, GLU 35, LEU 152, THR 153 |
|  | XDH | 2W3S | MET 1, ILE 3, ILE 16, THR 33, GLY 34, LYS 36, GLY 42, ASP 43, CYS 44, GLY 45, ALA 46, THR 48, ILE 51, ASP 53, ALA 55, ARG 58, ALA 59, VAL 60, ASN 61, LEU 64, MET 65, MET 66, PRO 68, GLN 69, ILE 70, ALA 71, LYS 73, ILE 78, LEU 88, VAL 91, GLN 92, MET 95, ILE 96, HIS 98, HIS 99, GLY 100, SER 101, GLN 102, CYS 103, GLY 104, PHE 105, CYS 106, THR 107, PRO 108, GLY 109, PHE 110, ILE 111, SER 113, MET 114, ALA 116, ALA 117, HIS 118, ARG 120, ARG 122, LYS 123, ASP 124, TYR 125, ASP 126, ASP 127, LEU 128, ARG 144, ALA 145, ALA 146, ALA 148, ALA 149, ALA 150, PRO 154, ALA 155, LEU 158, GLN 159, ALA 160, ASP 161, ALA 162, ALA 163, PHE 164, THR 165, LEU 166, ALA 167, GLN 168, SER 170, SER 171, GLY 172, VAL 173, ARG 174, GLY 175, GLN 176, THR 177, ALA 178, PRO 179, ALA 180, TYR 193, LEU 194, ALA 195, HIS 196, PRO 197, VAL 208, SER 209, LEU 210, GLN 233, GLU 236, THR 237, PRO 238, GLY 240, TYR 241, GLY 244, THR 248, ILE 249, ALA 250, ALA 251, LEU 252, ARG 253, ALA 254, PHE 255, ALA 256, GLU 257, GLY 258, PRO 259, HIS 260, ALA 262, LEU 263, ALA 264, GLY 265, LEU 266, LEU 267, ARG 268, ARG 269, PHE 270, ALA 271, SER 272, GLU 273, GLN 274, VAL 275, ARG 276, GLN 277, VAL 278, ALA 279, THR 280, GLY 283, ASN 284, ILE 285, PRO 290, ILE 291, GLY 292, ASP 293, GLY 294, PRO 295, PRO 296, ALA 297, LEU 298, ILE 299, ALA 300, MET 301, ALA 303, THR 306, ARG 308, ARG 309, GLY 310, GLN 311, GLU 312, ARG 313, ARG 315, LEU 318, GLU 319, ASP 320, PHE 321, PHE 322, LEU 323, GLU 324, TYR 325, ARG 326, LYS 327, GLN 328, ASP 329, ARG 330, ARG 331, PRO 332, GLY 333, GLU 337, SER 338, VAL 339, THR 340, LEU 341, PRO 342, LYS 343, SER 344, ALA 345, PRO 346, GLY 347, LEU 348, ARG 349, CYS 350, TYR 351, LYS 352, THR 371, LEU 372, LYS 373, GLY 374, SER 375, LYS 376, ILE 377, GLU 378, THR 379, ALA 380, ARG 381, ILE 382, ALA 383, PHE 384, GLY 385, GLY 386, MET 387, ALA 388, GLY 389, VAL 390, PRO 391, LYS 392, ARG 393, ALA 394, PHE 397, GLU 398, PHE 406, ARG 407, GLU 408, THR 410, ILE 411, ALA 412, ALA 413, ALA 414, LEU 415, LEU 418, ALA 419, GLN 420, ASP 421, PHE 422, THR 423, PRO 424, LEU 425, SER 426, ASP 427, MET 428, ARG 429, ALA 432, ALA 433, TYR 434, ARG 435, MET 436, ASN 437, ALA 438, ALA 439, GLN 440, ALA 441, ALA 443, LEU 444, ARG 445, TYR 446, VAL 447, ARG 448, GLU 449, LEU 450, GLU 453, VAL 455 |
|  | TNF | 7JRA | VAL 93, ALA 94, PRO 96, ARG 107, ALA 109, ASN 110, LEU 218, ASP 219, PHE 220, GLU 222, SER 223, GLY 224, GLN 225, VAL 226 |

**Table T5.** Docking-based free energy values (△Gb) and interacting residues

| **Ligand** | **PDB ID** | **^△^Gb (kcal/ mol)** | **3D confirmation** | **2D confirmation** | **Ligand interaction with an Amino acid** | **Spacing (Å) Centre grid box** |
| --- | --- | --- | --- | --- | --- | --- |
| Baicalin | 4M0E | -6.93 | 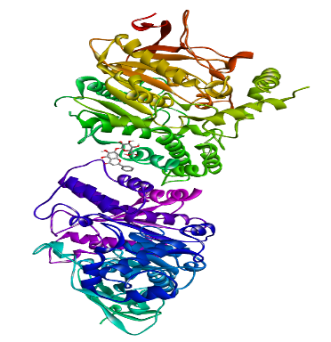 | 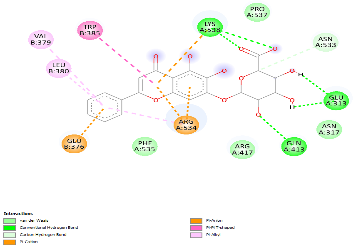 | 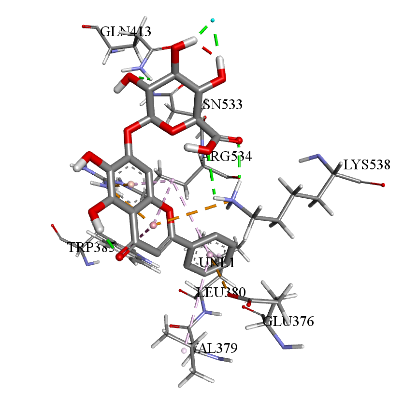 | x centre= 2.029; y centre= -41.752; z centre= 12.701 |
|  | 6F7R | -8.04 | 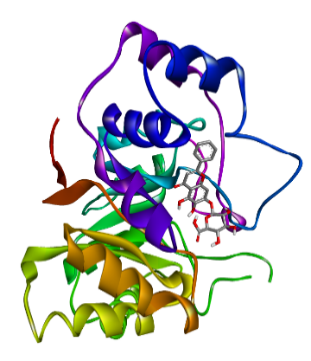 | 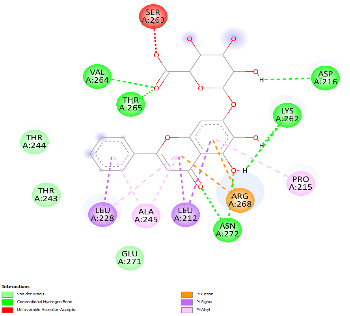 | 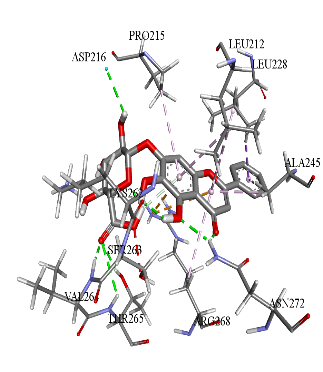 | x centre= 23.242; y centre= 4.817; z centre= 3.587 |
|  | 6Y74 | -7.99 | 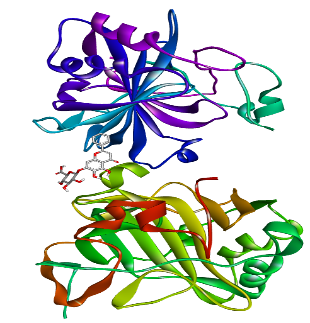 | 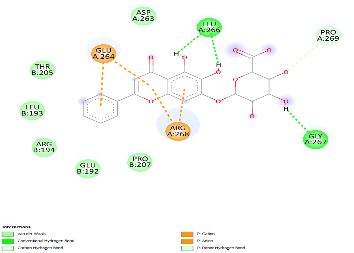 | 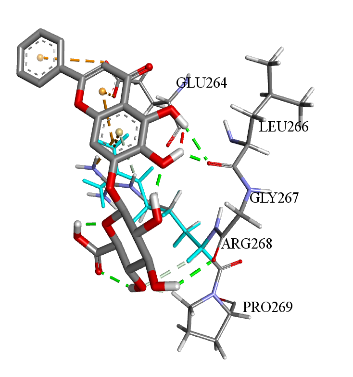 | x centre= -2.496; y centre= 17.152; z centre= 45.681 |
|  | 6CKZ | -7.82 | 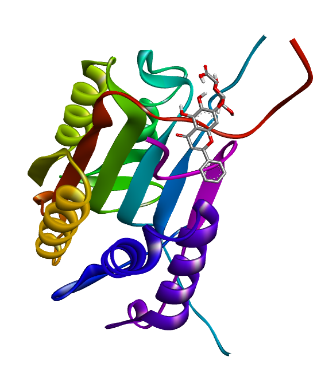 | 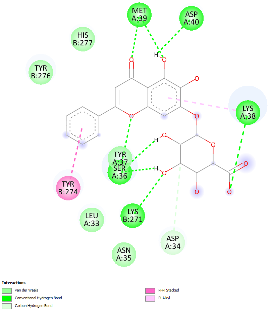 | 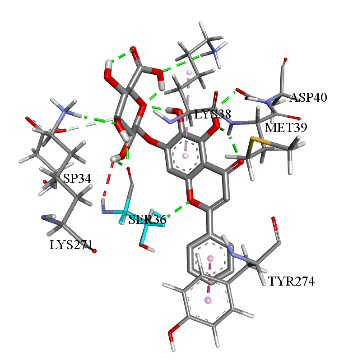 | x centre= 20.126; y centre= 37.556; z centre= 44.77 |
|  | 6BZ9 | -6.93 | 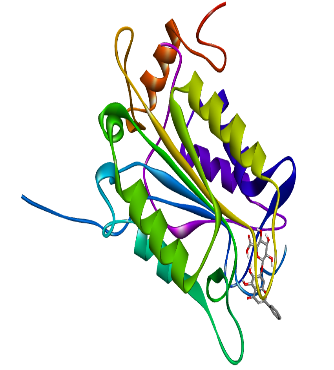 | 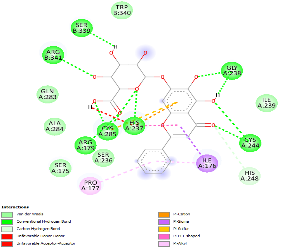 | 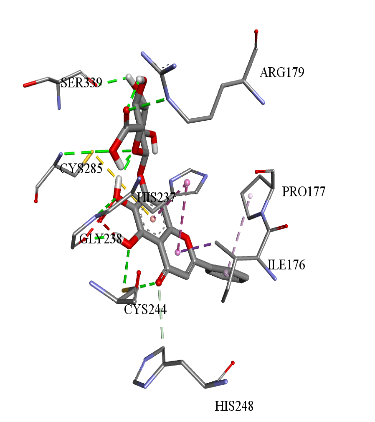 | x centre= 1.636; y centre= -27.072; z centre= 3.865 |
|  | 6CL1 | -7.56 | 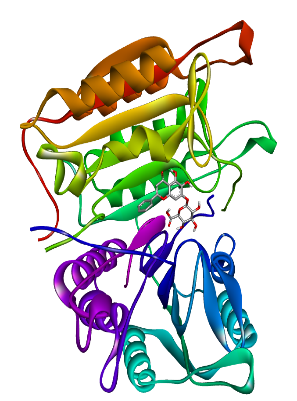 | 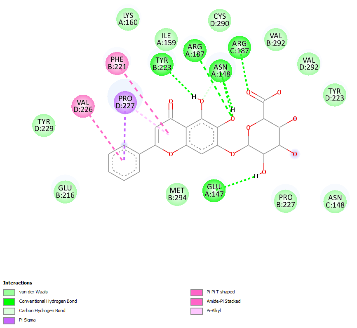 | 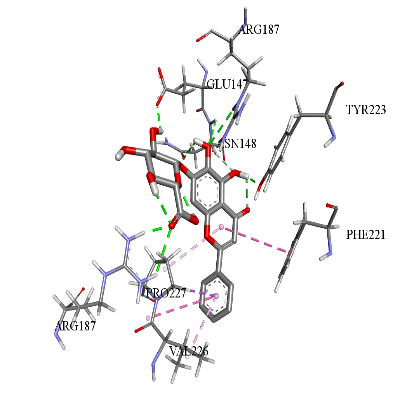 | x centre= 52.626; y centre= 10.582; z centre= 4.309 |
|  | 3KJQ | -6.45 | 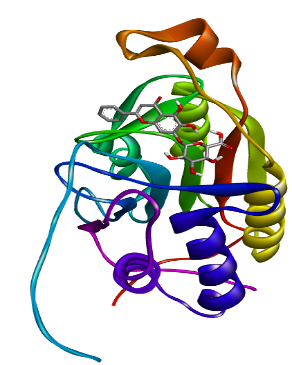 | 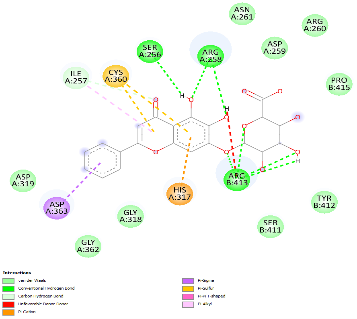 | 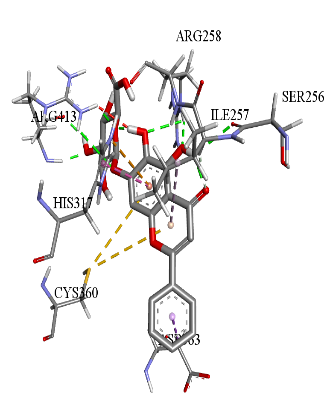 | x centre= -11.504; y centre= 38.267; z centre= 39.32 |
|  | 1RF7 | -8.70 | 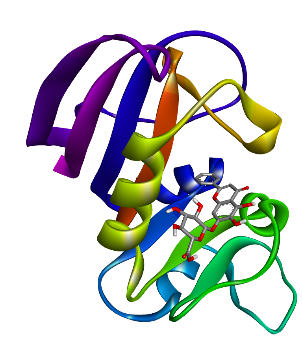 | 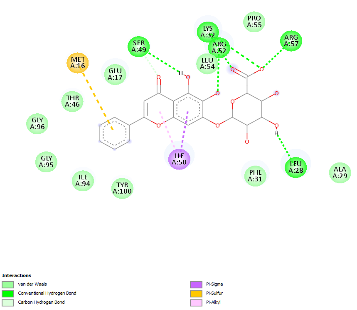 | 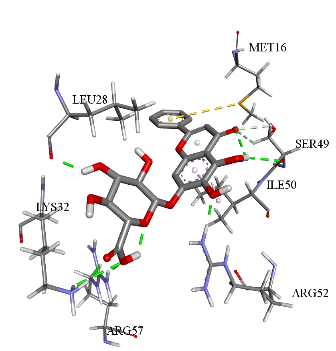 | x centre= 31.638; y centre= 41.214; z centre= 9.542 |
|  | 8OM6 | -5.86 | 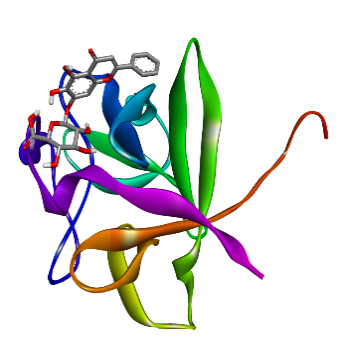 | 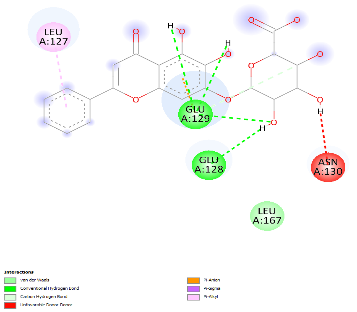 | 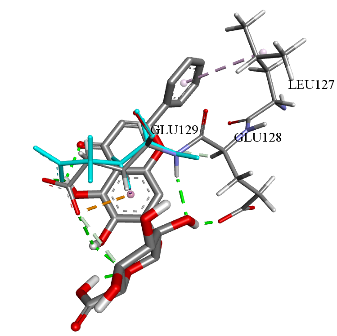 | x centre= -26.467; y centre= -12.289; z centre= 7.104 |
|  | 1MEN | -7.24 | 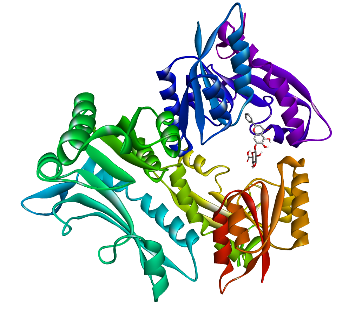 | 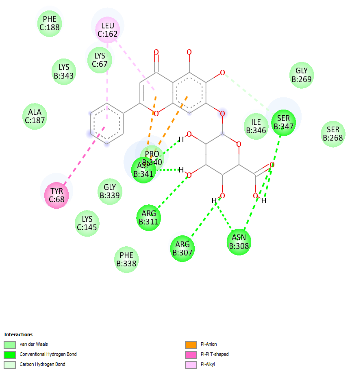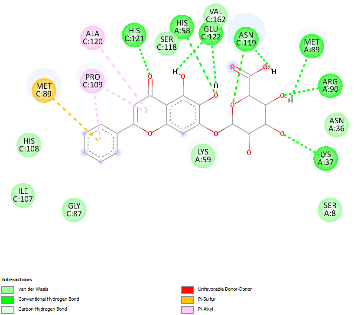 | 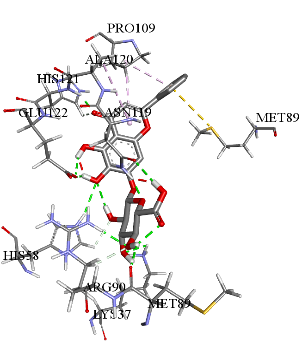 | x centre= -0.728; y centre= 32.724; z centre= 312.679 |
|  | 7LTG | -6.96 | 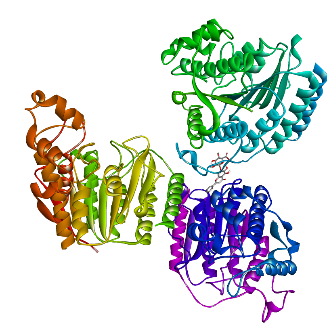 |  | 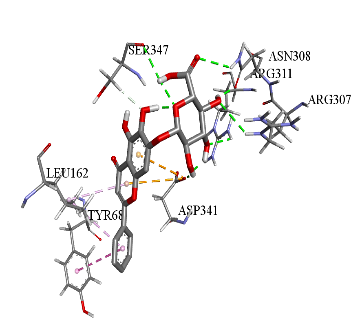 | x centre= 62.897; y centre= 26.375; z centre= -30.374 |
|  | 2QLY | -8.25 | 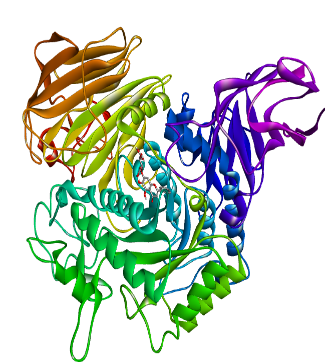 | 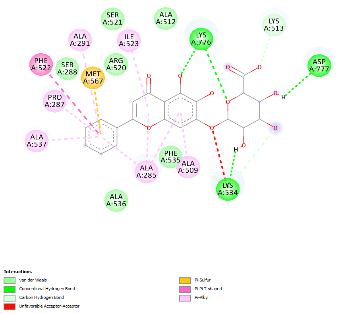 | 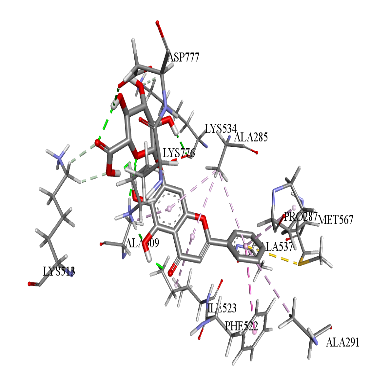 | x centre= 49.429; y centre= 110.095; z centre= 19.145 |
|  | 3SHI | -9.62 | 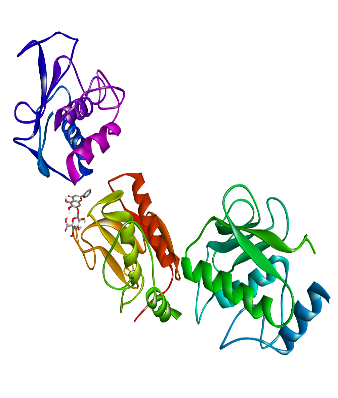 | 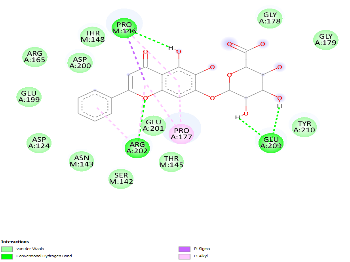 | 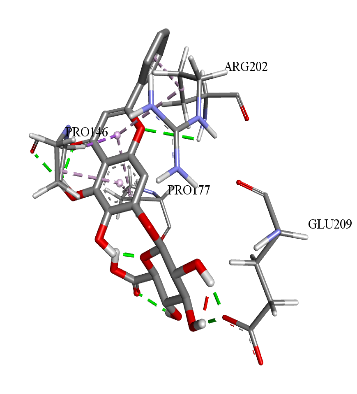 | x centre= 4.527; y centre= -17.936; z centre= -9.359 |
|  | 5LAR | -10.03 | 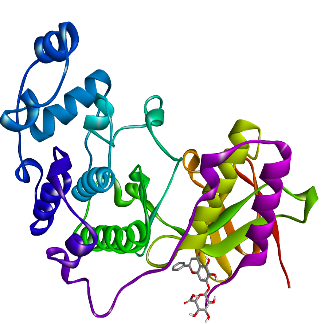 | 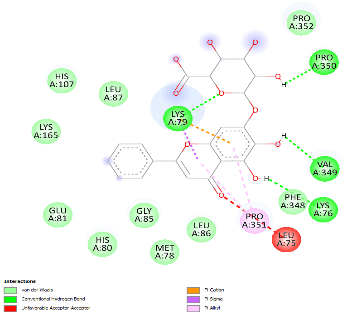 | 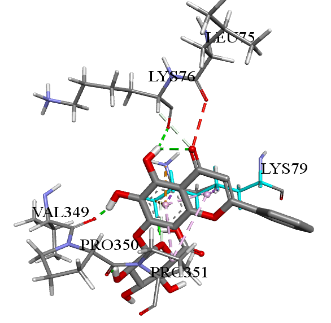 | x centre= 52.412; y centre= 64.78; z centre= 20.218 |
|  | 5F1A | -7.05 | 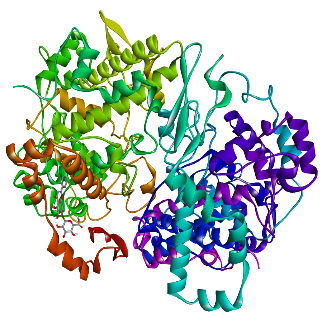 | 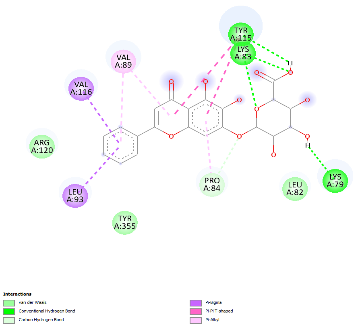 | 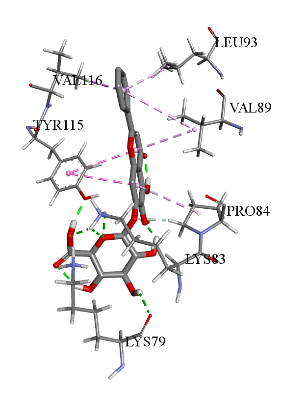 | x centre= 36.86; y centre= 23.708; z centre= 219.777 |
|  | 5X66 | -8.08 | 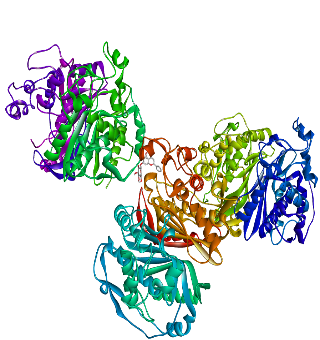 | 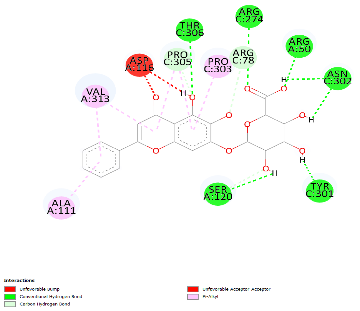 | 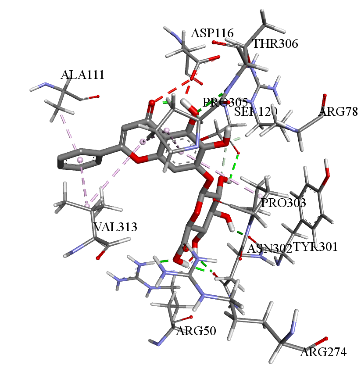 | x centre= 137.742; y centre= 155.864; z centre= 33.166 |
|  | 4FYQ | -5.27 | 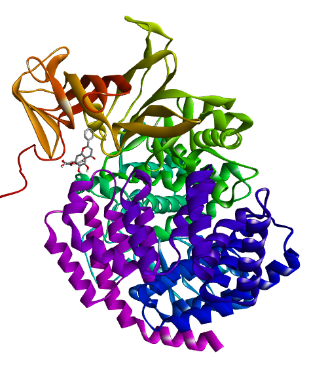 | 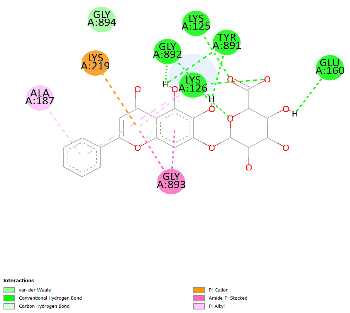 | 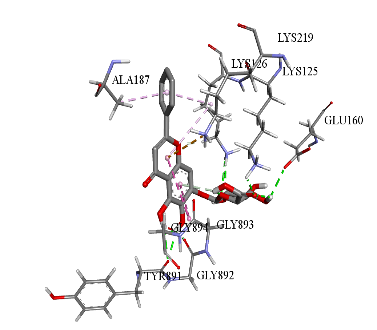 | x centre= -30.691; y centre= -11.362; z centre= 31.17 |
|  | 4WXX | -5.92 | 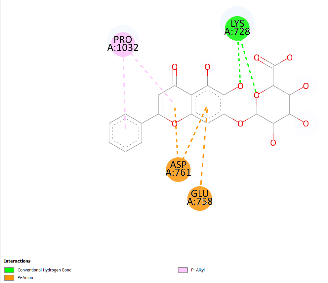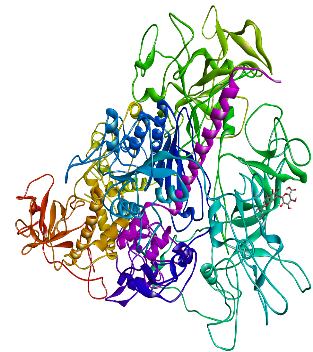 |  | 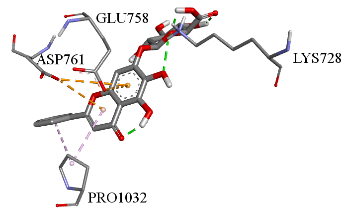 | x centre= -20.888; y centre= 41.559; z centre= 3.095 |
|  | 4WKQ | 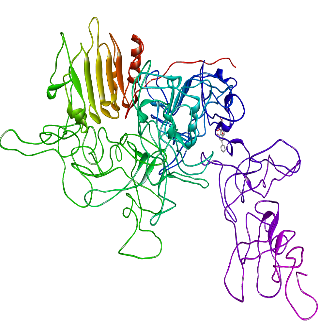-3.51 |  | 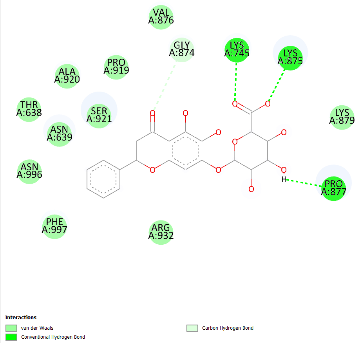 | 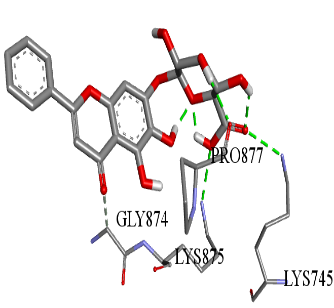 | x centre= 170.105; y centre= 166.234; z centre= 188.573 |
|  | 3PHD | -6.83 | 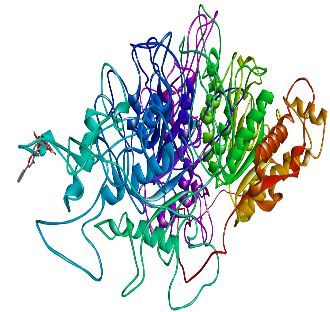 | 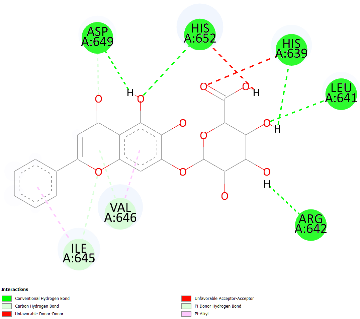 | 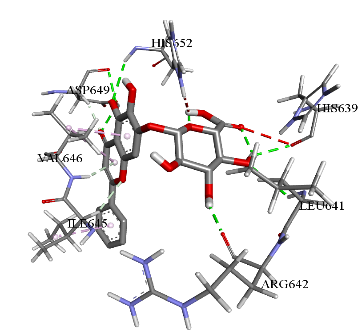 | x centre= 32.781; y centre= -11.03; z centre= 59.973 |
|  | 1Z7G | -6.89 | 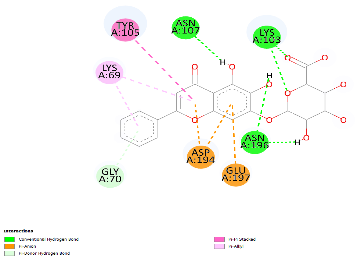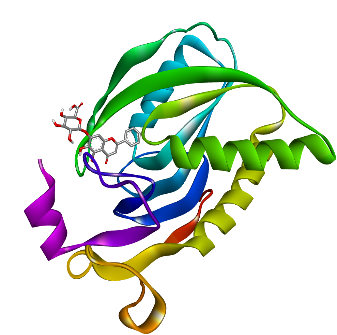 |  | 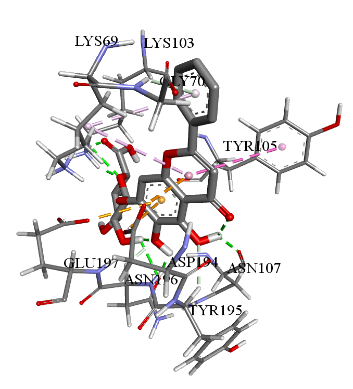 | x centre= 4.277; y centre= 81.255; z centre= 45.595 |
|  | 2D0T | -6.85 | 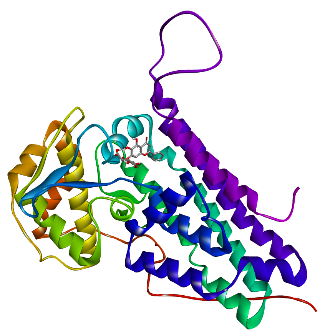 | 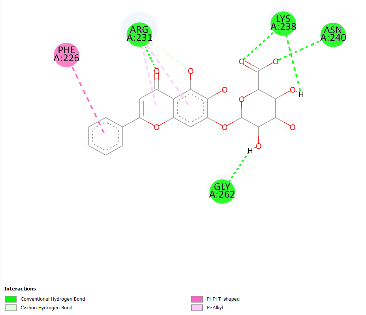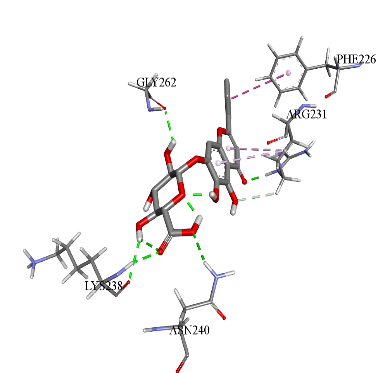 |  | x centre= 15.868; y centre= -7.582; z centre= -27.195 |
|  | 1M47 | -5.23 | 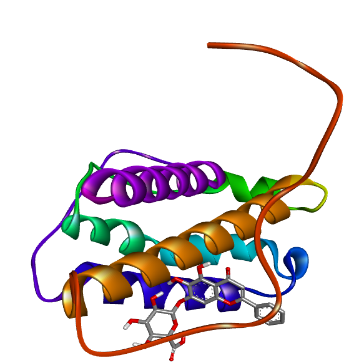 | 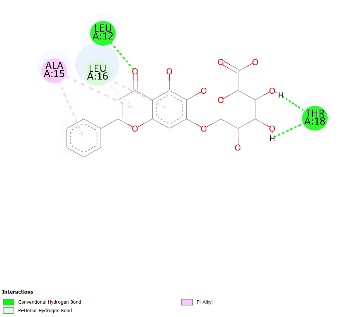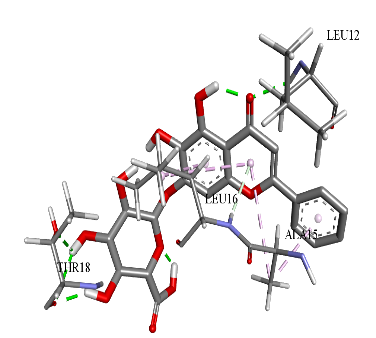 |  | x centre= -7.282; y centre= -61.742; z centre= 102.193 |
|  | 2W3S | -6.77 | 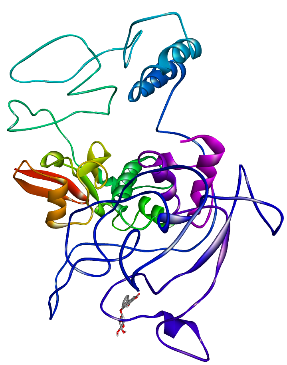 | 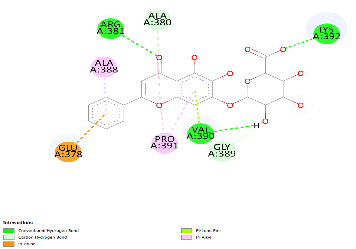 | 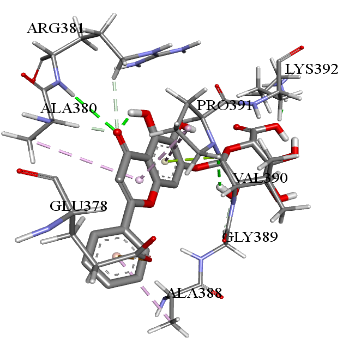 | x centre= 9.882; y centre= 26.907; z centre= 19.658 |
|  | 7JRA | -6.72 | 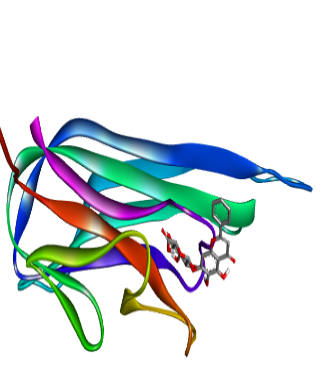 | 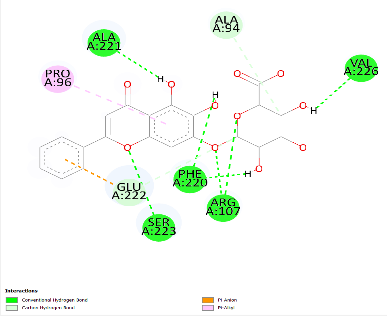 | 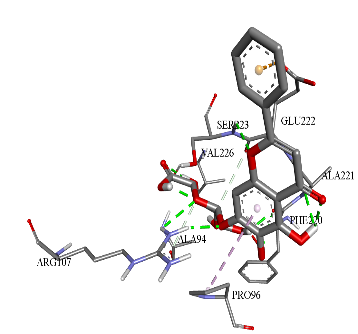 | x centre= 2.314; y centre=-14.496; z centre= 22.417 |
